# Statewide multi-year wastewater sequencing reveals dual origins of HIV-1 signal

**DOI:** 10.1038/s41467-026-74140-7

**Published:** 2026-06-11

**Authors:** Justin R. Clark, Dylan Chirman, Harihara Prakash, Austen Terwilliger, Marlene McNeese, Matt Ross, Mike Tisza, Sara J. Javornik Cregeen, Loren Hopkins, Jennifer Deegan, Catherine L. Troisi, Eric Boerwinkle, Kristina Mena, Fuqing Wu, Jason T. Kimata, Marc Johnson, Devon Gregory, Faith E. Fletcher, Thomas P. Giordano, Anthony W. Maresso

**Affiliations:** 1 Department of Molecular Virology and Microbiology, Baylor College of Medicine, Houston, TX USA; 2 TAILΦR Labs, Baylor College of Medicine, Houston, TX USA; 3 Houston Health Department, Houston, TX USA; 4 Alkek Center for Metagenomics and Microbiome Research, Baylor College of Medicine, Houston, TX USA; 5 The University of Texas Health Science Center at Houston (UTHealth) School of Public Health, Houston, TX USA; 6 Department of Molecular Microbiology and Immunology, University of Missouri-School of Medicine, Columbia, MO USA; 7 Center for Medical Ethics & Health Policy, Baylor College of Medicine, Houston, TX USA; 8 Department of Medicine, Baylor College of Medicine, Houston, TX USA

**Keywords:** Viral epidemiology, Water microbiology, Bioinformatics, Classification and taxonomy, Metagenomics

## Abstract

Human immunodeficiency virus 1 (HIV-1) is a retrovirus which has infected 90 million people and resulted in over 40 million deaths. Despite advances in diagnostics, treatment, and prophylaxis, HIV-1 continues to spread due to undiagnosed and untreated infections. Traditional monitoring methods are ineffective when access to testing is limited or people do not seek care, particularly given the long period between infection and symptom onset, allowing undetected transmission to continue. Here, we use a hybrid-capture sequencing approach to track HIV-1 signal in municipal wastewater in 15 different cities over nearly 3 years. We obtain near-complete genomic coverage of HIV-1, enabling detailed genomic analysis. Surprisingly, there are a substantial number of research-associated retroviral vector sequences recovered. Using computational competitive mapping, we identify specific genomic regions that differentiate authentic HIV-1 from vector-derived inputs. In an exploratory analysis of sites with available clinical data, wastewater-derived circulating HIV-1 reads show a positive correlation with community-level HIV diagnosed prevalence that was robust to exclusion of individual high-prevalence sites. This study identifies lentiviral vector contamination as a confounding factor in wastewater HIV-1 detection, recovers authentic circulating HIV-1 signal through an original classification framework, and provides initial evidence that the resulting signal tracks community HIV burden.

## Abstract (web summary)

Surveillance of infectious diseases through wastewater genomics can help reveal undetected transmission. Here, the authors use wastewater sequencing across 15 Texas cities and computational approaches to track HIV-1 and to distinguish community-derived HIV signal from widespread lentiviral vector contamination.

## Introduction

Human immunodeficiency virus (HIV) has infected nearly 90 million people and caused over 40 million deaths since its identification as the cause of AIDS in the early 1980s^1^. Despite major advances in diagnostics, treatment, and prevention, HIV transmission persists, with 1.3 million new infections globally in 2023^2^ and ~ 38,000 new diagnoses in the United States in 2022^3^. A key barrier to controlling HIV is the difficulty of identifying and engaging individuals who are undiagnosed or not receiving consistent care^4^. An estimated 13% of people with HIV in the US are undiagnosed, and nearly 40% of individuals with HIV are not virally suppressed despite diagnosis and treatment availability^5,6^. These groups—undetected or disengaged from care—account for more than 80% of new transmissions^7^.

Current HIV surveillance methods rely heavily on clinical diagnoses and viral load reporting, which depend on individuals accessing care. As a result, people who do not seek or delay seeking medical attention remain largely invisible to public health systems. Platforms like AIDSVu.org^8^ offer important geographic insights but are limited to reported diagnoses, failing to capture real-time transmission dynamics or silent outbreaks in underserved areas. There is an urgent need for a complementary surveillance strategy capable of detecting undiagnosed or untreated infections at the population level with high spatiotemporal resolution.

Wastewater-based epidemiology (WBE) may offer such potential. First developed for poliovirus surveillance in the mid-20^th^ century^9^, WBE has recently been revitalized through its success in tracking SARS-CoV-2 at the population and community levels^10–19^. Early studies detected HIV-1 nucleic acids in wastewater as far back as the 1990s^20^, and recent work has begun to explore HIV-1 detection using both PCR and sequencing-based approaches^21–23^. However, targeted amplicon strategies face limitations in source attribution and strain resolution, particularly when conserved genomic regions overlap between circulating strains and synthetic constructs used in research settings. Wastewater analyses have accurately mirrored case rates, predicted hospitalizations, and enabled early detection of viral variants^24–28^. The approach is now being extended to other viruses, including mpox and polio virus^29–34^, and forms the basis for the CDC’s National Wastewater Surveillance System for seasonal respiratory pathogens^35^.

Nearly all viral-WBE is performed using PCR. However, this method limits detection capability by focusing on one or a few viral targets. Since May 2022, the Texas Wastewater and Environmental Biomonitoring (TexWEB) group has implemented weekly-to-monthly agnostic pan viral sequencing of wastewater across major Texas cities^24,36,37^. Using a combined hybrid-capture and metagenomic approach^11^, this program has now detected over 400 human and animal viruses in wastewater^38^, enabled monitoring and early detection of viral outbreaks including avian influenza virus^39^, mpox^34^, and measles^40^, and generated a sequencing-based tracking dashboard for public health reporting (SeqBoard: https://dashboard.tephi.texas.gov/public-dashboard). In this study, we apply agnostic hybrid-capture sequencing to detect and characterize HIV-1 in municipal wastewater across 15 Texas cities over nearly three years. We recover near-complete genomic coverage of HIV-1 and identify a large presence of lentiviral vector sequences that confound the interpretation of the wastewater HIV-1 signal. To address this, we develop a competitive alignment framework that classifies reads as circulating (community-derived), non-circulating (vector-derived), or shared based on nucleotide identity to curated reference genomes. We show that without this classification framework, vector contamination obscures any relationship between wastewater signal and community HIV burden, whereas filtering for circulating reads reveals a significant positive correlation with diagnosed HIV prevalence. These findings establish a foundation for sequencing-based HIV wastewater surveillance and highlight lentiviral vector contamination as an overlooked confounding factor that must be addressed in future monitoring efforts for HIV-1 and other pathogens with synthetic counterparts.

## Results

### Widespread and persistent detection of HIV-1 in wastewater

**Fig. 1:**
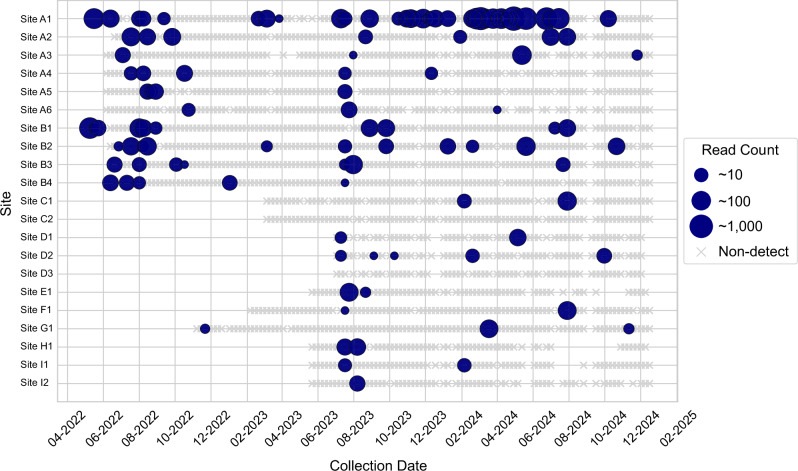
Weekly HIV-1 detection by site in Texas wastewater samples. Each point represents a wastewater sample collection from a specific site during a given week. Marker size is scaled continuously by the log_10_-transformed read count (i.e., 10, 100, 1000) for EsViritu-classified reads that additionally mapped to one of the 149 genomes in our genomic database, while gray ‘x’ markers indicate samples in which no HIV-1 reads were detected. Sites where sampling did not start until 2024 and had no positive samples are not shown. Source data are provided as a Source Data file.

**Fig. 2:**
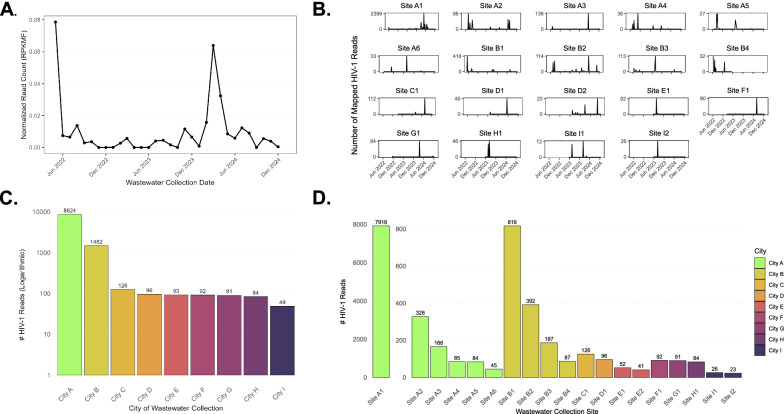
Sustained detection of HIV-1 in wastewater across time, sites, and cities in Texas. **A** Mean HIV-1 signal across all wastewater sites over time, measured as RPKMF, from May 2022 to December 2024. **B** Temporal profiles of EsViritu-classified HIV-1 read counts across individual wastewater sites, labeled by city (A-I) and site number. **C** Cumulative HIV-1 read counts by city. **D** Total read counts by site, color-coded by city. Source data are provided as a Source Data file.

To evaluate whether HIV-1 can be reliably detected using unbiased viral metagenomics, we analyzed longitudinal wastewater samples collected by TexWEB from urban catchment sites across the state between May 2022 and December 2024 (Fig. 1). Aggregated across all sites, we observed a sustained signal of HIV-1, with fluctuations in abundance over time (Fig. 2A). Across 40 wastewater sites in 15 Texas cities sampled 2,086 times between May 2022 and December 2024, HIV-1 reads were detected in 123 (5.9%) of samples. Site-level analysis revealed that several locations contributed disproportionately to HIV-1 read counts (i.e., the number of sequence reads), with certain sites (e.g., Site A1) showing recurrent high-level detection across the study period (Fig. 2B). All sites sampled more than 66 times had at least one positive sample (total range of sampling: 1–142 samples per site; range for positive sites: 66–142 samples per site). Detection frequency varied substantially by site: 6 sites had equal to or less than 3% positive samples (range: 1.5–3.0%), 7 sites had between 3.1 and 5% positive samples, 3 sites had > 5.1% positive samples over the study period (Fig. 2). Site A1 accounted for the majority of HIV-1 signal, with reads detected in 37 of 122 (30.3%) separate sampling events and a maximum single-sample read count of 2399. This concentration of signal at Site A1, located near major medical research infrastructure, is consistent with the predominant detection of vector-associated sequences (see below). Geographic distribution of HIV-1 reads by city and site are shown in Fig. 2C, D, respectively, illustrating heterogeneity in detection intensity across the state. These findings demonstrate that HIV-1 can be detected from municipal wastewater using hybrid-capture sequencing, and that signal is detectable in a geographically and temporally distributed manner. Since sequencing is the approach used herein, we embarked on a deeper analysis of wastewater genomic information with a focus on strain diversity and signal origin.

### Near complete sequencing coverage across the HIV genome

**Fig. 3:**
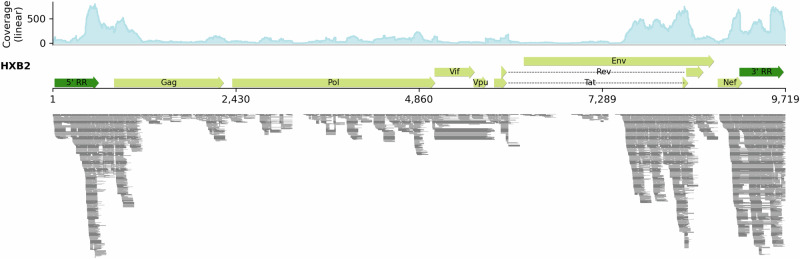
Genome-wide coverage of HIV-1 from wastewater-derived reads via hybrid-capture sequencing. Genome-wide read coverage from all EsViritu-classified HIV-1 reads aligned to the HXB2 reference genome (accession: K03455). Histogram (**top**) shows depth of coverage across the genome; stacked reads (**bottom**) indicate read distribution. Coverage spans 96.4% of the genome, with a mean coverage depth of 136.4x, and enrichment at the 5’ and 3’ long terminal repeat regions (RR) and the 3’ end of *env*. Source data are provided as a Source Data file.

To evaluate the extent of genomic information recovered from wastewater-derived HIV-1 reads, we mapped all reads to the HXB2 reference genome (accession: K03455), a well-characterized, non-circulating strain frequently used in laboratory studies. This alignment revealed broad genome-wide coverage, with mapped reads spanning 96.4% of the HXB2 Genome, with all major viral genes represented (Fig. 3). Further inspection revealed an interesting finding; read coverage was uneven, with a specific enrichment at the 5’ and 3’ LTRs and the 3’ end of the env gene. Because of this finding and the high coverage, we performed a phylogenetic and strain-level analysis.

### Wastewater-derived HIV-1 reads map to both circulating and non-circulating lineages

To explore the genetic diversity of HIV-1 reads detected in wastewater, we performed a phylogenetic analysis using a curated reference database containing 144 full-length subtype B HIV-1 genomes from the Los Alamos National Laboratory (LANL) HIV sequence database alongside five well-characterized non-circulating strains: HXB2, NL4-3, Bru, Yu-2, and NY5. LANL isolates were restricted to one genome per patient and limited to sequences collected in North America since 2014 to reflect contemporary diversity. The five reference strains were selected as historical reference strains because they were isolated in the early HIV epidemic and are widely used in research. For the purposes of this analysis, we refer to these five strains as “non-circulating”.

**Fig. 4:**
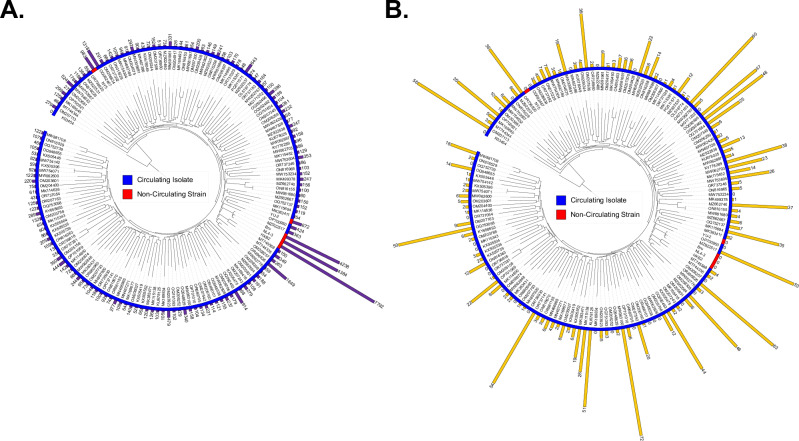
Read mapping across HIV-1 phylogeny highlights circulating and non-circulating lineages. A maximum-likelihood phylogenetic tree of 149 HIV-1 subtype B genomes, including 144 contemporary circulating isolates from the LANL HIV Sequence Database (North America, 2014—present) and five well-characterized non-circulating reference strains (HXB2, NL4-3, Bru, Yu-2, and NY5). The tree was inferred using IQ-TREE with *env* gene alignments (MAFFT). Colored bars indicate the number of wastewater-derived reads multimapping to each reference genome. **A** All mapped reads. **B** Reads that align preferentially to circulating isolates. Source data are provided as a Source Data file.

Maximum-likelihood phylogenetic inference revealed that most non-circulating reference clones clustered into a distinct lineage, separate from the majority of contemporary clinical isolates (Fig. 4). NY5 was a partial exception, exhibiting greater divergence. To investigate read distribution, we aligned wastewater-derived reads to this reference set using a multimapping strategy, in which reads were assigned to any site with an equally high alignment score. Notably, a substantial number of reads mapped preferentially to non-circulating reference genomes, while others aligned more closely to contemporary LANL isolates. A third group aligned equally well to both categories, likely reflecting conserved regions. These conserved regions are termed “shared” in this analysis. To visualize the phylogenetic distribution of reads mapping, we overlaid read multimapping data onto the phylogenetic tree. Figure 4A displays all mapped reads, while Fig. 4B shows only those that aligned more closely to circulating isolates.

Among the 16,710 HIV-1 classified reads, 780 mapped to the genomes of circulating isolates with higher nucleotide identity than to those of non-circulating strains. These reads provide strong, unambiguous genetic evidence of a signal originating from strains circulating within human populations. In contrast and of interest, the high number of reads mapping to non-circulating reference genomes suggests that wastewater may also contain inputs from other sources, including clinical or research-associated retroviral constructs. This distinction between community-derived HIV-1 and other retroviral sources underpins the need for careful interpretation in wastewater surveillance efforts.

### Differential read mapping reveals mixed origins of HIV-1 signal in wastewater

To better characterize the origin of wastewater-derived HIV-1 signal, we quantified how individual reads aligned to circulating and non-circulating reference genomes. Reads were assigned to one of three categories: (1) *circulating*, aligning preferentially to a contemporary LANL isolate; (2) *non-circulating*, aligning best to a historical reference strain; and (3) *shared*, aligning equally well to both. Reads in the third category typically mapped to conserved genomic regions and were treated conservatively in downstream analyses to avoid overinterpretation. This classification was based on the percent identity of each read to its best-aligned reference from each category. A circulating-to-non-circulating identity ratio (C/N ratio - defined as the percent nucleotide identity to the best-matching LANL genome divided by the identity to the best-matching non-circulating reference) was calculated for all reads with special handling of edge cases (e.g., reads with gaps or negative percent identity values; see Methods) and used to distinguish ambiguous signal from more informative, strain-skewed reads.

**Fig. 5:**
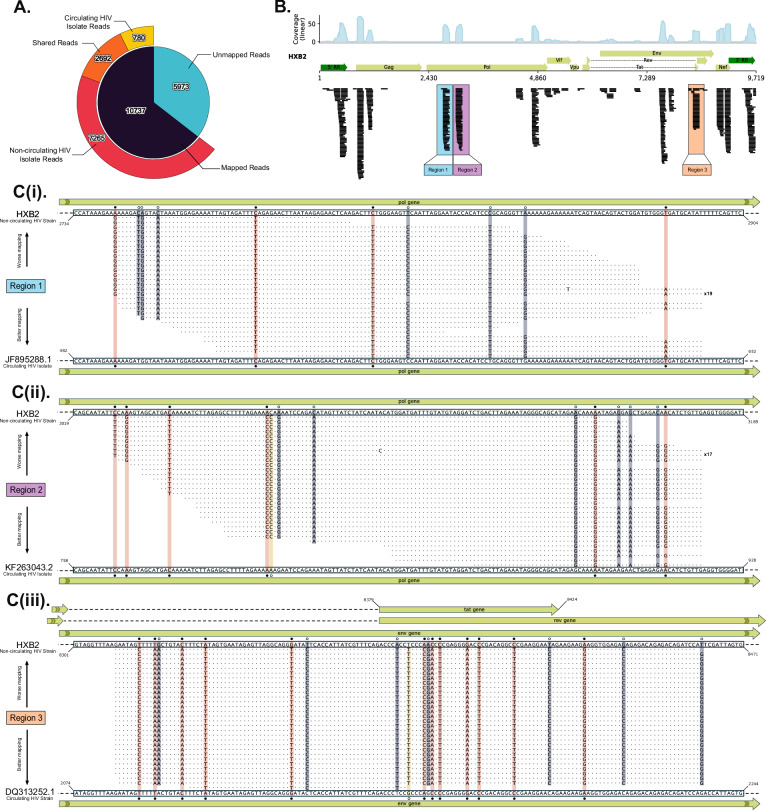
Evidence for circulating HIV-1 signal in wastewater read alignments. **A** Breakdown of read counts based on alignment preferences” “circulating” reads map preferentially to contemporary LANL isolates; “non-circulating” reads align best to historical references (e.g., HXB2); “shared” reads align equally to both. **B** Genome-wide distribution of circulating-aligned reads mapped to the HXB2 non-circulating reference genome. Note the sparse but region-specific coverage reflecting exclusion of conserved/shared reads. **C** Representative multiple sequence alignments (MSAs) from three high-coverage regions in (**B**), comparing read mismatches to both a non-circulating reference and a best-hit circulating isolate from NCBI (accessions: JF895288.1, KF263043.2, DQ313252.1). Closed circles (orange highlight) denote mismatches relative to both references; open circles highlight mismatches exclusive to one reference genome (gray = non-circulating, yellow = circulating.) Duplicate reads in track C(i) and C(ii) are collapsed for clarity. Source data are provided as a Source Data file.

Out of the 16,710 total HIV-1 reads classified by EsViritu in sensitive mode, 10,737 (64%) aligned to one or more references in our curated genome set. Of these mapped reads, 780 (7.3%) aligned preferentially to contemporary LANL isolates (circulating), 7265 (67.7%) aligned best to non-circulating reference strains, and 2692 (25.1%) aligned equally well to both categories (shared; C/N = 1) (Fig. 5A). The remaining 5973 EsViritu-classified reads (36%) did not map cleanly to any genome in the reference set, primarily due to short sequence length after quality trimming (median length: 151 bp) or mischaracterization by EsViritu in sensitive mode, which limits the mappable sequence available for competitive alignment. These unmapped reads were excluded from circulating vs. non-circulating classification and aggregate HIV-1 detection metrics shown in Figs. 1, 2.

To validate the accuracy of this classification framework, we simulated short-read sequences from three circulating isolates, one non-circulating clone, and a representative lentiviral vector. These reads were processed identically to the environmental samples and consistently mapped back to their source genomes, supporting the specificity of C/N ratio-based classification (Supplemental Figs. 2 and 3).

To further contextualize reads categorized as circulating, we examined their distribution across the HXB2 genome. As expected, coverage was sparser than the full HIV-1 read pool (Fig. 5B), given that shared reads—often derived from conserved regions—were excluded. We selected three genomic regions with relatively high coverage from circulating reads and compared their alignments against both non-circulating references and top BLAST hits from the NCBI database (Fig. 5C). In all cases, pairwise identity was greater with the circulating reference, supporting the classification. Notably, all three regions originated from the same wastewater site (A2), which is situated in a high-HIV-burden area according to AIDSvu^8^. This region was originally included in sampling because of its relatively high HIV burden and strongly suggests these three genomic regions are unambiguously assigned to circulating strains and should be considered for probe or primer design for more specific, targeted assays.

While these 780 circulating reads represent a small proportion of the total HIV-1 signal in wastewater, their consistent detection across multiple samples, sites, and timepoints supports their biological relevance. The relatively low read depth observed for this class limited genome reconstruction and may challenge finer-scale variant analysis under current enrichment and sequencing conditions.

**Fig. 6:**
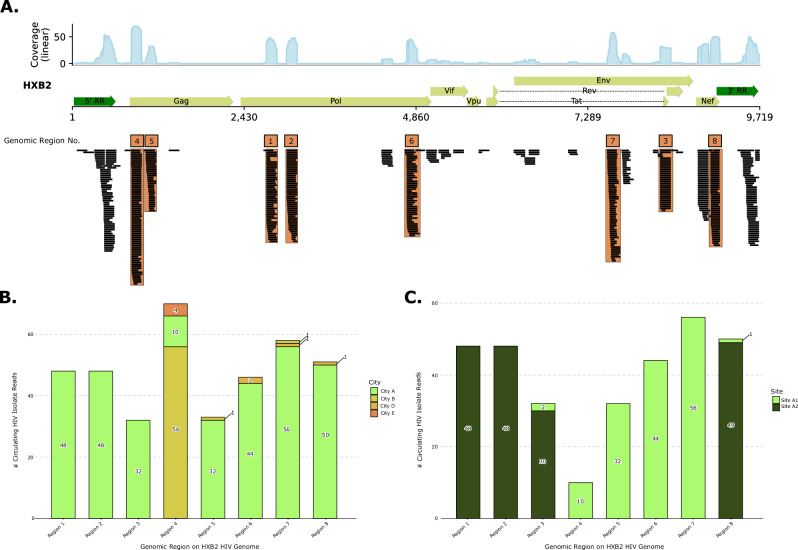
Circulating HIV-1 wastewater reads are geographically and genomically distributed. **A** Wastewater-derived HIV-1 reads that aligned preferentially to circulating reference strains were mapped across the HXB2 genome. Read depth (top track; blue) is shown above gene annotations, with individual reads (black bars) grouped into eight predefined genomic regions (orange boxes, labeled 1–8). **B** Stacked bar graph showing total circulating read counts per genomic region, colored by contributing city. City A dominates several high-coverage regions. **C** Bar plots showing the same data as (**B**) but split by two major wastewater sites within City A. Site A1 and A2 are the predominant sources for multiple regions. Site A2 is of particular interest because it contained relatively few non-circulating reads, suggesting localized enrichment of circulating HIV-1 signal. Source data are provided as a Source Data file.

To further evaluate the spatial and genomic distribution of circulating HIV-1 reads, we divided the HIV-1 genome into eight major regions and quantified read counts within each. This segmentation revealed that circulating reads were not evenly distributed: certain regions showed higher coverage, suggesting localized hotspots of genetic diversity with minimal overlap with non-circulating variants (Fig. 6A). Site-level analysis showed that two sampling locations, Site A1 and Site A2 within City A, contributed disproportionately to these high-coverage regions (Fig. 6B, C), with circulating reads recovered on 25 sampling dates from Site A1 and 4 sampling dates from Site A2 over the two-year study period. This site could inform targeted public health outreach.

All three genomic regions previously highlighted in Fig. 5 originated from Site A2, a catchment area that lacks major healthcare infrastructure but has one of the city’s highest estimated HIV prevalence rates according to AIDSVu. This convergence of genetic signal, site consistency, and epidemiological data strongly suggests that these reads reflect bona fide shedding from people with HIV in the community rather than environmental contamination or clinical runoff. Although City A exhibited the highest overall count of circulating reads (Supplemental Fig. 4), several other urban sites also yielded modest signals. This pattern indicates that while City A may represent a surveillance hotspot, lower-level circulation signals are detectable across a broader geographic area.

Collectively, this classification framework supports a mixed origin for the HIV-1 signal in wastewater: some reads most likely represent true circulating virus, while others may derive from synthetic or research-associated retroviral sources. To internally assess classification fidelity, we simulated Illumina reads from two circulating HIV-1 genomes and one non-circulating reference (HXB2). When aligned and visualized phylogenetically, the reads clustered around their source genomes, confirming that our mapping approach preserves strain-level identity (Supplemental Figs. 2–3). These results support the ability of the pipeline to distinguish HIV-1 source lineages in silico. This circulating vs. non-circulating distinction also provides a critical foundation for interpreting wastewater-based HIV surveillance.

### Spatiotemporal patterns of HIV-1 reads support authentic wastewater origin

**Fig. 7:**
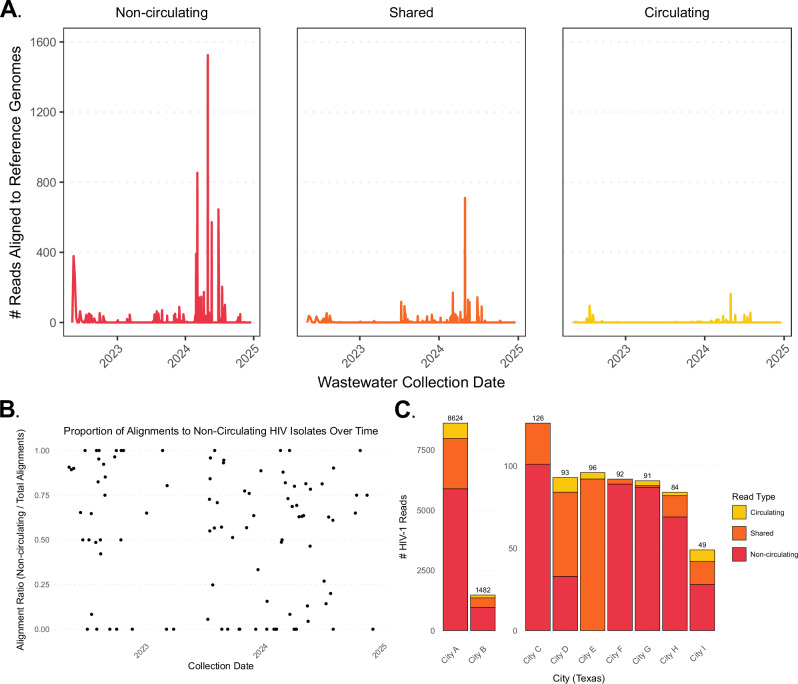
Spatiotemporal trends in circulating, shared, and non-circulating wastewater-derived HIV-1 reads. **A** Total read counts classified as circulating, shared (equal identity to both strain classes), or non-circulating, plotted over the full sampling period (May 2022-December 2024). Peaks in detection are shared across categories. **B** Proportion of reads aligning best to non-circulating strains over time, showing no abrupt shifts indicative of laboratory contamination. **C** Distribution of read types across individual sampling sites, illustrating consistent ratios of circulating, shared, and non-circulating reads across geographic locations. Source data are provided as a Source Data file.

To determine whether the frequent detection of HIV-1 reads—particularly those mapping to non-circulating strains—might result from laboratory contamination, we examined their temporal and spatial distribution across sampling sites. We first plotted HIV-1 read counts over time, categorized by mapping preference: non-circulating strains, circulating isolates, or shared alignments (Fig. 7A). This analysis revealed multiple peaks in HIV-1 detection during the first half of 2024 with elevated read counts spanning several wastewater catchment sites and multiple cities (Fig. 2B), suggesting these signals were not isolated events.

We further assessed whether specific dates showed disproportionate alignment to non-circulating strains, which might suggest episodic contamination. No such temporal clustering was observed (Fig. 7B). In addition, our sequencing laboratory is CLIA-certified, does not process clinical HIV samples, and does not perform HIV-related research—further supporting that the observed signal is unlikely to arise from in-lab contamination.

Next, we examined HIV-1 read abundance by site and city across the study period (May 2022—December 2024). While Site A1 exhibited the highest overall read count (Fig. 2D), and City A the highest aggregate signal (Fig. 2C), reads aligning to non-circulating versus circulating strains were proportionally distributed across sites and cities (Fig. 7C). No spatial bias was evident in terms of the type of strain detected.

However, site-level analysis revealed stark contrasts within the same city. Site A1, which had the highest overall HIV-1 read count, contributed 5631 of the 7265 total non-circulating reads (∼ 78%)—over 13 times more than its 409 circulating reads—suggesting a dominant vector-associated signal. In contrast, Site A2 contributed just 64 non-circulating reads but 191 circulating-classified reads (Supplemental Fig. 4). This divergence supports the interpretation that wastewater HIV-1 reads reflect inputs from both synthetic and biological sources, with source composition varying substantially even between catchments in the same city.

Interestingly, Site A2 contributed 191 circulating-classified reads from 4 sampling events over 2 years, compared to Site A1’s 409 circulating reads from 25 sampling events. Critically, Site A2’s ratio of circulating to non-circulating reads (191:64, or 3:1) was inverse to Site A1’s ratio (409:5631, or 1:14), indicating minimal vector contamination at Site A2 despite its location in a high HIV-prevalence area according to AIDSVu data.

While our read classification framework helps distinguish between circulating and vector-derived signals, the underlying detection of HIV-1 itself is agnostic to source. We therefore define detection sensitivity in terms of total HIV-1 read recovery. Although we did not perform a formal dilution series or spike-in calibration, we consistently detected HIV-1 signal in samples with as few as 2 total classified reads, corresponding to approximately 0.005 RPKMF under current enrichment and sequencing conditions. These empirical thresholds may serve as provisional benchmarks for interpreting future wastewater surveillance datasets until formal limit-of-detection experiments are conducted.

Taken together, these observations argue against localized contamination events as the source of HIV-1 sequences in wastewater. Instead, the reproducible detection of both circulating and non-circulating HIV-1 reads across multiple time points and geographically distinct sites supports the interpretation that these signals reflect true wastewater-derived nucleic acid inputs, likely from diverse sources.

### Circulating HIV-1 reads correlate with ZIP code level HIV prevalence

To evaluate whether reads classified as circulating reflect epidemiologically meaningful signal, we performed an exploratory correlation analysis between wastewater-derived read counts and publicly available HIV prevalence data from AIDSVu for Cities A, B, D, and H, the four cities for which ZIP code-level data were available. Prevalence estimates represent year-end 2023 point prevalence of persons living with diagnosed HIV per 100,000 population (See “Methods”). We compared correlations using either all HIV-1 reads or only reads classified as circulating (C/N ratio > 1) to assess whether our classification framework improves correlation with clinical data.

Site A1 presents a unique analytical challenge: located at a major medical research center, this site exhibited overwhelming vector contamination with 5631 non-circulating (vector-derived) reads versus only 409 circulating reads, a 14:1 ratio. For all-reads analysis, including Site A1, would render the correlation biologically uninterpretable, as the signal would be dominated by research-associated vectors rather than community shedding. However, for circulating-read analysis, Site A1 provides a critical test of whether our classification framework can successfully extract authentic HIV signal even in heavily contaminated environments.

**Fig. 8:**
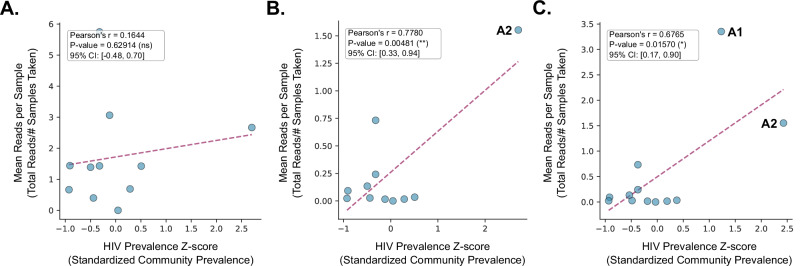
Correlation between wastewater-derived HIV-1 reads and community-level diagnosed HIV prevalence. Wastewater read counts (mean reads per sample) were compared with the year-end 2023 point prevalence of diagnosed HIV per 100,000 population (AIDSVu/CDC) for Cities A, B, D, and H. Prevalence values are expressed as z-scores calculated across the sites shown in each panel to preserve anonymity. **A** All HIV-1 reads with Site A1 excluded (*n* = 11 sampling sites). No significant correlation was observed (Pearson’s *r* = 0.16, two-tailed *p* = 0.63, 95% CI [− 0.48, 0.70], suggesting vector-derived reads obscure the epidemiological signal. **B** Circulating reads only (C/N ratio > 1) with Site A1 excluded (*n* = 11 sampling sites). Filtering for circulating reads revealed a significant positive correlation (*r* = 0.78, two-tailed *p* = 0.0048, 95% CI [0.33, 0.94]). **C** Circulating reads with Site A1 included (*n* = 12 sampling sites). Despite high vector contamination at Site A1 (14:1 non-circulating:circulating read ratio; 64.9 mean reads per sample), the correlation remained significant (*r* = 0.68, two-tailed *p* = 0.016, 95% CI [0.17, 0.90]). Dashed lines represent linear regression fits. *p < 0.05, **p < 0.01. Source data are provided as a Source Data file.

Circulating read classification substantially improved correlation with HIV prevalence compared to using all reads (Fig. 8). When Site A1 was excluded from both analyses (to enable fair comparison), all reads showed no significant correlation (Pearson’s *r* = 0.16, *p* = 0.629, 95% CI [− 0.48, 0.70], *n* = 11 sampling sites) (Fig. 8A), while circulating reads showed a significant positive association (*r* = 0.78, *p* = 0.0048, 95% CI [0.33, 0.94], *n* = 11 sampling sites) (Fig. 8B). This contrast is notable because without our classification framework, vector contamination completely obscures any relationship between wastewater HIV signal and community prevalence, whereas filtering for circulating reads reveals a significant correlation. When Site A1 was included in the circulating reads analysis to test the performance of our circulating read quantification framework, the correlation remained statistically significant (*r* = 0.68, *p* = 0.0157, 95% CI [0.17, 0.90], *n* = 12 sampling sites) (Fig. 8C), suggesting that the competitive alignment framework can extract epidemiologically informative signal even in catchments serving major biomedical research facilities.

To further assess the robustness of these correlations, we performed additional sensitivity analyses (Supplemental Fig. 5). When analyzed independently, City A (*n* = 4 sampling sites; excluding the heavily contaminated A1) and City H (*n* = 1 sampling site, within the metropolitan area of City A) showed stronger correlation with circulating reads (*r* = 0.92, *p* = 0.027, 95% CI [0.20, 0.99], *n* = 5 sampling sites) than all reads (*r* = 0.87, *p* = 0.055, 95% CI [− 0.05, 0.99], *n* = 5 sampling sites) (Supplemental Fig. 5A, B).

The correlation was not dependent on any single high-burden site. When Site A2 (the highest HIV-prevalence catchment) was excluded but Site A1 (the most vector-contaminated site) was retained, the correlation among circulating reads from both cities remained significant (*r* = 0.72, *p* = 0.013, 95% CI [0.21, 0.92], *n* = 11 sampling sites) (Supplemental Fig. 5C). This is the most direct test of the concern that the overall correlation might be driven by a single high-leverage point, and the results indicate that the association persists without it.

To formally assess site-level influence, we computed Cook’s distance for all sites in the full circulating reads analysis (Fig. 8C, *n* = 12). Two sites exceeded the conventional threshold (0.33): Site A2 (Cook’s *D* = 1.90) and Site A1 (Cook’s *D* = 1.17); no other site exceeded the threshold (all remaining Cook’s *D* < 0.06). These sites were influential for biologically distinct and interpretable reasons. A2 exhibited high leverage due to its position at the upper extreme of the prevalence distribution, anchoring the regression slope as the most informative observation for detecting a gradient, which is the expected pattern when a genuine biological relationship exists, but only one catchment represents the high-prevalence end of the range. Site A1 exhibited a large positive residual (standardized residual = 2.84), consistent with residual vector contamination inflating its circulating read count above the value predicted by prevalence alone, which explains the improvement in correlation strength when A1 is excluded (Fig. 8B; *r* = 0.78) compared to the full dataset (Fig. 8C; *r* = 0.68).

Adequate dynamic range in HIV prevalence was necessary to detect the correlation. When both sites with elevated prevalence (A1 and A2) were excluded, the correlation became non-significant (*r* = − 0.13, *p* = 0.72, 95% CI [− 0.70, 0.55], *n* = 10 sampling sites) (Supplemental Fig. 5D). This is consistent with a range restriction effect where the remaining sites compressed into a narrow prevalence band, there is insufficient variation to detect a trend, rather than evidence against a biological relationship. Across all sensitivity scenarios retaining adequate dynamic range, the correlation was consistently positive and significant (*r* = 0.68-0.92, all *p* < 0.05), supporting the interpretation that wastewater-derived circulating HIV reads track with community HIV burden.

### Vector-derived sequences drive coverage peaks in HIV-1 genome

To investigate the source of high read coverage across certain regions of the HIV-1 genome, we examined the alignment patterns of wastewater-derived HIV-1 reads against the HXB2 reference genome. We observed several chimeric reads in which one portion aligned to HIV-1, while the remainder showed no homology to the genome, suggesting potential vector-derived artifacts.

To better classify these sequences, we employed a BLAST-based pipeline querying the NCBI nucleotide, patent, and synthetic databases. Reads were categorized into four groups based on their top BLAST hits: HIV-only, synthetic-only, other (non-HIV), and ambiguous (aligning to more than one combination of datasets). This analysis revealed that a substantial fraction of wastewater reads initially classified as HIV-1 via competitive mapping also aligned to lentiviral vector sequences. These sequences commonly originate from backbones incorporating regions from HIV-1 strains like HXB2 and NL4-3.

**Fig. 9:**
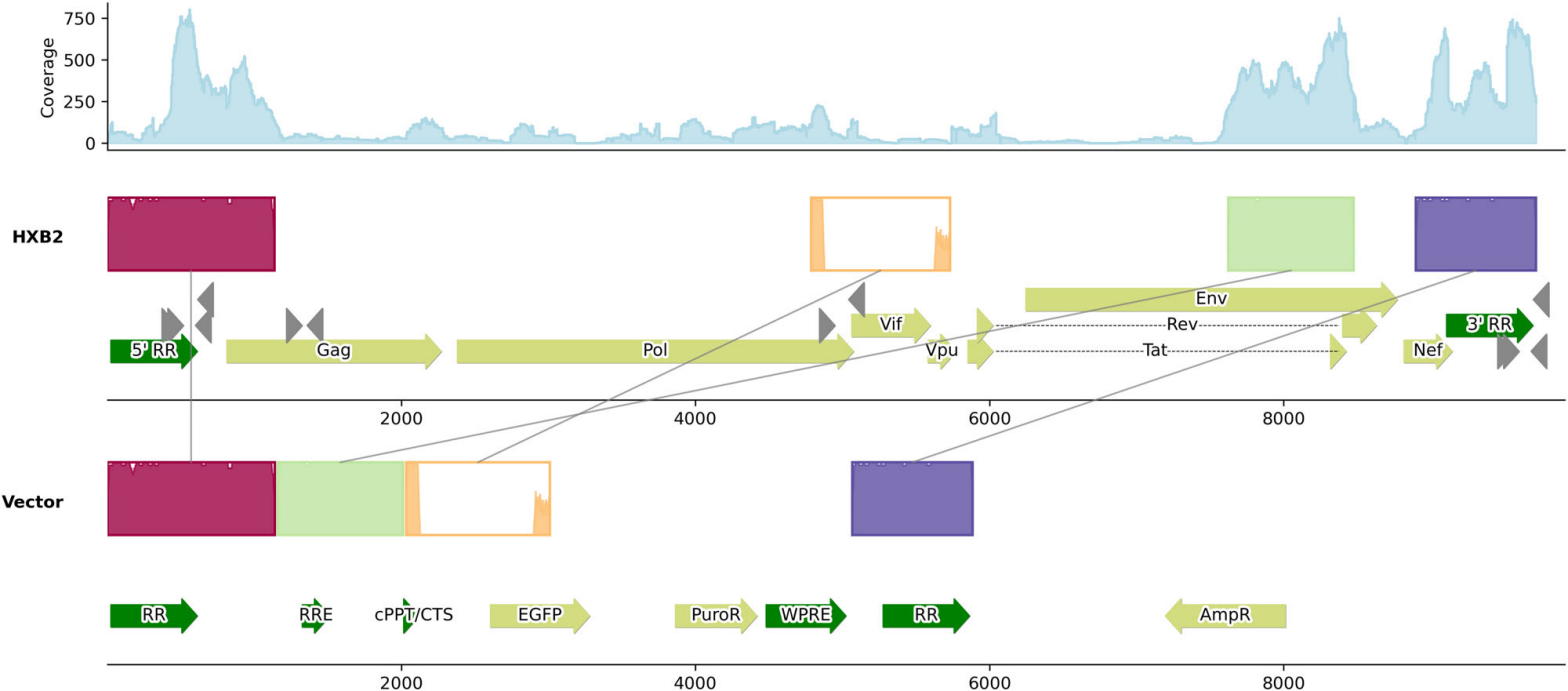
Genomic overlaps between wastewater HIV-1 reads and a lentiviral vector. Top panel: Coverage depth (y-axis) of wastewater-derived HIV-1 reads mapped against the non-circulating HXB2 genome (x-axis), annotated with major HIV-1 genes (gold arrows), long terminal repeat regions (RR, dark green arrows), and primers that have previously been used in wastewater work (gray arrowheads). Colored boxes identify regions of high coverage. Bottom panel: Representative lentiviral vector annotated with key elements: Rev Response Element (RRE), central polypurine tract/central termination sequence (cPPT/CTS), Enhanced Green Fluorescent Protein (EGFP), puromycin resistance gene (PuroR), Woodchuck Hepatitis Virus Posttranscriptional Regulatory Element (WPRE) and ampicillin resistance gene (AmpR). Connecting gray lines denote homologous segments between the vector and HXB2 genome, indicating the possible presence of wastewater HIV-1 reads by non-infectious lentiviral vectors. Source data are provided as a Source Data file.

Coverage peaks were particularly enriched in the terminal repeats, the pol gene, and the 3’ end of env—regions frequently retained in lentiviral vector constructs. Additional reads mapped exclusively or predominantly to synthetic elements, further supporting a vector-based origin for many of the high-depth reads. To visualize these overlaps, we plotted the genomic coverage of HIV-1 reads against HXB2 and aligned this with a representative lentiviral vector genome (Fig. 9). Regions of high read depth corresponded to structural and regulatory vector components such as the 5’ and 3’ LTRs, cPPT/CTS and RRE.

We also separately visualized the coverage from reads classified as synthetic-only, which did not align to the HIV-1 genome but did align to known lentiviral vector constructs. These reads mapped precisely to junctions between synthetic backbones and HIV-derived regions, further implicating lentiviral vectors as the source (Supplemental Fig. 6). We further annotated Fig. 9 to include the primer sites (gray arrowheads) used in both our prior preprint and the recent Wolfe et al.^21^ study on HIV-1 detection in wastewater. Notably, these primer sites are predominantly located in regions of the genome that showed the highest coverage in our sequencing data—regions that also strongly overlap with lentiviral vector components such as the 3’ env gene and the LTRs. This observation suggests that previous PCR-based studies may have unintentionally targeted genomic regions enriched by vector-derived nucleic acids, complicating the interpretation of past detections and reinforcing the need for approaches capable of differentiating endogenous HIV-1 from synthetic retroviral material, such as the sequencing approach used herein.

## Discussion

This study demonstrates a comprehensive application of unbiased wastewater sequencing to detect and genetically characterize HIV-1 across multiple urban catchments over a nearly three-year surveillance period. By systematically distinguishing between community-derived circulating strains and research-associated lentiviral vectors, we establish a robust analytical framework for wastewater-based epidemiology (WBE) and demonstrate its potential to supplement existing HIV surveillance systems—particularly in identifying communities with undiagnosed or untreated infections.

Building on our team’s early work in wastewater surveillance for SARS-CoV-2—which demonstrated that viral levels in sewersheds could serve as a leading indicator for clinical outbreaks^10,36,41^—we developed a hybrid-capture sequencing pipeline for virome-wide monitoring in collaboration with the Texas Epidemic Public Health Institute (TEPHI). This approach enables simultaneous detection of DNA and RNA viruses, including both enveloped and non-enveloped viruses from municipal wastewater^11,38^. Leveraging this platform, we have previously tracked community dynamics of mpox^34^, avian influenza^39^, and measles^40^. As a natural extension of this framework, we investigated whether HIV-1, an enveloped RNA virus with substantial public health impact, could be reliably detected and characterized using this virome-wide approach.

Across > 2000 samples from 15 cities, we classified over 16,000 reads, identified genomic hotspots enriched for vector-associated signal, mapped site-specific variation in signal type and abundance, and aligned detected circulating isolates with known epidemiological trends. These combined insights form the basis for a scalable, high-resolution WBE platform capable of discriminating clinical from synthetic signal with direct relevance to public health action.

HIV-1 is known to shed in urine, stool, and genital secretions, and early studies demonstrated its detection in wastewater via RT-PCR as far back as the 1990s^20,42–46^. Studies have shown that HIV RNA/DNA is detected in fecal samples from 8–19% of chronically infected individuals with detectable plasma viral loads, but is not detected in subjects on antiretroviral therapy (ART) with undetectable plasma viral loads^45^. This shedding originates from gut-associated lymphoid tissue (GALT), a major HIV reservoir that releases infected cells and free virus into the intestinal lumen. While this selectivity might initially seem problematic for surveillance, it actually enhances public health utility. Individuals with detectable viral loads, whether untreated, unsuppressed, or undiagnosed, represent the primary drivers of HIV transmission and are precisely the populations that traditional clinical surveillance struggles to capture.

WBE does not require universal or uniform shedding across infected individuals; it requires only that sufficient shedding occurs within populations served by the sewershed to generate a detectable signal. The enrichment of wastewater detection towards virally unsuppressed individuals aligns surveillance with epidemiological need. This principle is supported by SARS-CoV-2 wastewater surveillance, which succeeded despite substantial individual variation in shedding by disease stage, treatment status, and symptom severity. Future validation studies correlating wastewater measurements with clinical data will help clarify detection sensitivity across different populations, but the fundamental principle remains: population-level signal enriched for transmission-competent infections provides actionable surveillance value.

However, despite the early promise, few studies have pursued HIV wastewater surveillance and the ones that have relied on targeted PCR assays that amplify short, conserved regions^21,45,46^. These approaches face two major limitations. First, many primer sets target genomic regions conserved across both circulating HIV-1 strains, non-circulating HIV-1 strains, and lentiviral vectors, complicating source attribution. Second, targeted amplicon strategies inherently bias detection towards specific genomic loci, limiting insights into strain diversity and origin. Our sequencing-based approach addresses these constraints by enabling genome-wide, untargeted detection—improving resolution and enabling discrimination between clinical and alternative sources.

To disentangle these sources, we implemented a competitive alignment strategy that classifies reads as circulating, non-circulating, or shared based on identity ratios against curated circulating HIV-1 references and commonly used non-circulating molecular clones. This method revealed two major read classes: authentic circulating strains likely reflective of community-level HIV, and synthetic constructs overwhelmingly associated with known vector backbones like HXB2 and NL4-3. These categories were not evenly distributed. Some sites—such as Site A2—yielded circulating reads closely related to contemporary isolates and comparatively low levels of reads categorized as non-circulating. Other sites, most notably Site A1, were dominated by vector-associated sequences, contributing over 75% of the total non-circulating signal in the dataset. This skewed signal strongly suggests persistent environmental input from biomedical facilities (or perhaps gene therapy centers) rather than incidental lab contamination, particularly given Site A1’s proximity to major medical research infrastructure.

Because lentiviral vectors are subject to strict regulatory disposal protocols, they have not typically been considered a source of confounding HIV-1 signal in wastewater. This assumption has likely contributed to the lack of vector screening in prior studies. However, our results indicate that vector-derived nucleic acids are consistently detectable in wastewater, particularly at sites near biomedical research infrastructure. It is not possible to determine from sequence data alone whether these represent intact but inactivated vectors, fragmented genetic material, or degraded nucleic acids persisting after standard decontamination. Accepted decontamination protocols prioritize inactivation of infectious agents over elimination of all nucleic acids, and nucleic acid persistence after chemical or thermal treatment is well-documented^47^. The consistent recovery of vector sequences across multiple time points at research-adjacent sites suggests this reflects research activity rather than isolated contamination event.

The presence of vector-derived sequences represents an important methodological consideration for surveillance interpretation rather than necessarily indicating biosafety violations or environmental health hazards. This overlooked confounding factor may help explain why HIV-1 wastewater detection has historically lagged behind surveillance of other pathogens. Past studies relying on short, conserved PCR amplicons may have inadvertently amplified vector-derived sequences—particularly in regions like pol, env, and the LTRs—without recognizing their synthetic origin. In the absence of phylogenetic or genome-wide context, such signals might reasonably be dismissed as laboratory artifacts, limiting the interpretability and dissemination of early findings.

It is worth noting that commercial HIV wastewater surveillance assays are already available and in use (e.g., GT Molecular), indicating that detection of HIV-related nucleic acids in wastewater, including potential vector-derived sequences, is an established practice. Our sequencing-based approach provides the genomic resolution needed to distinguish vector-derived from circulating signals, a distinction not possible with PCR-only methods, enabling more accurate interpretation of HIV wastewater surveillance data.

The implications of our findings are twofold. First, the detection of lentiviral vector signal complicates public health interpretation and could lead to misidentification of transmission hotspots. However, identifying this signal is also valuable: it provides a basis for developing bioinformatic filters or targeted depletion strategies that can be applied to future wastewater sequencing efforts. Vector-associated sequences were enriched in specific regions of the HIV-1 genome—such as the LTRs and the 3’ end of env and pol—that overlap with commonly used lentiviral backbones. These findings can guide the design of next-generation probes or amplification strategies that avoid known vector hotspots while retaining coverage of clinically informative regions.

Second, the consistent recovery of circulating HIV-1 reads from sites without obvious clinical or research infrastructure highlights the potential of wastewater sequencing to identify underrecognized transmission zones. For example, Site A2, despite lacking a major hospital catchment, yielded the second highest number of circulating reads in the dataset with the highest circulating-to-non-circulating read ratio, and those reads mapped to diverse regions of the HIV-1 genome across multiple time points. A review of public epidemiological data (e.g., AIDSVu) confirms that the zip codes covered by this site harbor a disproportionately high burden of HIV, supporting the inference that wastewater detection at this site reflects genuine, ongoing shedding from people with HIV. These insights could be used to guide targeted testing and intervention campaigns, especially in areas where traditional surveillance is limited.

This study demonstrates proof-of-concept for sequencing-based HIV wastewater surveillance but also reveals important technical challenges. The relatively low recovery of circulating-classified reads (780 of 16,710 total HIV reads, or ~ 5%) reflects both biological constraints, such as variable fecal shedding among people with HIV, and technical limitations of hybrid-capture efficiency for highly diverse viral genomes. On the technical side, hybrid-capture efficiency for diverse HIV fragments remains challenging, especially given the high sequence variability across global isolates. Biologically, the background presence of lentiviral vector DNA may obscure low-abundance authentic signals, particularly in sites near research institutions. In addition, the need to maintain site anonymity, which is particularly important for HIV surveillance given ongoing stigma and criminalization, limits our ability to fully characterize catchment-level factors that may influence signal composition. Future optimization could explore enhanced enrichment strategies, deeper sequencing, or targeted depletion of vector-associated regions to improve signal-to-noise ratios. The limited depth of circulating reads in this dataset precludes high-resolution variant tracking or full-genome reconstruction, which may be essential for fine-grained epidemiological inference in future studies.

We deliberately employed commercially available reagents (Twist Comprehensive Viral Research Panel) and standardized reference databases to assess whether HIV surveillance could be achieved without site-specific customization. While this approach successfully detected and characterized the circulating HIV signal, the sparse recovery suggests that further methodological development is needed before widespread implementation. Potential improvements include supplementing commercial panels with consensus sequences from high-prevalence regions or developing vector-specific blocking probes. The analytical framework established here, particularly the competitive alignment strategy for distinguishing circulating from vector-derived reads, provides a foundation for such optimization efforts.

Another key uncertainty is the biological form of the detected HIV-1 material. Circulating reads could represent intact virions, proviral DNA from lysed cells, or fragmented viral genomes from human excretions. Early work in the 1990s demonstrated that infectious HIV could not be recovered from wastewater, likely due to viral inactivation during treatment processes or environmental degradation^20,46,48,49^. Without enrichment for RNA or methods to distinguish between viral particles and nucleic acid debris, we cannot definitively determine whether detected sequences represent infectious virus. Molecular approaches such as strand-specific library preparation, reverse-transcription assays, or long-read sequencing to detect human-HIV chimeric reads may help clarify the nature of the environmental signal. These approaches are critical to interpret wastewater HIV detection in an epidemiologically meaningful way.

Finally, linking wastewater sequencing results to clinical outcomes remains an open challenge. Correlating HIV-1 signal in wastewater with geographic incidence, treatment coverage, or viral suppression rates would enable more confident attribution of the signal to community-level transmission. Such integration is both feasible and necessary: while wastewater provides granular, continuous surveillance data, its public health utility hinges on validation against established clinical metrics. To begin addressing this challenge, we performed an exploratory correlation analysis using publicly available HIV prevalence data from AIDSVu^8^ (Emory University), which aggregates data from the CDC’s National HIV Surveillance System, for sites where ZIP code-level data were available (Cities A, B, D, and H; *n* = 5-12 sites depending on the analysis; Fig. 8 and Supplemental Fig. 5). This analysis tested whether our circulating read classification tracks with known epidemiological patterns. Because AIDSvu reports diagnosed prevalence only, these estimates do not capture undiagnosed infections and may underestimate true community HIV burden, particularly in communities with lower testing access, which could attenuate the observed correlations.

The contrast between the results for all-reads and circulating-reads in the correlation analyses is notable because using all-reads showed no significant correlation with prevalence (*r* = 0.16, *p* = 0.63; Fig. 8A), whereas filtering for circulating reads revealed a significant positive association (*r* = 0.78, *p* = 0.0048; Fig. 8B), suggesting that our classification framework is necessary to extract epidemiologically meaningful signal from the mixed wastewater HIV-1 read pool. Site A1, serving a major medical research center with a 14:1 non-circulating:circulating read ratio, provided a stringent test for our framework. The persistence of a significant correlation when A1 was included (*r* = 0.68, *p* = 0.016; Fig. 8C) suggests that competitive alignment can discriminate circulating reads even under high contamination, with practical implications for monitoring in catchments serving biomedical facilities.

Several limitations of the correlation analysis warrant emphasis. Sample sizes (*n* = 10-12) and Cook’s distance analysis confirmed that Sites A1 and A2 exert disproportionate influence on the regression, as expected given their positions at the extremes of the prevalence and signal distributions. Importantly, the correlation persisted when A2 was excluded (*r* = 0.72, *p* = 0.013; Supplemental Fig. 5C), indicating that the association is not dependent on this single high-prevalence site. In addition, geographic mismatch between sewersheds and administrative units such as ZIP codes introduces spatial uncertainty into prevalence comparisons^50^. In this study, we used population-weighted prevalence estimates computed from the spatial intersection of City (where available) or EPA-modeled sewershed catchment boundaries with Census ZCTA polygons, which reduces but does not eliminate this source of error. The AIDSVu prevalence data reflect year-end 2023 diagnoses rather than contemporaneous viral shedding, and data were available for only four cities. One site serving a major transportation hub was excluded because transient populations violated the assumption that the wastewater signal reflects the local community burden.

The range restriction analysis (Supplemental Fig. 5D) further illustrates that wastewater HIV monitoring requires meaningful variation in HIV burden for statistical detection. When both high-prevalence sites were excluded, the correlation became non-significant (*r* = − 0.13, *p* = 0.72), consistent with insufficient dynamic range rather than contradictory biology. Future studies with larger and more geographically diverse cohorts, longitudinal sampling, and integration of additional clinical metrics such as viral suppression rates will be needed to establish the quantitative relationship between wastewater signal and community HIV burden with greater precision.

Despite these constraints, the consistent observation of significant positive correlations across multiple sensitivity scenarios (*r* = 0.68–0.92, all *p* < 0.05), combined with the absence of any correlation when vector-derived reads are included (*r* = 0.16, *p* = 0.63; Fig. 8A), provides proof-of-concept that wastewater HIV detection tracks with community burden when vector contamination is properly accounted for. We interpret these correlations as encouraging initial evidence rather than definitive validation; the exploratory nature of this analysis, combined with the acknowledged geographic and temporal limitations, warrants cautious interpretation. Nevertheless, the improved concordance using circulating reads versus all reads supports the biological relevance and practical necessity of our classification framework.

Beyond validation, translating detection into actionable public health interventions requires further development. Empirical thresholds linking wastewater signal to community prevalence must be established through longitudinal studies, similar to those conducted for SARS-CoV-2, to determine meaningful alert levels. Our group’s prior work with SARS-CoV-2 surveillance in Houston demonstrated the utility of wastewater-guided interventions^10,41^. For HIV, the advantages could be even more substantial given the lengthy and expensive epidemiological investigations currently required to confirm local outbreaks.

Clear intervention pathways must also be defined in partnership with local health departments and affected communities. Plausible responses to elevated HIV signal could include deployment of mobile testing units to affected catchments, alert to clinical facilities and providers in the affected areas to increase HIV testing and be vigilant for acute retroviral syndrome, intensified PrEP outreach, and strategic resource allocation. The key advantage lies in detecting transmission among undiagnosed populations—the groups most responsible for ongoing transmission—potentially weeks to months before traditional surveillance systems would identify an outbreak, and with minimal additional resources.

If successful, this approach could offer health systems a real-time, population-scale method to identify communities with high levels of undiagnosed or unsuppressed HIV-1—particularly in areas underserved by traditional surveillance. This study represents an early but critical step toward that goal, establishing an unbiased, scalable framework for detecting and evaluating HIV-1 signal through comprehensive wastewater virome sequencing. While substantial work remains to translate technical feasibility into operational readiness, the foundational analytical framework presented here provides a basis for future development.

We also cannot overlook the fact that HIV wastewater surveillance raises ethical considerations distinct from other pathogens. HIV remains subject to criminalization laws in many U.S. jurisdictions, and people living with HIV continue to face stigmatization and discrimination. There is ongoing debate within HIV advocacy communities about genomic surveillance conducted without explicit consent^51,52^, and these realities demand that HIV surveillance approaches, including wastewater methods, include robust safeguards against misuse and harm to affected communities.

Our methodology does not enable individual-level identification. The circulating HIV reads we recovered are highly fragmented, represent pooled community signal from entire sewersheds, and reflect substantial within-host viral heterogeneity^53^. Even with future improvements in sensitivity, extensive biological and technical barriers to individual-level surveillance remain. Nevertheless, as sequencing-based wastewater surveillance capabilities advance, clear ethical frameworks are essential^51,52^. Wastewater-derived HIV data should focus exclusively on population-level public health benefits and should not be used for individual identification or legal purposes.

We recommend that HIV wastewater surveillance programs partner with community-based HIV organizations and advocacy groups to address concerns about privacy, stigma, data stewardship and distrust^54^. Our team conducted townhalls with people living with HIV to gather input on study design, data use, and community concerns. These partnerships help ensure surveillance practices align with community needs and can advocate for expanding access to wastewater monitoring^54^. Continued community engagement is necessary to realize public health benefits while preventing harm to affected communities.

Taken together, this study provides a methodological and conceptual framework for the high-resolution detection and classification of HIV-1 in complex environmental samples. By moving beyond PCR-based detection and establishing a competitive alignment model to distinguish circulating from vector-associated reads, we demonstrate the feasibility of using wastewater sequencing for near-real-time HIV surveillance at the community level. Importantly, the analytical framework developed here is adaptable. While focused on HIV-1, the same principles can be extended to other pathogens, particularly those with synthetic counterparts or used in laboratory settings. As WBE becomes a cornerstone of global infectious disease monitoring, resolving issues of source ambiguity, classification accuracy, and public health integration will be critical. This work represents a major step toward that goal.

## Methods

### Wastewater collection

Beginning in 2022, untreated influent was collected from 40 wastewater catchment sites across 15 Texas cities on a weekly to monthly basis^11,38^. To protect privacy and minimize potential stigma associated with community-level HIV detection, cities with positive detections were anonymized as Cities A-I and individual sites within those cities as Sites 1-7. Between 100 and 500 mL of influent was sampled using 24 h composite samplers at municipal wastewater treatment plants. Sample bottles were surface-decontaminated with 10% bleach, sealed in sterile bags and shipped on ice to Baylor College of Medicine. Upon arrival, samples were stored at 4 °C and processed within 24 h.

### Hybrid-capture sequencing approach for detection of HIV-1 in wastewater

Wastewater is processed next to extract bulk DNA and RNA from centrifuged solid pellets^38^. RNA is reverse-transcribed to cDNA using the Protoscript II First Strand cDNA Synthesis Kit (New England Biolabs Inc.), NEBNext Ultra II Non-Directional RNA Second Strand Module (New England Biolabs Inc.), and Random Primer 6 (New England Biolabs Inc.) before mixing with DNA for library construction. Libraries are constructed with Twist Library Preparation EF 2.0 Kit and Twist Universal Adaptor System (Twist Biosciences) and pooled with a maximum of 16 samples, totaling 1500 ng. Probes are hybridized with the Twist Comprehensive Viral Research Panel (Twist Biosciences) at 70 °C for 16 h. Sequencing yields raw binary base call (BCL) data files, which are converted to FASTQ format. Demultiplexing is done from dual-index barcodes using Illumina bcl2fastq software (v2.20).

### HIV-1 Sequence assignment with EsViritu

Demultiplexed FASTQ files undergo quality control through processing with BBDuk for quality trimming (Q25). Illumina adapters are trimmed, and reads mapping with BBMap to PhiX Illumina spike-in are filtered. Reads mapping to a human reference genome database (GCF_000001405.39) by BBMap are also removed. The remaining processed reads are classified by our previously published bioinformatics pipeline EsViritu (v0.2.3) using the default settings^38^. Reads were classified as HIV-1 with minimap2 competitive mapping against the Virus Pathogen Database (v2.0.2) with a minimum of 90% identity across at least 90% of the read length. Reconstructed alignments were clustered by sequence similarity to deduplicate sequences with > 98% identity, and the consensus sequence from these clusters were used as the final sequence. Viral species assignments were confirmed by mapping these final extracted read sequences against a restricted database of HIV-1 only genomes from the Virus Pathogen Database (v2.0.2) with the same minimap2 settings and storing reads that were re-classified as HIV-1.

### Taxonomic filtering and abundance averaging via TREx

To extract and summarize HIV-1 signal from EsViritu output, we developed a companion analysis module called TREx (Taxonomic-based Relative-Abundance Extractor, v0.1). TREx filters taxonomic profiles based on accession number, taxonomic IDs, or lineage terms, joins each hit to site-level metadata (including site, city, and collection date), and computes Reads Per Kilobase per Million Filtered Reads (RPKMF) per sample. It supports both standard and sensitive detection outputs and includes options for temporal aggregation at the weekly, monthly, or yearly level.

This script was used to extract and aggregate the data shown in Fig. 2a. All data processing was performed in Python using pandas, with downstream visualizations generated separately in R (v4.3.0) using ggplot. TREx is publicly available on GitHub.

### Detection and removal of library preparation artifacts using FADE

To remove sequencing artifacts introduced during library preparation, we applied the FADE tool (Fragmentase Artifact Detection and Elimination; github.com/blachlylab/fade, v0.6.0) to identify and excise structured chimeric reads containing inverted terminal repeats. These artifacts, typically resulting from enzymatic fragmentation, can resemble bona fide recombination or integration events and are often incompletely filtered by conventional soft-clipping during alignment.

EsViritu-processed reads were first retrimmed using BBDuk (v38.84) to trim low-quality (Q < 20) reads and filter out < 30 bp reads after trimming, resulting in read lengths of 30-151 bp (median = 151 bp). Trimmed and filtered reads were aligned to the HXB2 reference genome (accession: K03455) using minimap2 (v2.26) in local mode with default parameters. HXB2 was chosen because preliminary analysis suggested the more reads mapped to it than other non-circulating strains.

FADE was run in two steps: FADE annotate was used to identify inverted terminal repeats, and FADE out was used to hard-clip these regions. FADE specifically targets terminal segments aligned in reverse orientation to the rest of the read—consistent with the formation of interrupted palindromes during library preparation. This high-specificity filtering preserves the central informative portion of each read while eliminating artifact-derived terminal sequences.

To ensure that the true biological signal was not lost during artifact removal, we treated FADE as a quality control step and mapped reads were extracted from the filtered BAM files for downstream analyses.

### Competitive mapping to all HIV-1 subtypes

To verify that subtype B made up the overwhelming majority of our EsViritu-classified HIV-1 reads, we selected representative reference genomes for all known HIV-1 subtypes (3 isolates for A, C, D, F1, G; 2 isolates for O, and 1 isolates for H, J, K, L, N, O, P, and U subtypes) and used them to create a reference database with our 5 subtype B non-circulating strains (HXB2, NL4-3, Bru, Yu-2, NY5).

Trimmed reads were then mapped to this database of reference genomes using a multimapping strategy with the Geneious Assembler (v2025.0.3). Read counts from BAM files were then tabulated and used to generate a bar chart (Supplemental Fig. 1) using a custom Python script.

### Analysis of mapped reads

To enable comparative classification of wastewater-derived HIV-1 reads, we constructed a curated reference database comprised of complete HIV-1 subtype B genomes isolated from North America between 2014 and 2024. Reference genomes were retrieved from the Los Alamos National Laboratory (LANL) database with the inclusion criteria requiring (1) full-genome coverage, (2) subtype B designation, (3) collection within the last 10 years, (4) no duplicate patients (i.e., one isolate per patient). In total, 144 contemporary circulating sequences meeting these criteria were selected. These were supplemented with five historically important non-circulating reference clones: HXB2, NL4-3, Bru, Yu-2, and NY5.

Trimmed and FADE-processed reads were mapped to the combined reference set using Geneious Assembler (v2025.0.3) with “best-to-all” mapping (multimapping) enabled, which assigns reads to all equally optimal alignment positions. This strategy helps prevent artificial inflation of read counts for any single genome when dealing with highly conserved regions. BAM files were exported from Geneious and further processed using custom Python scripts, starting with samtools (v1.21) to add mismatch information (-NM tag) to each BAM file.

Using the -NM tags, percent identity was calculated as:

1 $${Percent}\,{Identity}\,\left(\%\right)=\frac{{Read}\,{Length}\,-{Number}\,{of}\,{Mismatches}}{{Aligned}\,{Length}}\times 100$$

Where alignment length included both mismatches and gaps. Reads were then categorized as preferentially aligning to either circulating or non-circulating genomes based on which class yielded the highest alignment identity using the Circulating/Non-circulating percent identity ratio heuristic (C/N Ratio):

2 $$C/N\,{Ratio}=\frac{{Percent}\,{Identity}\,{to}\,{Circulating}\,{Isolate}}{{Percent}\,{Identity}\,{to}\,{Non}-{Circulating}\,{Strain}}$$

Due to this method weighing both mismatches and gaps equally, reads containing large gaps (e.g., resulting from recombination events or vector engineering) yielded negative percent identities. To correct this, the following conditions were applied:

- If both the circulating and non-circulating percent identities were negative, the C/N ratio calculation was inverted:

3 $$C/N\,{Ratio}=\frac{{Percent}\,{Identity}\,{to}\,{Non}-{Circulating}\,{Strain}}{{Percent}\,{Identity}\,{to}\,{Circulating}\,{Isolate}}$$

- If the percent identity to the circulating isolate was negative but positive for the non-circulating strain, the C/N ratio was set to 0.1.
- If the percent identity to the non-circulating strain was negative but positive for the circulating isolate, the C/N ratio was set to 2.

Read classifications were then integrated with sample-level metadata using parsed FASTQ headers and per-pool Excel files containing site, city, and collection data for each sample. A comprehensive summary table was generated, listing mapping categories, best-hit strain, percent identity, C/N ratio, and associated sample information for each read. Filtered read sets were also produced to support downstream alignment visualization, coverage profiling, and phylogenetic classification. All outputs were generated using a custom Python script.

### Phylogenetic analysis

To contextualize wastewater-derived HIV-1 reads within the broader genetic diversity of HIV-1 subtype B, we performed reference-based phylogenetic analysis using a curated set of circulating 144 HIV-1 subtype B genomes. Five non-circulating molecular clones—HXB2, NL4-3, Bru, Yu-2, and NY5—were included as internal controls, as described above.

Rather than using whole-genome sequences, we focused the analysis on the *env* gene, a region with sufficient diversity for phylogenetic resolution, yet conserved enough for robust alignment. First, all annotated env genes were extracted in Geneious Prime (v2025.0.3). For genomes lacking annotated env genes, the env open reading frame was predicted using Geneious’s built-in Open Reading Frame Finder. The HIV-1 subtype D strain ELI (accession: K03454) was included as an outgroup.

Sequences were aligned using the command-line version of MAFFT (v7.453) with –localpair and –maxiterate 1000 options to optimize alignment accuracy in hypervariable regions. A maximum-likelihood phylogenetic tree was inferred using IQ-TREE (v 2.1.4) with GTR + G substitution model, 1000 SH-aLRT replications, and 2000 ultrafast bootstrap replicates. The tree topology converged with a final correlation coefficient of 0.99, indicating strong and stable branch support.

Two read-mapping annotation schemes were used to generate iTOL-compatible annotation tracks:

- All reads: each read was counted based on its best-scoring alignment to any reference genome.
- Circulating reads only: a subset combining reads that mapped exclusively to circulating strains or to both categories with a C/N ratio > 1, indicating closer identity to circulating references.

For both annotation tracks, each read was counted for every genome it aligned to in the BAM files. This provided a more complete view of alignment overlap—especially in conserved regions—and informed var chart overlaps in iTOL (7.5.1). A color strip was included to distinguish non-circulating strains (red) from circulating isolates (blue). All outputs were generated using a custom Python pipeline integrating alignment, tree-building, and read classification (available at https://github.com/TAILOR-Lab/hiv-wastewater-study).

### In silico validation of mapping and classification pipeline

To validate the specificity and phylogenetic resolution of our read classification pipeline, we simulated Illumina paired-end reads from four reference genomes and a lentiviral vector. For the reference genomes, the HXB2 non-circulating strain was chosen along with three contemporary circulating isolates from our 144 LANL database of strains, accessions: MK114636, OM203601, and ON816670). The circulating strains were selected randomly using Microsoft Excel’s RANDBETWEEN() function. For the lentiviral vector, pLVX.TRE3G.eGFP (accession: MH325104.1; see below) was used.

Simulated reads were generated using randomreads.sh from the BBMap toolkit (v39.06) with the following parameters: 500 read pairs per genome, 150 bp read length, 250—500 bp insert size range, and a mean Phred quality score of 33. This score was chosen because it was approximately the same mean quality score as trimmed wastewater reads. Read names were trimmed and batch-renamed to ensure compatibility with downstream tools. Simulated reads were then randomly down sampled in read length between 30 and 150 bps to reflect real-world trimming effects. Simulated reads were then treated as single-end reads and processed identically to environmental samples, including competitive mapping, read classification, and phylogenetic placement. These in silico controls confirm the ability of our pipeline to accurately preserve source-genome identity and distinguish circulating from non-circulating HIV-1 (Supplemental Figs. 2 and 3).

### Read mapping visualization and comparative genomic alignment

Read-level alignment plots and genome coverage profiles were generated using a custom Python script that integrates mapped read positions, reference genome annotations, and per-base coverage data. BAM files were visualized alongside reference sequences to display genomic features such as genes, regulatory regions, and primer binding sites.

The script produced three vertically stacked panels: (1) genome-wide read coverage, (2) annotated features with strand-specific directional arrows and labels, and (3) stacked read blocks showing individual alignments. Coverage was computed directly from BAM alignments using pysam (v0.22.1), while feature annotations were parsed using Biopython (v1.85) to preserve compound locations and relevant qualifiers. Final figures were rendered with matplotlib (v3.9.1) and saved as high-resolution vector images. These visualizations were used to identify regional hotspots of read enrichment across the HIV-1 genome.

To examine the sequence-level similarity between reference HIV-1 isolates and engineered lentiviral vectors, we developed a second visualization script that overlay MAUVE progressive genome alignments. This module parses XMFA-formatted alignments created in Mauve (v2015-02-26) using the progressiveMauve algorithm and displays local colinear blocks (LCBs) between HXB2 and the lentiviral vector pLVX.TRE3G.eGFP (accession: MH325104.1). pLVX.TRE3G.eGFP was selected as a representative lentiviral vector because the consensus sequence from high-coverage HXB2-mapping regions aligned strongly with it via megaBLAST. Blocks are color-coded, and nucleotide identity is shown using a smoothed sliding window. Annotated gene features from both genomes are aligned in parallel with strand-aware arrows, enabling comparison of coding structure and identity. These comparative plots highlight conserved regions that may confound source attribution in environmental reads. All visual elements were generated using Biopython, pysam, and matplotlib.

### Spatiotemporal analysis of HIV-1 reads in wastewater

To assess spatiotemporal trends, we grouped HIV-1 reads by sample site, city, and collection date. All reads aligning to HIV-1—regardless of classification—were included in aggregate analyses of total signal. For lineage-specific analysis, we restricted reads classified as circulating and required a circulating-to-non-circulating (C/N) ratio greater than 1 for inclusion. Reads classified as “shared” were excluded from lineage-specific plots.

Read counts were obtained from summary files generated during mapped read analysis and totaled per site and city. Temporal aggregation was performed on a monthly basis, where applicable. All figures were generated as bar graphs and stacked bar graphs (Figs. 2 and 7) using R (v4.3.0) tidyverse, cowplot (v1.2.0), grid (v3.6.2), gridExtra (v2.3), stringr (v1.6.0), dplyr (v1.1.2), and RcmdrMisc (v.2.10.1) packages.

To visualize HIV-1 detection across all collection dates and geographic areas with site-level resolution, we generated a dot plot where each point represents a weekly sample from a specific site. Points were colored and sized based on detection status and number of reads detected, respectively. Read counts were log_10_-transformed and used to scale marker size continuously, enabling intuitive visual comparison of viral load intensity over time. Sites were ordered alphanumerically, and non-detects were indicated with a gray ‘x’. This plot was produced using a custom Python script with the matplotlib (v3.9.1), seaborn (v0.13.2), and pandas (v2.3.0) libraries (Fig. 1).

### Analysis of the origin of reads mapping to circulating HIV isolates

To characterize the geographic and genomic distribution of reads classified as circulating (C/N ratio > 1), we used read-level summary files generated during the Analysis of Mapped Reads step described above. Circulating reads were grouped by the wastewater sampling site and city from which they originated, enabling spatial attribution. Aggregated read counts were visualized in R (v4.3.0) using the ggplot2 package, with final formatting performed in Inkscape (v1.4).

To evaluate the genomic distribution of circulating reads, we aligned them to the HXB2 reference genome using Geneious Assembler (v2025.0.3) and plotted genome-wide coverage using the custom Python script described in the *Read Mapping Visualization and Comparative Genomic Alignment* subsection above. Based on this alignment, eight distinct genomic regions were selected for further analysis based on visually apparent coverage enrichment. For each region, all circulating reads mapping within the specified coordinates were extracted, and their associated sampling site and city were annotated using metadata linked via FASTQ headers.

This data was tabulated by genomic region and location and plotted as stacked bar graphs in R (v4.3.0) to compare how different wastewater sites contributed to the signal across the HIV-1 genome. This analysis directly informed the spatial and genomic visualization of circulating HIV-1 reads shown in Figs. 5B, C, and 6.

### Correlation of wastewater signal with HIV clinical prevalence data

HIV prevalence data at ZIP code resolution were obtained from AIDSVu (https://aidsvu.org)^8^, a publicly accessible database maintained by Emory University’s Rollins School of Public Health that disseminates data from the CDC’s National HIV Surveillance System. ZIP code-level prevalence estimates represent year-end 2023 point prevalence of persons living with diagnosed HIV per 100,000 population, as reported through the CDC’s National HIV Surveillance System. These data capture individuals with a documented HIV diagnosis alive at the end of 2023 and do not include estimates of undiagnosed infections or distinguish by antiretroviral treatment status or viral suppression. Data for Cities A, B, D, and H were assessed and extracted in January 2026.

To improve the geographic precision of prevalence estimates, we obtained sewershed catchment boundaries from available utility-reported and modeled datasets, including the EPA National Sewershed Dataset for selected facilities. Catchment polygons were spatially intersected with U.S. Census Bureau Zip Code Tabulation Area (ZCTA shapefiles; 2023 vintage) using GeoPandas (v1.1.3). For each sewershed, a population-weighted mean HIV prevalence was computed by weighting each overlapping ZCTA’s AIDSVu prevalence estimate by the fraction of its population falling within the catchment boundary, estimated via areal interpolation assuming uniform population distribution within ZCTA.

Population-weighted prevalence for sewershed S:

4 $${Prev}\left(S\right)=\frac{{\sum }_{i}{Prev}\left({Z}_{i}\right)*{Pop}({Z}_{i}\bigcap S)}{{\sum }_{i}{Pop}({Z}_{i}\bigcap S)}$$

Estimated population of ZCTA I within sewershed S, via areal interpolation:

5 $${Pop}\left({Z}_{i}\bigcap S\right)={Pop}\left({Z}_{i}\right)*\frac{{Area}\left({Z}_{i}\bigcap S\right)}{{Area}\left({Z}_{i}\right)}$$

Where:

- $${Prev}\left(S\right)={estimated\; HIV\; prevalence\; for\; sewershed\; S}$$
- $${Prev}\left({Z}_{i}\right)={AIDSVu}-{reported\; diagnosed\; HIV\; prevalence\; for\; ZCTA\; i}$$
- $${Pop}\left({Z}_{i}\right)={total\; population\; of\; ZCTA\; i}$$
- $${Pop}({Z}_{i}\bigcap S)={estimated\; population\; of\; the\; portion\; of\; ZCTA\; i\; that\; falls\; within\; sewershed\; S}$$
- $${Area}\left({Z}_{i}\bigcap S\right)={geographic\; area\; of\; the\; intersection\; between\; ZCTA\; i\; and\; sewershed\; s}$$
- $${Area}\left({Z}_{i}\right)={total\; geographic\; area\; of\; ZCTA\; i}$$
- $${\sum }_{i}={summation\; over\; all\; ZCTAs\; that\; intersect\; sewershed\; S}$$

We acknowledge that the uniform population assumption is an approximation, particularly in heterogeneous urban areas. AIDSVu prevalence inputs were restricted to 2023 ZIP-code records with ZIP Code Rate Stability = “Y”. ZIP code population was estimated from AIDSVu-reported ZIP Code Cases and Zip Code Rate as implied by population = (cases x 100,000) / rate.

To maintain site anonymity, prevalence values were converted to z-scores (z = (x - µ) / σ), where μ and σ represent the mean and standard deviation of the prevalence estimates across the sites included in each respective analysis panel. This standardization preserves proportional relationships for correlation analysis without exposing rates that could identify specific locations.

Wastewater read counts were standardized by the number of samples collected per site and reported as mean reads per sample to account for variable sampling frequency. For two sampling points within the same city that fed into the same wastewater treatment plant, reads were aggregated, and the total number of sampling events (not individual inlet collections) was used as the denominator. One site in City A serving a major transportation hub was excluded due to high transient:resident population ratios that violate the assumption that the wastewater signal reflects local community burden.

Pearson correlation coefficients with two-tailed *p*-values and 95% confidence intervals were calculated using Python scipy (v. 1.16.3; Fisher z-transformation for confidence intervals) to assess associations between z-scored HIV prevalence and wastewater read counts. To evaluate the influence of individual high-prevalence sites on the observed correlation, we computed Cook’s distance for all observations in the primary regression using ordinary least squares (OLS) implemented in statsmodels. Sites exceeding the conventional thresholds were reported, and sensitivity analyses excluding individual influential sites were performed to assess robustness (Supplemental Fig. 5).

### BLAST-Based classification of HIV-like reads to synthetic or HIV origins

To independently assess the origin of HIV-1-like sequences and validate potential vector-derived signal, we performed nucleotide BLAST (blastn) on all reads. These reads were queried against a custom BLAST database combining the NCBI nucleotide (nt) core dataset and the NCBI patent sequence collection.

Reads were classified into four categories based on their top-scoring BLAST hits: (1) HIV-only, where the top hit matched exclusively to sequences assigned to the HIV-1 taxonomic ID (taxid: 11676); (2) synthetic-only, where the top hit(s) matched exclusively to patented or synthetic vector-associated sequences; (3) ambiguous, where equivalent matches were found to both HIV and synthetic (or other) sequences, preventing unambiguous classification; and (4) unclassified, where no high-confidence match was returned.

This BLAST-based classification scheme was used to detect putative vector-derived elements, support identification of chimeric reads, and refine attribution of HIV signal in wastewater. It served as a complementary approach to competitive alignment-based C/N ratio classification and helped identify persistent synthetic vector contributions.

### Ethics statement

This study was determined to be exempt from human subjects review by the Baylor College of Medicine Institutional Review Board. Wastewater samples are environmental specimens collected from municipal infrastructure and cannot be traced to identifiable individuals. No human participants were recruited, and no individual-level human data were analyzed. Human-derived sequencing reads were removed during bioinformatics quality control and were not included in any analyses. City and site identities were anonymized throughout the study to protect community privacy.

### Reporting summary

Further information on research design is available in the Nature Portfolio Reporting Summary linked to this article.

## Supplementary information

Supplementary Information

Reporting Summary

Transparent Peer Review file

## Source data

Source Data

## Data Availability

Raw sequencing reads are available under NCBI BioProject accession number PRJNA966185. Aggregated read classification summaries used to generate figures and analyses in this study are available at: https://github.com/TAILOR-Lab/hiv-wastewater-study (archived at 10.5281/zenodo.19561428). Source data are provided with this paper. HIV prevalence data used in correlation analyses are publicly available through AIDSVu (https://aidsvu.org). Site-level metadata linking sampling locations to city identities are restricted to protect community privacy and minimize potential stigma associated with geographic HIV detection. Access to restricted metadata is limited to researchers conducting public health or epidemiological research who agree not to publicly disclose location identifiers. To meet our commitment to confidentiality, any data request will be evaluated in consultation with local public health authorities. Requests may be directed to the corresponding author (A.W.M., maresso@bcm.edu) and may take up to 60 days. De-identified data may be provided; release of location-identifying metadata is not guaranteed. Approved data will remain available for the duration of the requesting study. Source data are provided in this paper.

## Code availability

All core code used in this study, including the TREx abundance extraction tool (https://github.com/TAILOR-Lab/TREx) and the EsViritu viral mapping pipeline (https://github.com/cmmr/EsViritu), is publicly available. Custom scripts for read classification, competitive mapping analysis, prevalence correlation, coverage visualization, and figure generation are archived at Zenodo (10.5281/zenodo.19561428)^55^ and available via GitHub: https://github.com/TAILOR-Lab/hiv-wastewater-study.
