# Supplementary material for: Statewide multi-year wastewater sequencing reveals dual origins of HIV-1 signal
Source: Nat Commun. 2026 Jun 11;17:7428. doi: 10.1038/s41467-026-74140-7 (PMC13408598; doi:10.1038/s41467-026-74140-7)
Supplement: Supplementary file 1 — Supplementary Information [file 41467_2026_74140_MOESM1_ESM.pdf]

Supplemental Information

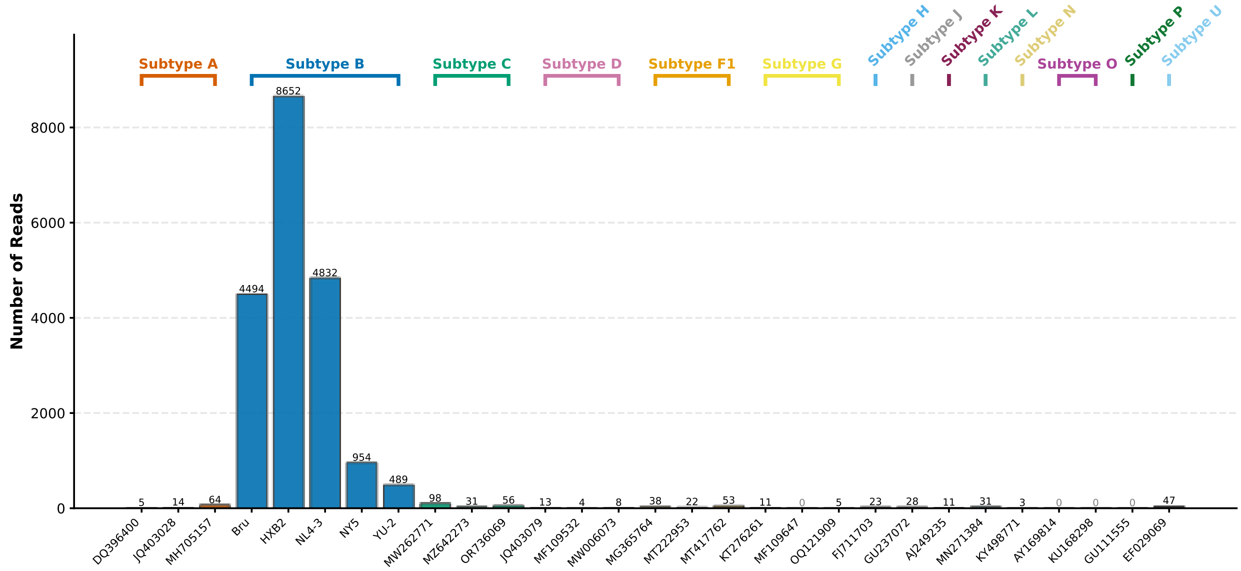

Supplemental Figure 1. HIV-1 reads sampled in wastewater belong almost exclusively to Subtype B HIV-1. Number of reads mapping to 14 known HIV-1 subtypes, (3 isolates for A, C, D, F1, G; 2 isolates for O, and 1 isolate for H, J, K, L, N, P, and U subtypes) alongside the 5 non-circulating B subtype isolates. Reference subtype ID or accession ID is present on the x-axis, with raw read count mapping to each reference on the y-axis. Source data are provided as a Source Data file.

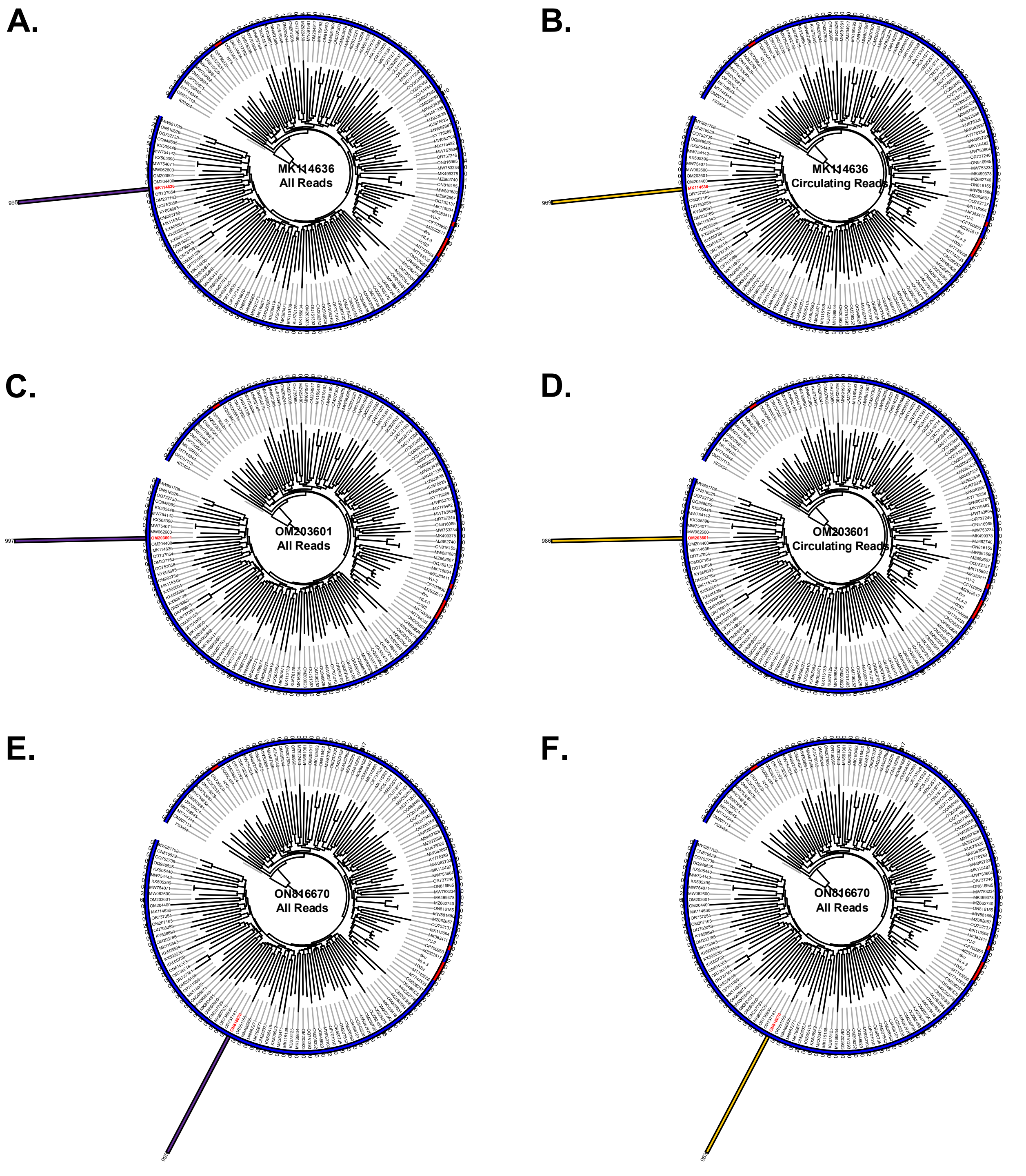

Supplemental Figure 2. ***In Silico*** **Validation Using Circulating HIV-1 Isolates.** Placement of Simulated reads generated from three circulating HIV-1 isolates (MK114636, OM203601, and ON816670). Simulated reads were generated *in silico* and processed identically to wastewater-derived reads using the same competitive mapping and classification pipeline. Each panel shows the number of reads aligning to circulating reference genomes across a phylogeny of 144 North American subtype B isolates (blue) and 5 non-circulating reference strains (red). (**A**, **C**, **E**)

All simulated reads from each genome. (**B**, **D**, **F**) Subset of reads classified as “circulating” by the circulating/non-circulating (C/N) identity ratio heuristic. Source genomes are colored red. Reads consistently cluster around their source genomes, validating the specificity of the C/N ratio-based classification strategy. Source data are provided as a Source Data file.

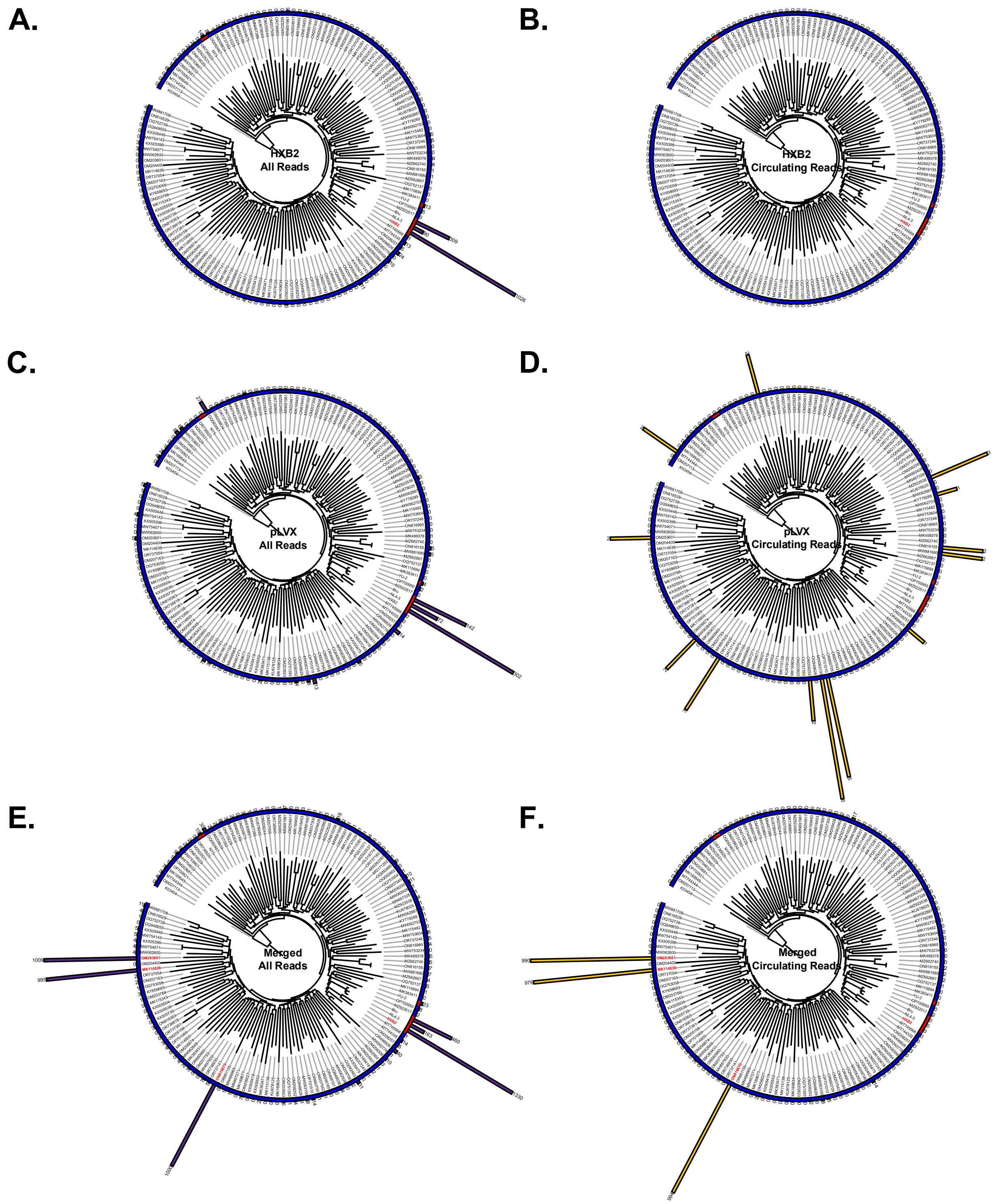

Supplemental Figure 3. ***In Silico*** **Evaluation of Non-Circulating and Vector-Associated HIV-1 Reads.** Phylogenetic placement of simulated reads from a non-circulating strain (HXB2; **A**, **B**), a lentiviral vector backbone (pLVX.TRE3G.eGFP; **C**, **D**), and a merged control pool including HXB2, the lentiviral vector, and the three circulating isolates in Supplemental Figure 2

(**E**, **F**). (**A**, **C**, **E**) All simulated reads from each source. (**B**, **D**, **F**) Reads classified as “circulating” based on the C/N identity ratio heuristic. Source genomes are colored red. Results highlight the ability of the framework to correctly exclude vector- and non-circulating reference clone-derived reads from circulating classification, even when co-present in a mixed pool. Source data are provided as a Source Data file.

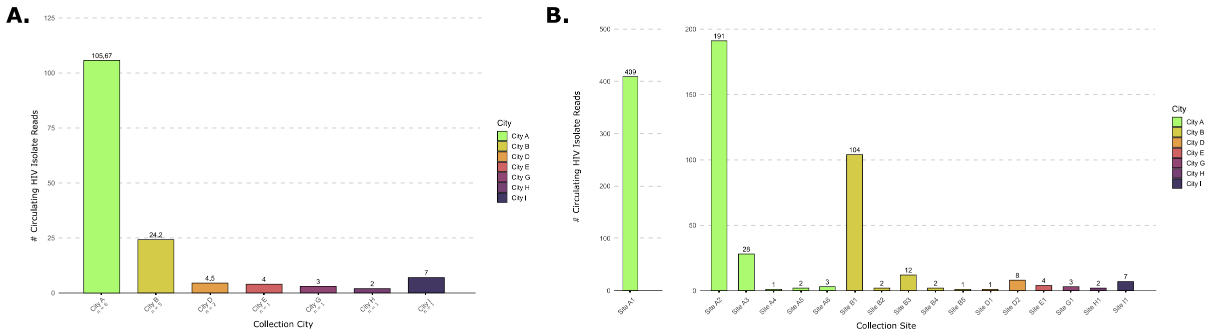

Supplemental Figure 4. Geographic Distribution of Wastewater Reads aligned to Circulating HIV-1 Isolates. (**A**) Mean number of circulating reads per site, aggregated by city, where n represents the number of sites within each city. This normalization highlights city-to-city variability in detection independent of sampling density. (**B**) Site-level read counts for each wastewater collection point, color-coded by city. Site A1 and A2 (City A) exhibit the highest read counts, consistent with localized enrichment of circulating HIV-1 signal. Source data are provided as a Source Data file.

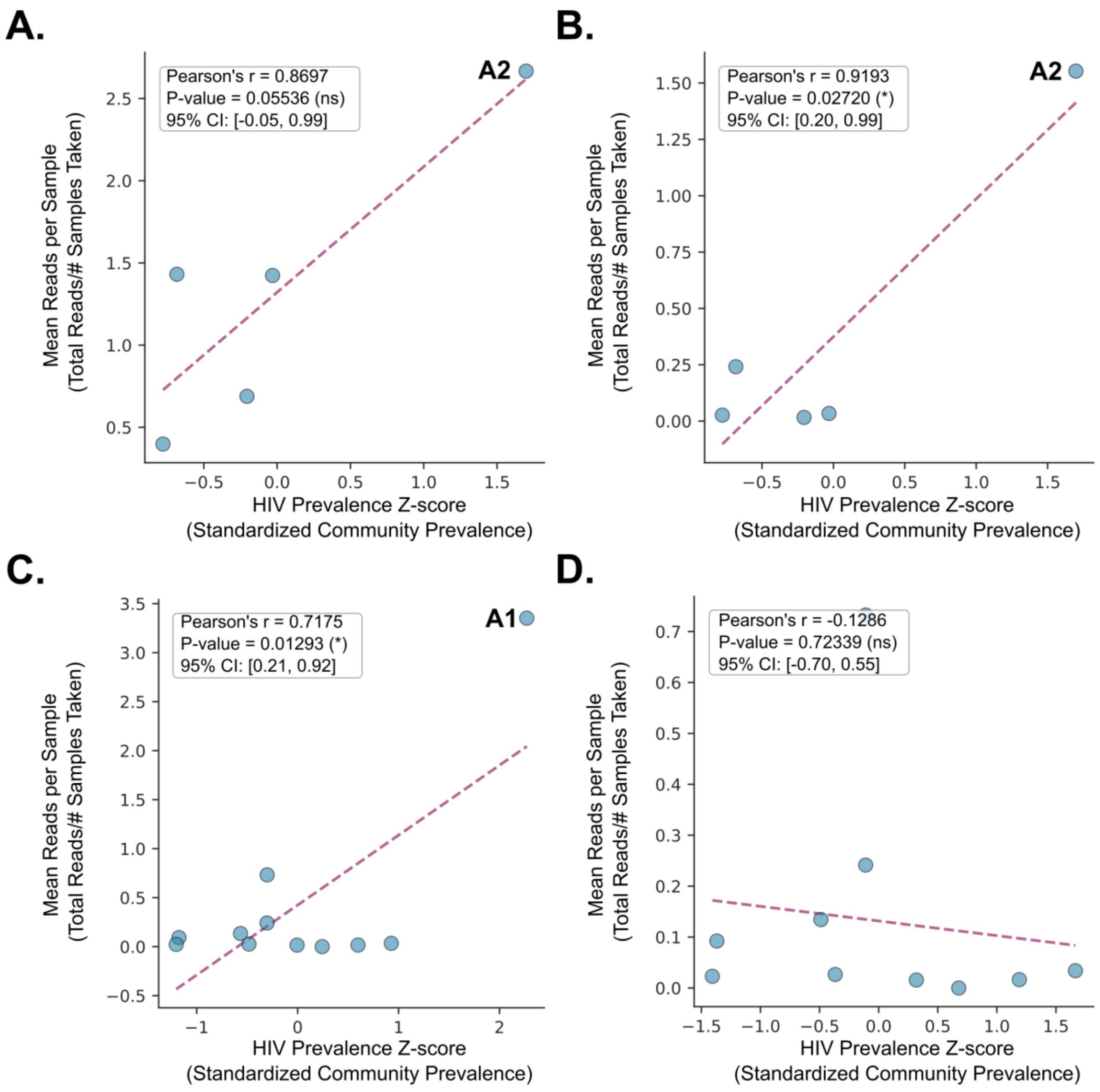

Supplemental Figure 5. Sensitivity Analysis for the Correlation Between Wastewater HIV-1 Circulating Reads and Diagnosed HIV Prevalence. Wastewater read counts (mean reads per sample) were compared with year-end 2023 point prevalence of diagnosed HIV per 100,000 population (AIDSVu/CDC). Prevalence values are expressed as z-scores calculated across the sites included in each panel to preserve anonymity. (**A**) All reads versus prevalence for City A (n=4 sampling sites) and City H (n=1 sampling site; within the metropolitan area of City A) sites only (total n=5 sampling sites; Pearson's r = 0.87, two-tailed p = 0.055, 95% CI [-0.05, 0.99]). (**B**) Circulating reads versus prevalence for City A and City H sites only (n=5 sampling sites; r = 0.92, two-tailed p = 0.027, 95% CI [0.20, 0.99]). (**C**) Circulating reads versus prevalence across all cities with Site A2 excluded (n=11 sampling sites; r = 0.72, two-tailed p = 0.013, 95% CI [0.21, 0.92]). This

panel provides a direct robustness check against the influence of the high-prevalence site. (**D**) Circulating reads versus prevalence across all cities with both Sites A1 and A2 excluded ($n=10$ sampling sites; $r = -0.13$, two-tailed $p = 0.72$, 95% CI [-0.70, 0.55]). The non-significant result is consistent with range restriction; with the two highest-prevalence catchments removed, insufficient dynamic range remains to detect a correlation statistically. Dashed lines represent linear regression fits. ns $p > 0.05$; * $p < 0.05$; ** $p < 0.01$. Source data are provided as a Source Data file.

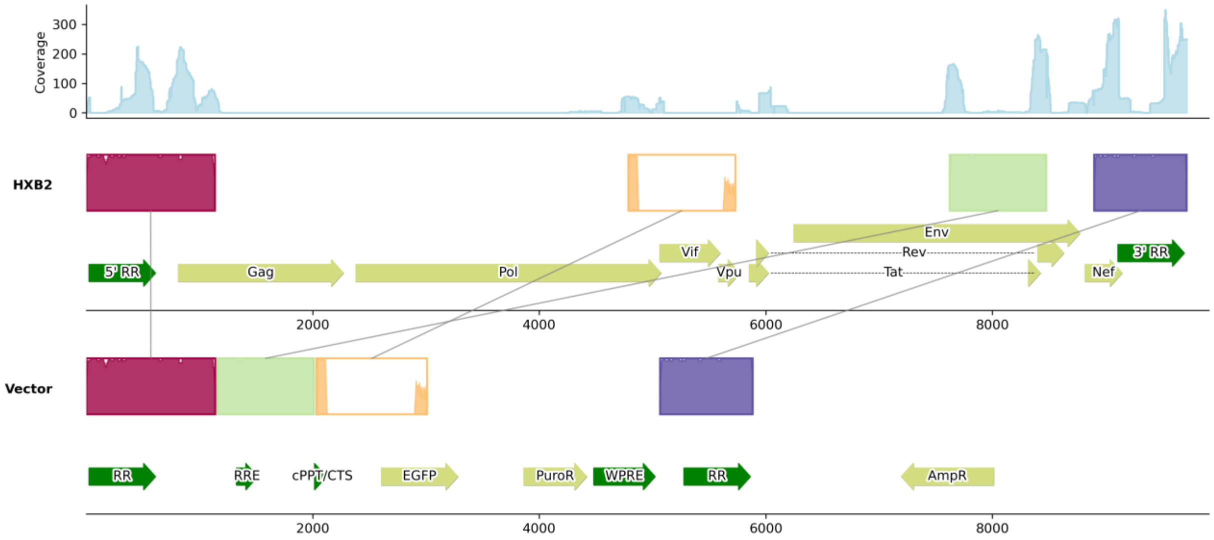

Supplemental Figure 6. Coverage of Synthetic Reads Along Lentiviral Vector Backbones. Coverage plot displaying wastewater reads classified as synthetic (aligning exclusively to synthetic or patented sequences via BLAST) mapped to the non-circulating HXB2 genome (top), aligned with the representative lentiviral vector pLVX.TRE3G.eGFP (accession: MH325104.1; bottom). Coverage depth (y-axis) is shown across the HXB2 genome (x-axis), with major HIV-1 genes annotated. Colored blocks represent locally collinear blocks from a progressive Mauve genome alignment, indicating regions of sequence homology between HXB2 and the lentiviral vector; matching colors denote corresponding homologous segments across the two genomes. Connecting gray lines link homologous regions between the HXB2 and vector. The vector (bottom) is annotated with key structural and regulatory elements: long terminal repeat regions (RR), Rev Response Element (RRE), central polypurine tract/central termination sequence (cPPT/CTS), Enhanced Green Fluorescent Protein (EGFP), puromycin resistance gene (PuroR), Woodchuck Hepatitis Virus Posttranscriptional Regulatory Element (WPRE), and ampicillin resistance gene (AmpR). Coverage peaks on HXB2 correspond to regions sharing homology with elements retained in the vector backbone, supporting the vector origin of these sequences. Source data are provided as a Source Data file.
