# Supplementary material for: Statewide multi-year wastewater sequencing reveals dual origins of HIV-1 signal
Source: Nat Commun. 2026 Jun 11;17:7428. doi: 10.1038/s41467-026-74140-7 (PMC13408598; doi:10.1038/s41467-026-74140-7)
Supplement: Supplementary file 2 — Reporting Summary [file 41467_2026_74140_MOESM2_ESM.pdf]

nature portfolio

Corresponding author(s): Anthony W. Maresso, Thomas P. Giordano

Last updated by author(s): May 7, 2026

Reporting Summary

Nature Portfolio wishes to improve the reproducibility of the work that we publish. This form provides structure for consistency and transparency in reporting. For further information on Nature Portfolio policies, see our Editorial Policies and the Editorial Policy Checklist.

Statistics

For all statistical analyses, confirm that the following items are present in the figure legend, table legend, main text, or Methods section.

n/a	Confirmed	
☐	☒	The exact sample size (*n*) for each experimental group/condition, given as a discrete number and unit of measurement
☐	☒	A statement on whether measurements were taken from distinct samples or whether the same sample was measured repeatedly
☐	☒	The statistical test(s) used AND whether they are one- or two-sided<br>*Only common tests should be described solely by name; describe more complex techniques in the Methods section.*
☐	☒	A description of all covariates tested
☐	☒	A description of any assumptions or corrections, such as tests of normality and adjustment for multiple comparisons
☐	☒	A full description of the statistical parameters including central tendency (e.g. means) or other basic estimates (e.g. regression coefficient) AND variation (e.g. standard deviation) or associated estimates of uncertainty (e.g. confidence intervals)
☐	☒	For null hypothesis testing, the test statistic (e.g. *F*, *t*, *r*) with confidence intervals, effect sizes, degrees of freedom and *P* value noted<br>*Give P values as exact values whenever suitable.*
☒	☐	For Bayesian analysis, information on the choice of priors and Markov chain Monte Carlo settings
☒	☐	For hierarchical and complex designs, identification of the appropriate level for tests and full reporting of outcomes
☐	☒	Estimates of effect sizes (e.g. Cohen's *d*, Pearson's *r*), indicating how they were calculated

Our web collection on statistics for biologists contains articles on many of the points above.

Software and code

Policy information about availability of computer code

Data collection	HIV prevalence data at ZIP code resolution was obtained from AIDSVu (https://aidsvu.org). To improve the geographic precision of prevalence estimates, we obtained sewershed catchments boundaries from available utility-reported and modeled datasets, including the EPA National Sewershed Dataset for selected facilities. Catchment polygons were spatially intersected with U.S. Census Bureau Zip Code Tabulation Area (ZCTA shapefiles; 2023 vintage) using GeoPandas.

Data analysis	Software tools used in this study include both open-source analysis software as well as custom analysis code developed by the authors of the study. Publicly available open-source tools include EsViritu (v0.2.3) for initial read classification, TREx (v0.1) for read abundance estimation, Python (3.12.7) and R (v4.3.0) for data processing and visualization respectively. In addition, FADE (v0.6.0) was used to identify and excise chimeric reads containing inverted terminal repeats.<br>BBDuk (v38.84) was used to carry out trimming of low-quality reads and minimap2 (v2.26) was used to align reads to reference HIV genomes. Geneious Prime and Geneious Assembler (v2025.0.3) were used to map HIV reads to reference HIV genomes, and phylogenetic analyses were carried out with MAFFT (v7.453), IQ-TREE (v2.1.4) and iTOL (7.5.1). Genomic coverage maps were made with the matplotlib (v3.9.1) python package after calucating coverage with pysam (v0.22.1) and Biopython (v1.85).<br>Genome alignments between HIV references and lentiviral vectors were developed with custom scripts using Mauve (v2015-02-2026). Figures representing spatio-temporal analysis of HIV reads was carried out in R (v4.3.0) tidyverse, cowplot (v1.2.0), grid (v3.6.2), gridExtra (v2.3), stringr (v1.6.0), dplyr (v1.1.2), and RcmdrMisc (v.2.10.1) packages. In addition, plots were generated with custom Python scripts utilizing packages including matplotlib, seaborn (v0.13.2) and pandas (v2.3.0). Final touches to figures were added using Inkscape (v1.4).<br>NCBI BLAST was carried out on HIV reads to assess the origin of HIV-1-like sequences and validate potential vector-derived signal.<br>Finally, all custom scripts developed to analyze data has been included in a dedicated github repository and can be accessed at https://github.com/TAILOR-Lab/hiv-wastewater-study.

For manuscripts utilizing custom algorithms or software that are central to the research but not yet described in published literature, software must be made available to editors and reviewers. We strongly encourage code deposition in a community repository (e.g. GitHub). See the Nature Portfolio guidelines for submitting code & software for further information.

Data

Policy information about availability of data

All manuscripts must include a data availability statement. This statement should provide the following information, where applicable:

- Accession codes, unique identifiers, or web links for publicly available datasets
- A description of any restrictions on data availability
- For clinical datasets or third party data, please ensure that the statement adheres to our policy

Raw sequencing reads are available under NCBI BioProject accession number PRJNA966185 (https://www.ncbi.nlm.nih.gov/bioproject/PRJNA966185). Aggregated read classification summaries used to generate figures and analyses in this study are available at: https://github.com/TAILOR-Lab/hiv-wastewater-study (archived at https://doi.org/10.5281/zenodo.19561428). Source data are provided with this paper. HIV prevalence data used in correlation analyses are publicly available through AIDSVu (https://aidsvu.org). Site-level metadata linking sampling locations to city identities are restricted to protect community privacy and minimize potential stigma associated with geographic HIV detection. Access to restricted metadata is limited to researchers conducting public health or epidemiological research who agree not to publicly disclose location identifiers. To meet our commitment to confidentiality, any data request will be evaluated in consultation with local public health authorities. Requests may be directed to the corresponding author (A.W.M., maresso@bcm.edu) and may take up to 60 days. De-identified data may be provided; release of location-identifying metadata is not guaranteed. Approved data will remain available for the duration of the requesting study.

Research involving human participants, their data, or biological material

Policy information about studies with human participants or human data. See also policy information about sex, gender (identity/presentation), and sexual orientation and race, ethnicity and racism.

Reporting on sex and gender	This study analyzes pooled municipal wastewater samples. No individual-level human data were collected and no human participants were involved.
Reporting on race, ethnicity, or other socially relevant groupings	This study does not involve human participants or individual-level demographic data. Community-level HIV prevalence estimates from AIDSVu were used for ecological correlation only.
Population characteristics	Wastewater samples represent aggregate inputs from municipal sewersheds. No individual-level characteristics were assessed.
Recruitment	No human participants were recruited. Samples consist of municipal wastewater collected from treatment plant infrastructure.
Ethics oversight	This study was determined to be exempt from IRB review by the Baylor College of Medicine Institutional Review Board. The study analyzes environmental wastewater samples collected from municipal infrastructure. No human participants were recruited, no biological specimens were collected from individuals, and human-derived sequencing reads were removed during bioinformatics quality control and not analyzed.

Note that full information on the approval of the study protocol must also be provided in the manuscript.

Field-specific reporting

Please select the one below that is the best fit for your research. If you are not sure, read the appropriate sections before making your selection.

☐ Life sciences ☐ Behavioural & social sciences ☒ Ecological, evolutionary & environmental sciences

For a reference copy of the document with all sections, see nature.com/documents/nr-reporting-summary-flat.pdf

Ecological, evolutionary & environmental sciences study design

All studies must disclose on these points even when the disclosure is negative.

Study description	Observational environmental surveillance study using hybrid-capture metagenomic sequencing to detect and classify HIV-1 signal in municipal wastewater. Wastewater samples (n=2,086) were collected from 40 catchment sites across 15 Texas cities over approximately 3 years (May 2022 to December 2024). The study uses a nested design: samples are nested within sites, which are nested within cities. No experimental treatments or manipulations were applied. The primary analytical framework classifies HIV-1 reads as circulating (community-derived), non-circulating (vector-derived), or shared (conserved regions) based on competitive alignment to a curated database of 149 HIV-1 subtype B reference genomes.
Research sample	Untreated municipal wastewater influent collected from 40 catchment sites serving 15 anonymized Texas cities (Cities A through I, Sites 1 through 7). Sites were selected as part of the Texas Wastewater and Environmental Biomonitoring (TexWEB) program to capture geographically and demographically diverse urban sewersheds. Samples represent pooled community-level biological inputs to wastewater treatment infrastructure and are not traceable to individual persons. For the correlation analysis, community-level HIV prevalence data (year-end 2023 diagnosed prevalence per 100,000 population) were obtained from AIDSVu, a publicly available database maintained by Emory University that disseminates CDC National HIV Surveillance System data. Prevalence data were available at ZIP code resolution for Cities A, B, D, and H (n=12 sampling sites across these cities).
Sampling strategy	Wastewater samples were collected on a weekly to monthly basis depending on site capacity and operational logistics within the TexWEB program. No formal power analysis or sample-size calculation was performed, as this is an observational surveillance study. Sample sizes were determined by the existing TexWEB sampling infrastructure and operational capacity at each treatment facility. The total of 2,086 samples across 40 sites over approximately 3 years provides broad temporal and geographic coverage. Individual site sampling frequency ranged from 1 to 142 samples over the study period. For the prevalence correlation analysis, sample sizes (n=10 to 12 sites depending on the sensitivity analysis) were constrained by the availability of ZIP code-level HIV prevalence data from AIDSVu for the anonymized cities.
Data collection	24-hour composite wastewater samples were collected at municipal wastewater treatment plants using automated composite samplers operated by local utility staff. Sample bottles were surface-decontaminated with 10% bleach, sealed in sterile bags, and shipped on ice to Baylor College of Medicine, where they were stored at 4 degrees C and processed within 24 hours. Nucleic acid extraction, reverse transcription, library preparation (Twist Library Preparation EF 2.0 Kit), and hybrid-capture enrichment (Twist Comprehensive Viral Research Panel) were performed by laboratory personnel at Baylor College of Medicine. Sequencing was performed on Illumina platforms. Bioinformatic classification was conducted using the EsViritu pipeline (v0.2.3) followed by custom competitive mapping and read classification scripts. Sample metadata (site, city, collection date) were recorded by TexWEB program staff using standardized collection logs and linked to sequencing data via dual-index barcodes.
Timing and spatial scale	Data collection began in May 2022 and continued through December 2024 (approximately 3 years). Sampling frequency was weekly to monthly depending on site, with no systematic gaps in collection. The spatial scale encompasses 40 wastewater catchment sites across 15 cities distributed throughout the state of Texas. Each catchment site corresponds to a defined sewershed serving a distinct geographic area within its city. For the correlation analysis, sewershed catchment boundaries were spatially intersected with U.S. Census Bureau ZIP Code Tabulation Area (ZCTA) polygons to compute population-weighted HIV prevalence estimates at the catchment level. AIDSVu prevalence data reflect year-end 2023 diagnoses and were extracted in January 2026.
Data exclusions	EsViritu-classified HIV-1 reads that did not map to any genome in the curated 149-genome reference set were excluded from circulating versus non-circulating classification (5,973 of 16,710 reads, 36%). This exclusion criterion was pre-established based on the requirement for competitive alignment to enable classification. These unmapped reads were primarily short fragments with insufficient sequence for reliable competitive mapping. Sequencing artifacts containing inverted terminal repeats were identified and hard-clipped using the FADE tool (v0.6.0) as a pre-established quality control step. One wastewater site in City A serving a major transportation hub was excluded from the prevalence correlation analysis because transient populations violated the assumption that wastewater signal reflects local community burden; this exclusion criterion was pre-established based on known catchment characteristics.
Reproducibility	In silico validation was performed by simulating Illumina paired-end reads from three circulating HIV-1 isolates, one non-circulating reference (HXB2), and a lentiviral vector (pLVX.TRE3G.eGFP). Simulated reads were processed identically to environmental samples and consistently mapped back to their source genomes, confirming the specificity of the classification pipeline (Supplemental Figures 2 and 3). Multiple sensitivity analyses were performed for the prevalence correlation, including systematic exclusion of individual high-influence sites (Cook's distance analysis) and range restriction testing. The correlation between circulating HIV-1 reads and community prevalence was robust across multiple scenarios ($r = 0.68$ to 0.92, all $p < 0.05$ when adequate dynamic range in prevalence was retained).
Randomization	Randomization is not relevant to this observational environmental surveillance study. Wastewater sampling sites were selected based on geographic coverage and operational availability within the TexWEB program, not through random allocation. For the in silico validation, circulating reference strains used for read simulation were selected randomly from the 144-genome LANL database using a random number generator (Microsoft Excel RANDBETWEEN function).
Blinding	Blinding was not relevant to this study. Wastewater sample processing and sequencing were performed without reference to site-level HIV prevalence data, which were obtained independently from AIDSVu after sequencing and bioinformatic classification were complete. City and site identities were anonymized (Cities A through I, Sites 1 through 7) throughout analysis and reporting to protect community privacy.

Did the study involve field work? ☐ Yes ☒ No

Reporting for specific materials, systems and methods

We require information from authors about some types of materials, experimental systems and methods used in many studies. Here, indicate whether each material, system or method listed is relevant to your study. If you are not sure if a list item applies to your research, read the appropriate section before selecting a response.

Materials & experimental systems

n/a	Involved in the study
[x]	[ ] Antibodies
[x]	[ ] Eukaryotic cell lines
[x]	[ ] Palaeontology and archaeology
[x]	[ ] Animals and other organisms
[x]	[ ] Clinical data
[x]	[ ] Dual use research of concern
[x]	[ ] Plants

Methods

n/a	Involved in the study
[x]	[ ] ChIP-seq
[x]	[ ] Flow cytometry
[x]	[ ] MRI-based neuroimaging

Plants

Seed stocks	This study does not involve plant material, seed stocks, or botanical specimens.
Novel plant genotypes	No plant genotypes were produced or used in this study.
Authentication	No plant material was used; no authentication procedures were required.
