# Supplementary material for: Statewide multi-year wastewater sequencing reveals dual origins of HIV-1 signal
Source: Nat Commun. 2026 Jun 11;17:7428. doi: 10.1038/s41467-026-74140-7 (PMC13408598; doi:10.1038/s41467-026-74140-7)
Supplement: Supplementary file 3 — Transparent Peer Review file [file 41467_2026_74140_MOESM3_ESM.pdf]

nature portfolio

Peer Review File

Statewide Multi-Year Wastewater Sequencing Reveals Dual Origins of HIV-1 Signal

Corresponding Author: Dr Anthony Maresso

This file contains all reviewer reports in order by version, followed by all author rebuttals in order by version.

Version 0:

Reviewer comments:

Reviewer #1

(Remarks to the Author)
The authors have conducted a wastewater-based epidemiology study of HIV, using genetic sequencing. They have found a large degree of an HIV signal is suspected to be driven by gene therapy or medical research. Whatever the source the vast majority of HIV recovered in wastewater was unrelated to HIV known to be circulating in the population. The results are quite interesting and have some important conclusions for the potential to use wastewater surveillance for HIV. I have a number of questions and suggestions.
1) In the introduction the authors frame the study as a complement to clinical HIV surveillance, with the ability to identify areas where HIV is circulating and maybe missed in diagnosis. The authors make inferences based upon the genetic makeup of the HIV recovered from wastewater, but they never show the clinical data of prevalence or estimated incidence of HIV between the sampling sites. Could the authors include some of that information, while maintaining the anonymity of the sites? This would go a long way in helping to interpret the results, particularly figure 5B that shows the HIV signal is predominantly from City A. Including the size of the population contributing to the sampling sites would be important as well.
2) Figure 5B raises a very large question – why is region 4 of the HIV genome so dominant in City B, where all the other regions of the genome are dominant in city A? Do the authors have any theories – it's quite perplexing. And then a related question for figure 5C – why are certain regions of the genome only being identified from certain sampling points? Is this a small sample size problem? How many HIV positive samples are represented here?
3) Thinking through the sample sizes here, can the authors clearly state the number and proportion of samples that were positive for HIV? I expect this in lines 85-86, here the authors should report basic HIV positive statistics by sampling site and across time. I see it in the supplemental figures. From Figure 1B, it looks like about half the sites had 1-2 detections and half the sites had a few more – closer to 6-7? Is that accurate? It would be good to clearly state this. Also, some of the signal in figure 1B is really low – for example site B5 only had two detections, one with 2 reads and one with 1 read. Whereas site A1 had reads up to 2,399. Site A1 is so different from the rest of the sites – why is that? Its number of reads is more than all the other sites combined. Would it make sense just to focus on Site A1 – would the authors still be able to make the same inferences? The other sites are very, very different in HIV recovery. This is a big limitation in the work – the results are dominated by a single site but the analysis is presented as multiple sites. From Figure 1A it looks like two points the HIV reads were higher – could that be related to dumping in the site? Looking at supplemental figure 1, I think that belongs in the main manuscript. I recommend trimming it to remove the sites that began sampling in 2024.
4) The authors need to describe what a "read count" is and how it is used in a mixed sample context. They first use this term in line 88. The authors also need to describe their data beyond read count – what is the read length and depth of the sequencing that they obtained? From line 183 the authors state that more than 1/3 of the sequenced data could not map clearly to a reference set due to the short sequence length.
5) In the introduction, lines 62-68 the authors should cite the studies that have already examined HIV through wastewater surveillance. It looks like they've cited them in the discussion as well – would be good in both places. For example:
- Wolfe MK, Varkila MR, Zulli A, Parsonnet J, Boehm AB. Detection and quantification of human immunodeficiency virus-1 (HIV-1) total nucleic acids in wastewater settled solids from two California communities. Applied and Environmental Microbiology. 2024 Dec 18;90(12):e01477-24.
- Terwilliger A, Clark J, Avadhanula V, Feliz K, Weesner K, Murphy E, Hopkins L, Kimata JT, Giordano TP, Piedra P, Maresso A. HIV Detection in Wastewater as a Potential Epidemiological Bellwether. Available at SSRN 4257909. 2022.
- Alshehri B, Birch ON, Greaves JC. Monitoring Multiple Sexually Transmitted Pathogens Through Wastewater Surveillance. Pathogens. 2025 Jun 5;14(6):562.
6) Line 69 – revise to "Nearly all viral WBE is performed using PCR."

7) Line 440-445 in the discussion – in the 1990s wastewater was tested for infectious HIV and it was not found to be infectious. The authors could cite some of those studies such as
8) From supplemental figure 4 – does the incidence or prevalence of HIV support the disproportionate results by city here? I know the authors have stated this, but can they share any prevalence or incidence estimates while still preserving anonymity of the sites?

(Remarks on code availability)
I reviewed this as an epidemiologist.

Reviewer #2

(Remarks to the Author)
Thank you for the opportunity to review this manuscript, which evaluates a previously developed agnostic sequencing approach for wastewater surveillance to detect HIV-1. The article is generally well written and easy to follow. However, its conclusions are undermined by methodological limitations in the choice of reference database and by insufficient discussion of the ethical context. Addressing these issues, particularly by expanding sensitivity analyses and integrating a nuanced ethical discussion, would substantially improve the manuscript. I have several major considerations.
Major Comments
1. Methodological concern: reference database selection
• My major methodological concern relates to the curated reference database used to map circulating and non-circulating HIV strains. The authors employed 144 full-length subtype B HIV-1 genomes. Given the growing number of infections linked to non-B subtypes in the United States, why were these excluded?
https://pubmed.ncbi.nlm.nih.gov/30961993/
https://pubmed.ncbi.nlm.nih.gov/28318764/
The choice of reference set is critical because it fundamentally shapes read-mapping efficiency, detection sensitivity, and ultimately all downstream analyses and inferences. For example, if a locally curated dataset of HIV sequences, including non-B subtypes, were used, it is possible the authors would have captured more reads and generated more accurate results. Excluding non-B references risks systematically underestimating signals from circulating lineages.
• Relatedly, there is seemingly low coverage observed in pol, the genomic region where most U.S. sequence data are historically derived for surveillance and clinical monitoring. If pol coverage in the wastewater samples was indeed poor, did this drive the authors to avoid using local reference datasets that would have relied heavily on pol alignments? If so, this limitation should be explicitly discussed, as it may have restricted the ability to compare wastewater-derived data with the largest available source of U.S. HIV sequences.
•Since one of the manuscript's central conclusions is that lentiviral vectors may be incorrectly disposed of, a claim with substantial public health and policy implications, additional sensitivity analyses using alternative reference sets are necessary. This would allow readers to assess how robust the findings are to different assumptions.
• Furthermore, given the seriousness of this claim, were regulatory authorities informed? If not, the authors should clarify how such findings should be responsibly communicated. I also recommend tempering the tone of the discussion to reflect the uncertainty introduced by reference database limitations.
2. Ethical considerations
• The manuscript lacks sufficient discussion of the ethical issues surrounding wastewater surveillance for HIV. HIV is unlike many other pathogens in the United States because transmission, or even perceived exposure, remains criminalized in many states (see: https://www.lgbtmap.org/equality-maps/hiv_criminalization_laws). This legal context substantially heightens the risks of stigma, discrimination, and misuse of data.
• In parallel, there is an ongoing debate within the U.S. HIV advocacy community regarding the ethics of genomic surveillance. In many jurisdictions, routinely collected clinical samples are sequenced without explicit consent for genomic surveillance. Extending such approaches to wastewater data without broad community input risks amplifying mistrust.
• The omission of this broader ethical discussion is a major limitation. I recommend reading Mehta et al 2019 Coltart et al 2018 as starting references on the ethical dimensions of pathogen genomics and surveillance. This would provide important context on issues of consent, equity, and responsible data use.
3. Utility of wastewater HIV detection
• I remain unconvinced that wastewater detection of HIV offers actionable surveillance value. The authors suggest it could help guide testing campaigns, but what levels of detection would trigger an intervention? And what kind of intervention would realistically follow?
• A deeper discussion is needed on what thresholds of detection would be meaningful, how results could be operationalized, and whether such an approach would provide any advantage over existing epidemiologic data sources.
4. Consideration of HIV biology and shedding dynamics
• The introduction and discussion should more carefully address the biology of HIV fecal shedding. Who sheds HIV in stool, under what circumstances, and does this vary by strain or treatment status? Since not everyone sheds HIV via the gut, what biases could this introduce into surveillance frameworks relying on wastewater detection?
• To what extent can the authors infer whether detected signals represent multiple individuals or a single person shedding? Without such considerations, it is difficult to evaluate the validity of the proposed surveillance approach.

(Remarks on code availability)
Will be made public when published

Version 1:

Reviewer comments:

Reviewer #1

(Remarks to the Author)
The authors have done well to address my concerns. The primary limitation of the work remains, that of the recovery of HIV being dominated by a single site. Still I think the work represents a significant advancement in the science.
On figure 8: It's a rather small detail but I recommend the authors try a z-score on the prevalence to anonymize the sites. That's a bit simpler and easier to understand than dividing by the lowest rate.
On figure 8: Would the authors be more specific about the prevalence in the methods? Is this a period prevalence (what date range) or point prevalence (what date)? Is it prevalence of diagnosed HIV? Or prevalence of folks with HIV on ARVs? Lastly, taking only the WWTP zip code will introduce quite a bit of error. Do the authors have catchment areas for the WWTPs that they sampled? Can the authors estimate a median or mean HIV prevalence from the catchment area that is overlapping. There are a few different approaches to handle the mis-alligned geographies: Price M, Simpson BS, Tscharke BJ, Ahmed F, Keller EL, Sussex H, Kah M, Sila-Nowicka K, Chappell A, Gerber C, Trowsdale S. Reporting population size in wastewater-based epidemiology: A scoping review. Science of the Total Environment. 2024 Nov 25;953:176076.

(Remarks on code availability)

Reviewer #2

(Remarks to the Author)
I appreciate the detailed responses to my concerns regarding the use of the reference database and the ethical considerations. Overall, I am satisfied with the corresponding revisions, which I think substantially improve the manuscript. The authors were incredibly thoughtful and responsive to the reviewer critiques.

I have one remaining concern, which has left me skeptical about the public health utility of HIV waste surveillance data. In the revised Figure 8, the observed correlation appears to be driven largely by a single site (A2). I encourage the authors to conduct a sensitivity analysis, assessing the robustness of this association by evaluating the influence of A2 (i.e., re-estimating the correlation after excluding A2 and/or reporting standard influence diagnostics)

(Remarks on code availability)

Version 2:

Reviewer comments:

Reviewer #1

(Remarks to the Author)
The authors have addressed all my concerns.

(Remarks on code availability)

Reviewer #2

(Remarks to the Author)
I am happy with the sensitivity analyses provided in response to my remaining query. I recommend this article for publication.

(Remarks on code availability)

Open Access This Peer Review File is licensed under a Creative Commons Attribution 4.0 International License, which permits use, sharing, adaptation, distribution and reproduction in any medium or format, as long as you give appropriate credit to the original author(s) and the source, provide a link to the Creative Commons license, and indicate if changes were made.
In cases where reviewers are anonymous, credit should be given to 'Anonymous Referee' and the source.
The images or other third party material in this Peer Review File are included in the article's Creative Commons license, unless indicated otherwise in a credit line to the material. If material is not included in the article's Creative Commons license and your intended use is not permitted by statutory regulation or exceeds the permitted use, you will need to obtain permission directly from the copyright holder.
To view a copy of this license, visit https://creativecommons.org/licenses/by/4.0/

Reviewers' comments:

Reviewer #1 (Remarks to the Author):

The authors have conducted a wastewater-based epidemiology study of HIV, using genetic sequencing. They have found a large degree of an HIV signal is suspected to be driven by gene therapy or medical research. Whatever the source the vast majority of HIV recovered in wastewater was unrelated to HIV known to be circulating in the population. The results are quite interesting and have some important conclusions for the potential to use wastewater surveillance for HIV. I have a number of questions and suggestions.

1) In the introduction the authors frame the study as a complement to clinical HIV surveillance, with the ability to identify areas where HIV is circulating and maybe missed in diagnosis. The authors make inferences based upon the genetic makeup of the HIV recovered from wastewater, but they never show the clinical data of prevalence or estimated incidence of HIV between the sampling sites. Could the authors include some of that information, while maintaining the anonymity of the sites? This would go a long way in helping to interpret the results, particularly figure 5B that shows the HIV signal is predominantly from City A. Including the size of the population contributing to the sampling sites would be important as well.

We thank the reviewer for these important questions. To address each component clearly, we organize our response to each question as follows:

***1a) Clinical HIV data context**: "*Could the authors include some of that information, while maintaining the anonymity of the sites? This would go a long way in helping to interpret the results..."

*Author's response: This is a good suggestion. We have now added **Figure 8**, which correlates wastewater-derived HIV-1 reads with ZIP code-level HIV prevalence data from AIDSVu for Cities A and D (the two cities where such data was available).*

Key findings form the correlation analysis:

- *All HIV-1 reads (i.e. non-circulating and circulating reads) show moderate correlation with community HIV prevalence when Site A1 is excluded (Pearson's r = 0.74, p = 0.035, n=8 sites) (**Fig. 8A**).*
- *Filtering for circulating reads (i.e. reads with C/N ratio > 1, the ones we think are authentic HIV signal) **substantially improves correlation** (r = 0.89, p = 0.003, 95%*

*CI [0.51, 0.98], n=8 sites), demonstrating that our classification framework successfully isolates epidemiologically meaningful signal (**Fig. 8B**).*

- *Site A1, which covers major biomedical research infrastructure, exhibits extreme vector contamination (14:1 non-circulating: circulating read ratio). Despite this, when Site A1 is included in the circulating reads analysis, correlation remains statistically significant (r = 0.69, p = 0.038, n=9) (**Fig. 8C**), suggesting that our competitive alignment framework can extract authentic circulating signal even in heavily contaminated samples.*
- *Site A2, which shows a 1:3 non-circulating:circulating read ratio, is located in the **highest HIV-prevalence catchment** in our dataset according to AIDSVu, providing independent **orthogonal** validation.*

These correlations provide proof-of-concept that wastewater HIV-1 surveillance tracks with community HIV burden when vector-derived sequences are properly classified.

We preserved site anonymity by normalizing prevalence rates (dividing by the minimum observed rate) rather than reporting absolute values. One site serving a major transportation hub was excluded because the high ratio of transient:resident population would systematically bias comparisons between wastewater signal and residential ZIP code prevalence data.

We acknowledge important limitations of this analysis:

- *Small sample size (n=7-9 sites) limits statistical power*
- *Sewersheds imperfectly align with ZIP code boundaries; our catchments typically serve multiple ZIP codes with varying population densities and HIV-1 prevalence*
- *AIDSVu prevalence data are from 2022, while our sampling extended through 2024*
- *Only two cities had ZIP code-level data available; other cities had only county-level data that were too coarse for meaningful sewershed correlation*

Despite these constraints, the consistent positive correlation, including highly significant results when vector contamination is properly accounted for (p = 0.003), demonstrate that our method detects epidemiologically relevant HIV-1 signal. The substantial improvement in correlation when filtering for circulating reads (r = 0.74 to r = 0.89) validate the biological utility of our classification framework.

We have revised the manuscript to include the following:

Figure 8:

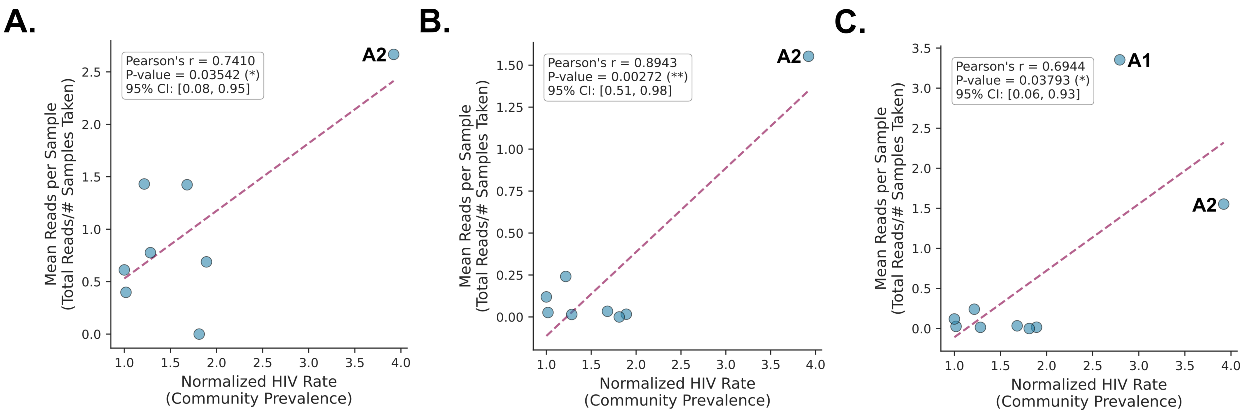

Figure 8. Correlation Between Wastewater-Derived HIV-1 Reads and ZIP Code Level HIV Prevalence*. Wastewater read counts (mean reads per sample, normalized by total number of samples taken) were corelated with publicly available HIV prevalence data from AIDSVu for Cities A and D. HIV prevalence rates were normalized by dividing by the lowest observed rate to preserve anonymity while maintaining proportional relationships. (****A****) Correlation using all HIV-1 reads with Site A1 excluded (n=8 sites). Pearson's r = 0.74, p = 0.035, 95% CI [0.08, 0.95]. (****B****) Correlation using only circulating reads (C/N ratio > 1) with Site A excluded (n=8 sites) to allow for consistent comparison with (****A****). Filtering for circulating reads substantially improved correlation strength (r = 0.89, p = 0.003, 95% CI [0.51, 0.98]) compared to all reads. (****C****) Correlation using circulating reads with Site A1 included (n=9 sites). Site A1, located at a major medical research center in the second-highest HIV-prevalence catchment, exhibited high non-circulating read contamination (14:1 non-circulating:circulating read ratio). Despite this contamination, correlation remained significant (r = 0.69, p = 0.038, 95% CI [0.06, 0.93]), demonstrating robustness of the competitive alignment framework for extracting circulating signal even in heavily contaminated samples. Site A2 represents the highest HIV-prevalence catchment in the dataset. Dashed lines represent linear regression fits. * p < 0.05, ** p < 0.01.*

Supplemental Figure 5 (sensitivity analysis):

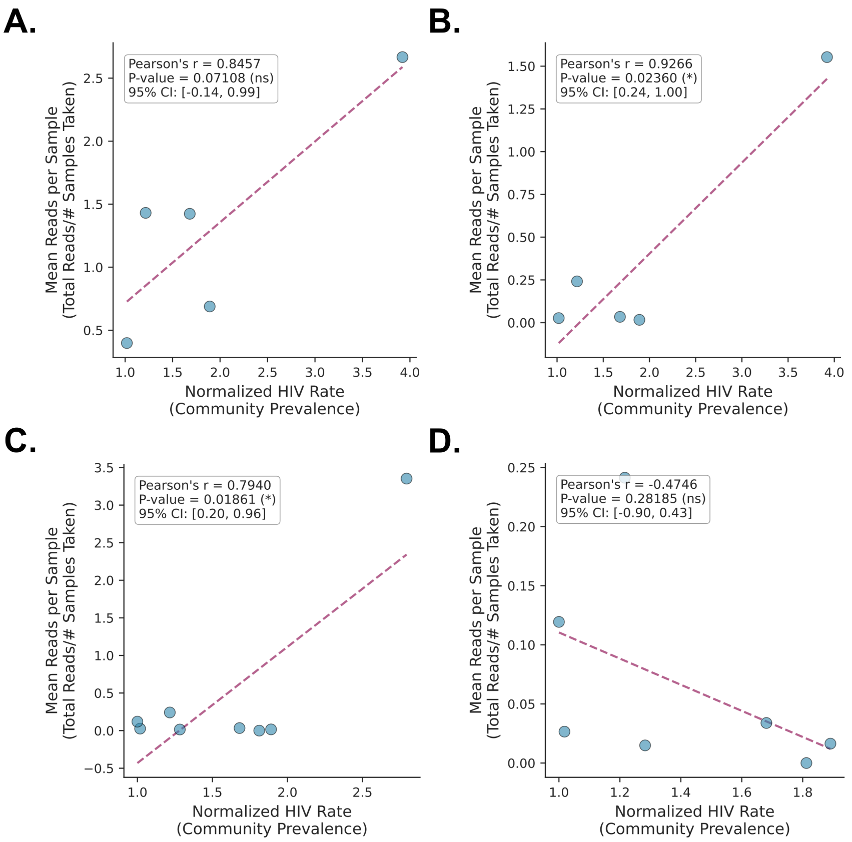

Supplemental Figure 5. Sensitivity Analysis Demonstrates Robustness and Dynamic Range Requirements*. City A analyzed independently (n=5) shows improvement from (****A****) all reads (r = 0.85, p =0.071, 95% CI [-0.14, 0.99]) to (****B****) circulating reads (r = 0.93, 0.024, 95% CI [0.24, 1.00]). Circulating read classification converts marginally significant trend into significant correlation, with confidence intervals shifting from including zero to excluding it. (****C****) Circulating reads correlation excluding Site A2 (highest prevalence HIV) but including Site A (highest vector contamination) remains significant (r = 0.79, p = 0.019, n=8). This demonstrates the relationship is not dependent on the highest-burden site and that classification framework successfully extracts circulating read signal even from heavily contaminated sites. (****D****) Excluding both sites with elevated prevalence (A1 and A2; normalized rates > 2.5) results in restricted range among remaining sites (all with normalized rates < 2) and non-significant correlation (r = -0.47, p = 0.28, n=7). This restricted range effect is consistent with fundamental statistical principles: correlation requires adequate variation in both variables. The significant correlation in all three scenarios with at least one elevated-signal site (panels in main Fig. 8 plus panel* ***C*** *here; all p < 0.05) validate the biological relationship between HIV burden and wastewater signal. Linear regression with Pearson correlation, two-tailed p-values, and 95% confidence intervals are shown. HIV prevalence data from AIDSVu with ZIP code level resolution. ns p>0.05; * p<0.05; ** p<0.01.*

We have added a new subsection to the Results:

Lines 333-377:

"Circulating HIV-1 Reads Correlate with ZIP Code Level HIV Prevalence

To evaluate whether reads classified as circulating reflect epidemiologically meaningful signal, we correlated wastewater-derived read counts with publicly available HIV prevalence data from AIDSVu for Cities A and D, the two cities which had ZIP code level data available for HIV prevalence. We compared correlations using either all HIV-1 reads or only reads classified as circulating (C/N ratio > 1) to assess whether our classification framework improves clinical correlation.

*Site A1 presents a unique analytical challenge: located at a major medical research center, this site exhibited overwhelming vector contamination with 5,631 non-circulating (vector-derived) reads versus only 409 circulating reads, a 14:1 ratio. For all-reads analysis, including Site A1 would render the correlation biologically uninterpretable, as the signal would be dominated by research-associated vectors rather than community shedding. However, for circulating-read analysis, Site A1 provides a critical test of whether our classification framework can successfully extract authentic HIV signal even in heavily contaminated environments. Circulating read classification substantially improved correlation with HIV prevalence compared to using all reads (**Fig. 8**). When Site A1 was excluded from both analyses (to enable fair comparison), all reads showed a moderate correlation (Pearson's $r = 0.74$, $p = 0.035$, 95% CI [0.08, 0.95], n=8) (**Fig. 8A**), while circulating reads demonstrated a substantially stronger relationship ($r = 0.89$, $p = 0.003$, 95% CI [0.51, 0.98], n=8) (**Fig. 8B**). The tighter confidence interval [0.51, 0.98] versus [0.08, 0.95] and improved p-value (0.003 vs 0.035) validate that filtering vector-derived sequences reveals stronger associations with clinical data. Critically, when Site A1 was included in the circulating reads analysis to test performance of our circulating read quantification framework, the correlation remained statistically significant ($r = 0.69$, $p = 0.038$, 95% CI [0.06, 0.93], n=9) (**Fig. 8C**). Although correlation strength decreased from $r = 0.89$ to $r = 0.69$ when the heavily vector-contaminated site was included, the persistence of significant correlation despite a 14:1 vector-to-circulating ratio at Site A1 validates the robustness of our competitive alignment framework. This demonstrates that our method can extract epidemiologically meaningful circulating signal even in challenging real-world scenarios where wastewater catchments serve major biomedical research facilities.*

*To further assess the robustness of these correlations, we performed additional sensitivity analyses (**Supplemental Figure 5**). When analyzed independently, City A (n=5) showed stronger correlation with circulating reads (r = 0.93, p = 0.024) than all reads (r = 0.85, p = 0.071) (**Supplemental Figure 5A-B**).*

*The correlation was not dependent on any single high-burden site. When Site A2 (the highest HIV-prevalence catchment) was excluded but Site A1 (the most vector-contaminated site) was retained, the correlation among circulating reads from both cities remained significant (r = 0.79, p = 0.019, n=8) (**Supplemental Figure 5C**). This demonstrates that the relationship persists across different site combinations and is not driven by a single high-leverage point.*

*However, adequate dynamic range in HIV prevalence is necessary to detect the correlation statistically. When both sites with elevated prevalence (A1 and A2; normalized rates > 2.0) were excluded, leaving only sites with uniformly low-to-moderate prevalence (normalized rates 1.0-1.9), the correlation became non-significant (r = -0.48, p = 0.28, n=7) (**Supplemental Figure 5D**). The significant correlations observed in all three scenarios that included at least one elevated signal site (r = 0.69-0.89, all p < 0.05) validate the biological relationship between community HIV burden and circulating wastewater reads."*

To the discussion we have added:

***Lines 586-623**: "Finally, linking wastewater sequencing results to clinical outcomes remains an open challenge. Correlating HIV-1 signal in wastewater with geographic incidence, treatment coverage, or viral suppression rates would enable more confident attribution of signal to community-level transmission. Such integration is both feasible and necessary: while wastewater provides granular, continuous surveillance data, its public health utility hinges on validation against established clinical metrics. To begin addressing this challenge, we performed an exploratory correlation analysis using publicly available HIV prevalence data from AIDSVu54 (Emory University), which aggregates data from the CDC's National HIV Surveillance System, for limited sites where ZIP code level data were available (Cities A and D, n=7-9 sites). This analysis provided an initial test for whether our circulating read classification tracks with known epidemiological patterns.*

Site A1, serving a major medical research center, provided a stringent test of our classification framework: with a 14:1 non-circulating:circulating ratio, we asked whether our method could extract authentic circulating signal when background contamination was high. The persistence of significant correlation when A1 was included (r = 0.69, p = 0.038) despite reduction from r = 0.89 without it demonstrates that competitive alignment successfully discriminates circulating reads even under high contamination. This has

practical implications: surveillance remains viable in catchments serving biomedical facilities, though site selection strategies or targeted vector depletion could improve signal quality.

*Several limitations exist: small sample sizes (n=7-9), spatial mismatch between sewersheds and ZIP codes (with catchments typically serving multiple ZIP codes with varying population densities), 2022 AIDSVu prevalence data rather than contemporaneous shedding metrics, and availability of only two cities. One site serving a major transportation hub was excluded because transient populations (travelers) greatly outnumber local population, thus making HIV prevalence rates unreliable and violating the assumption that wastewater signal reflects local community burden. Additionally, the range restriction analysis (**Supplemental Figure 5D**) illustrates that wastewater HIV surveillance requires meaningful variation in HIV burden for statistical detection. When restricted to compressed prevalence (normalized rates 1.0-1.9), correlation became undetectable despite consistent positive associations across the full range (1.0-4.0). This null result reflects insufficient statistical power from limited dynamic range rather than contradictory biology, indicating that surveillance will be most informative in heterogeneous settings or with sufficient catchments to capture prevalence variation.*

Despite these constraints, consistent positive correlations across multiple scenarios, including highly significant results under optimal conditions ($p = 0.003$) and persistent significance with high non-circulating read contamination ($p = 0.038$), provide proof-of-concept that wastewater HIV detection tracks with community burden when vector contamination is properly accounted for. The improved correlation using circulating versus all reads validates our classification framework's biological relevance and practical utility."

And, finally, we have added a new subsection to the Methods:

***Lines 903-922**: "Correlation of Wastewater Signal with HIV Clinical Prevalence Data*

HIV prevalence data at ZIP code resolution were obtained from AIDSVu (https://aidsvu.org)[9], a publicly accessible database maintained by Emory University's Rollins School of Public Health that visualizes data from the CDC's National HIV Surveillance System. ZIP code level prevalence estimates from 2022 for Cities A and D were extracted in October 2025. For each site, data were taken from the ZIP code in which the corresponding wastewater treatment plant (WWTP) is located, as incomplete overlap between WWTP catchments and ZIP code boundaries limit the precision of broader geographic mapping. This approach provides a consistent and representative proxy for the contributing populations. To maintain anonymity, prevalence rates were normalized by dividing each value by the lowest prevalence rate among the sites analyzed. This indexing

approach preserved the proportional relationship between values for accurate statistical analysis without exposing actual rates that could enable identification of specific locations. Wastewater read counts were standardized by the number of samples taken per site and reported as "mean reads per sample" to account for variable sampling frequency across sites. One site in City A serving a major transportation hub was excluded from the correlation analyses due to high transient:resident population ratios that would systematically bias comparisons between wastewater signal and residential ZIP code prevalence data. Pearson correlation coefficients with two-tailed p-values and 95% confidence intervals were calculated using Python scipy (v. 1.16.3) to assess associations between normalized HIV prevalence and wastewater read counts."

1b) Site A interpretation: *"*...particularly figure 5B that shows the HIV signal is predominantly from City A*"*

Author's response: The reviewer correctly identifies that Site A1 does dominates our signal and requires explanation. In the manuscript, we note that this site is located near major medical research infrastructure:

Lines 388-389 *(revised manuscript line 505): "...given Site A1's proximity to major medical research infrastructure."*

Additionally, the original manuscript had:

Lines 393-398*: "Because lentiviral vectors are subject to strict regulatory disposal protocols, they are not typically considered a serious source of confounding HIV-1 signal in wastewater. This assumption is reasonable and has likely contributed to the lack of vector screening in prior studies. However, our results suggest that this assumption does not hold in practice. The consistent, high-abundance detection of vector-derived sequences across multiple time points and sites indicates persistent environmental input—likely associated with routine research activity—**given that these sequences were predominantly recovered from sites near major medical research infrastructure**. This overlooked confounder may help explain why HIV-1 wastewater detection has historically lagged behind surveillance of other pathogens."*

This has been revised to:

Lines 506-517*: "Because lentiviral vectors are subject to strict regulatory disposal protocols, they have not typically been considered a source of confounding HIV-1 signal in wastewater. This assumption has likely contributed to the lack of vector screening in prior studies. However, our results indicate that vector-derived nucleic acids are consistently detectable in wastewater, particularly at sites near biomedical research infrastructure. It is not possible to determine from sequence data alone whether these represent intact but*

inactivated vectors, fragmented genetic material, or degraded nucleic acids persisting after standard decontamination. Accepted decontamination protocols prioritize inactivation of infectious agents over elimination of all nucleic acids, and nucleic acid persistence after chemical or thermal treatment is well-documented.[51] The consistent recovery of vector sequences across multiple time points at research-adjacent sites suggests this reflects research activity rather than isolated contamination event."

*We have also added an earlier sentence in the results at revised manuscript **lines 103-104**": "This concentration of signal at Site A1, located near major medical research infrastructure, is consistent with predominant detection of vector-associated sequences (see below)."*

The high signal at this site, which is overwhelmingly non-circulating/vector-associated, is consistent with its proximity to research and clinical facilities that use lentiviral vectors. While we are limited in how many additional details we can provide without compromising site anonymity, this spatial pattern reinforces our finding that distinguishing circulating from non-circulating HIV sequences is critical for wastewater-based surveillance interpretations.

1c) Population size information: "Including the size of the population contributing to the sampling sites would be important as well."

Author's response: We are limited in how much additional detail we can provide without compromising the anonymization of our sampling sites, particularly given that this work involves HIV surveillance with attendant privacy and stigma concerns. We acknowledge this limitation affects our ability to contextualize the geographic distribution of our findings.

Revised Discussion:

***Lines 560-562**: "Additionally, the need to maintain site anonymity, which is particularly important for HIV surveillance given ongoing stigma and criminalization, limits our ability to fully characterize catchment-level factors that may influence signal composition."*

2) Figure 5B raises a very large question – why is region 4 of the HIV genome so dominant in City B, where all the other regions of the genome are dominant in city A? Do the authors have any theories – it's quite perplexing. How many HIV positive samples are represented here?

Author's response: We thank the reviewer for this important observation and we address each question below:

2a) Genomic region distribution patterns: "Figure 5B raises a very large question – why is region 4 of the HIV genome so dominant in City B, where all the other regions of the genome are dominant in city A"

*Author's response: This is a very good point. The apparent regional specificity is likely due to stochastic variations from small sample sizes rather than biological differences in which genomic regions circulate at different sites. City B contributed substantially fewer circulating reads overall compared to City A (**see Supplemental Figure 4**), making the signal more susceptible to sampling variation. The small number of reads from City B limits our ability to draw strong conclusions about genomic distribution patterns in that location.*

2b) Sample representation: "How many HIV positive samples are represented here?"

*The genomic and geographic distribution patterns in **Figure 5B** and **5C** reflect differences in sample size and detection frequency across sites. For **Figure 5C**, reads from Site A1 were recovered on 25 different sampling dates over the study period, while reads from Site A2 came from only 4 samples collected across 2 years. This disparity in temporal sampling contributes to the uneven genomic coverage observed between sites.*

Manuscript revisions:

***Lines 205-208 originally read:** "Site-level analysis showed that two sampling locations, Site A1 and Site A2 within City A, contributed disproportionately to these high-coverage regions (Fig. 6B-C)."*

***Line 232-235 now read:** "Site-level analysis showed that two sampling locations, Site A1 and Site A2 within City A, contributed disproportionately to these high-coverage regions (Fig. 6B-C), with circulating reads recovered on 25 sampling dates from Site A1 and 4 sampling dates from Site A2 over the two-year study period."*

3) Thinking through the sample sizes here, can the authors clearly state the number and proportion of samples that were positive for HIV? I expect this in lines 85-86, here the authors should report basic HIV positive statistics by sampling site and across time. I see it in the supplemental figures. From Figure 1B, it looks like about half the sites had 1-2 detections and half the sites had a few more – closer to 6-7? Is that accurate? It would be good to clearly state this. Also, some of the signal in figure 1B is really low – for example site B5 only had two detections, one with 2 reads and one with 1 read. Whereas site A1 had reads up to 2,399. Site A1 is so different from the rest of the sites – why is that? Its number of reads is more than all the other sites combined. Would it make sense just to focus on Site A1 – would the authors still be able to make the same inferences? The other sites are very, very different in HIV recovery. This is a big limitation in the work – the results are

dominated by a single site but the analysis is presented as multiple sites. From Figure 1A it looks like two points the HIV reads were higher – could that be related to dumping in the site? Looking at supplemental figure 1, I think that belongs in the main manuscript. I recommend trimming it to remove the sites that began sampling in 2024.

Author's response: We thank the reviewer for these detailed observations and will address each point below:

3a) Basic detection statistics: *"…*can the authors clearly state the number and proportion of samples that were positive for HIV? I expect this in lines 85-86, here the authors should report basic HIV positive statistics by sampling site and across time*"*

Author's response: We have added clear detection statistics in the revised manuscript, which now reads:
Lines 93-103*: "Across 40 wastewater sites in 16 Texas cities sampled 2,086 times between May 2022 and December 2024, HIV-1 reads were detected in 123 (5.9%) of samples. Site-level analysis revealed that several locations contributed disproportionately to HIV-1 read counts (i.e. the number of sequence reads), with certain sites (e.g. Site A1) showing recurrent high-level detection across the study period (Fig. 2B). All sites sampled more than 66 times had at least one positive sample (total range of sampling: 1-142 samples per site; range for positive sites: 66-142 samples per site). Detection frequency varied substantially by site: 6 sites had equal to or less than 3% positive samples (range: 1.5-3.0%), 7 sites had between 3.1 and 5% positive samples, 3 sites had >5.1% positive samples over the study period (Fig 2). Site A1 accounted for the majority of HIV-1 signal, with reads detected in 37 of 122 (30.3%) separate sampling events and a maximum single-sample read count of 2,399."*

3b) Site-level detection patterns: *"*From Figure 1B, it looks like about half the sites had 1-2 detections and half the sites had a few more – closer to 6-7? Is that accurate? It would be good to clearly state this.*"*

Author's response: The reviewer's assessment is approximately correct, and we have added site-level statistics:

Lines 97-103*: "All sites sampled more than 66 times had at least one positive sample (total range of sampling: 1-142 samples per site; range for positive sites: 66-142 samples per site). Detection frequency varied substantially by site: 6 sites had equal to or less than 3% positive samples (range: 1.5-3.0%), 7 sites had between 3.1 and 5% positive samples, 3 sites had >5.1% positive samples over the study period (Fig 2). Site A1 accounted for the majority of HIV-1 signal, with reads detected in 37 of 122 (30.3%) separate sampling events and a maximum single-sample read count of 2,399."*

3c) Low-signal sites: *"*some of the signal in figure 1B is really low – for example site B5 only had two detections, one with 2 reads and one with 1 read.*"*

Author's response: We acknowledge that sites with very low read counts (such as Site B5, which we note is actually the same site as B4 due to a metadata error that has been corrected in the revised manuscript) provide limited interpretive value. These low-signal detections may reflect true level-level shedding, sporadic contamination, or stochastic sampling effects, and we do not draw strong epidemiological conclusions from them.

3d) Site A1 dominance and analytical approach: *"*site A1 had reads up to 2,399. Site A1 is so different from the rest of the sites – why is that? Its number of reads is more than all the other sites combined. Would it make sense just to focus on Site A1 – would the authors still be able to make the same inferences? The other sites are very, very different in HIV recovery. This is a big limitation in the work – the results are dominated by a single site but the analysis is presented as multiple sites.*"*

Author's response: The reviewer raises an important concern about Site A1's dominance of the overall HIV-1 signal. We address this through three complementary approaches: (1) explaining the biological basis for Site A1's high signal, (2) demonstrating that multi-site analysis enables critical methodological insight impossible with Site A1 alone, and (3) providing empirical validation through correlation with clinical HIV prevalence data.

Why Site A1 has such a high signal:
Site A1, located near major medical research infrastructure, ***exhibits overwhelming vector contamination*** *with 5,631 non-circulating reads versus only 409 circulating reads (a 14:1 ratio). This extreme imbalance explains why it dominates total read counts: the signal is predominantly research-associated rather than community-derived.*

Why multi-site analysis is essential:
Limiting analysis to Site A1 alone would obscure our central methodological finding: vector contamination is site-specific and predictable based on proximity to research infrastructure. Site A2 provides the critical counterpoint: despite being in the same city, it shows the opposite pattern (64 non-circulating reads vs 191 circulating reads, a 1:3 ratio. This contrast demonstrates that:

- *Circulating HIV can be detected with minimal vector signal when sites are not adjacent to research facilities*
- *Signal composition varies predictably with site characteristics*
- *Our competitive alignment framework can distinguish authentic virus from vector-derived sequences across diverse contamination scenarios*

Without multi-site comparison, we could not validate that the classification framework works across different contexts or establish that high non-circulating signal is infrastructure-specific rather than a systematic issue with the method. And, without a sequencing-based approach, we could ***not differentiate contamination from true circulating signal****.*

Site A1 can be included or excluded with valid results (empirical validation):

*The new correlation analysis (****Figure 8****) directly addresses whether Site A1's dominance undermines our conclusions. We tested three scenarios:*

1. *All reads, Site A1 excluded (****Fig. 8A****): Moderate correlation with HIV prevalence (r = 0.74, p = 0.035, n=8 sites). This demonstrates detectable signal across sites even without the highest-signal location.*
2. *Circulating reads, Site A1 excluded (****Fig. 8B****): Correlation remains significant (r = 0.69, p = 0.038, n=9 sites) despite A1's 14:1 vector contamination ratio. This is the critical finding: even including a heavily contaminated site, our classification framework extracts circulating signal that correlates with community HIV burden.*

The persistence of significant correlation when A1 is included (p = 0.038) demonstrates that our method is robust to contamination, precisely the scenario most relevant for real-world surveillance where sites may unavoidably be near research facilities.

Additionally, ***Supplemental Figure 5*** *shows sensitivity analyses confirming that correlation is not dependent on any single high-leverage site. When A2 (the highest HIV-prevalence catchment) is excluded but Site A1 (the second highest HIV-prevalence catchment and most vector-contaminated site) is retained, correlation among circulating reads from both cities remain significant (r = 0.79, p = 0.019, n=8).*

The revised Results now include:

Lines 340-361*: "Site A1 presents a unique analytical challenge: located at a major medical research center, this site exhibited overwhelming vector contamination with 5,631 non-circulating (vector-derived) reads versus only 409 circulating reads, a 14:1 ratio. For all-reads analysis, including Site A1 would render the correlation biologically uninterpretable, as the signal would be dominated by research-associated vectors rather than community shedding. However, for circulating-read analysis, Site A1 provides a critical test of whether our classification framework can successfully extract authentic HIV signal even in heavily contaminated environments. Circulating read classification substantially improved correlation with HIV prevalence compared to using all reads (****Fig. 8****). When Site A1 was excluded from both analyses (to enable fair comparison), all reads showed a moderate correlation (Pearson's r = 0.74, p = 0.035, 95% CI [0.08, 0.95], n=8) (****Fig. 8A****), while*

*circulating reads demonstrated a substantially stronger relationship (r = 0.89, p = 0.003, 95% CI [0.51, 0.98], n=8) (**Fig. 8B**). The tighter confidence interval [0.51, 0.98] versus [0.08, 0.95] and improved p-value (0.003 vs 0.035) validate that filtering vector-derived sequences reveals stronger associations with clinical data. Critically, when Site A1 was included in the circulating reads analysis to test performance of our circulating read quantification framework, the correlation remained statistically significant (r = 0.69, p = 0.038, 95% CI [0.06, 0.93], n=9) (**Fig. 8C**). Although correlation strength decreased from r = 0.89 to r = 0.69 when the heavily vector-contaminated site was included, the persistence of significant correlation despite a 14:1 vector-to-circulating ratio at Site A1 validates the robustness of our competitive alignment framework. This demonstrates that our method can extract epidemiologically meaningful circulating signal even in challenging real-world scenarios where wastewater catchments serve major biomedical research facilities."*

The revised Discussion now includes:

***Lines 597-604**: "Site A1, serving a major medical research center, provided a stringent test of our classification framework: with a 14:1 non-circulating:circulating ratio, we asked whether our method could extract authentic circulating signal when background contamination was high. The persistence of significant correlation when A1 was included (r = 0.69, p = 0.038) despite reduction from r = 0.89 without it demonstrates that competitive alignment successfully discriminates circulating reads even under high contamination. This has practical implications: surveillance remains viable in catchments serving biomedical facilities, though site selection strategies or targeted vector depletion could improve signal quality."*

3e) Temporal spikes in Figure 1A: *"*From Figure 1A it looks like two points the HIV reads were higher – could that be related to dumping in the site?*"*

Author's response: This is a very good observation, and we thank the reviewer for mentioning it. The temporal peaks in aggregate HIV-1 signal could potentially reflect episodic dumping events, increased community shedding, or sampling variation. However, the fact that elevated signal appears across multiple independent sites during these periods (as shown in Figure 1B (now Figure 2B in the revised manuscript) argues against site-specific dumping as the sole explanation. Without additional contextual data (e.g. facility discharge records), we cannot definitively attribute these peaks to a single cause.

3f) Supplemental Figure 1: "Looking at supplemental figure 1, I think that belongs in the main manuscript. I recommend trimming it to remove the sites that began sampling in 2024.*"*

Author's response: We agree and have moved Supplemental Figure 1 to the main manuscript as Figure 1, trimmed to remove sites that began sampling in 2024 as suggested.

4) The authors need to describe what a "read count" is and how it is used in a mixed sample context. They first use this term in line 88. The authors also need to describe their data beyond read count – what is the read length and depth of the sequencing that they obtained? From line 183 the authors state that more than 1/3 of the sequenced data could not map clearly to a reference set due to the short sequence length.

Author's response: This is good point. We provided clearer definitions and additional sequencing metrics in the revised manuscript.

4a) Definition of "read count": *"*The authors need to describe what a "read count" is and how it is used in a mixed sample context. They first use this term in line 88.*"*

Author's response: Read count refers to the number of sequence reads. In the context of wastewater, HIV-1 read counts reflect the relative abundance of HIV-1 genetic material detected in the sample, and this can be quantified using total filtered reads in the sample and genomic size of HIV-1 to calculate RPKMF, as in revised Figure 2B.

We have added a concise explanation to the first use of "read count" in the manuscript:

Lines 94-97 *now reads: "Site-level analysis revealed that several locations contributed disproportionately to HIV-1 read counts (i.e. the number of sequence reads), with certain sites (e.g. Site A1) showing recurrent high-level detection across the study period (Fig. 2B)"*

4b) Sequencing Metrics: *"*The authors also need to describe their data beyond read count – what is the read length and depth of the sequencing that they obtained?*"*

Author's response: Our samples were sequenced on Illumina platforms generating reads of approximately 150 bps. After quality trimming (Q≥25) and adapter removal with BBDuk, read lengths range from 30-150 bps, with reads <30 bps excluded from analysis.

To the revised manuscript we have added:

Lines 521-522 originally read: *"Reads were first trimmed using BBDuk (v38.84) to trim low-quality (Q < 20) reads and filter out <30 bp reads after trimming."*

Now reads:

Lines 732-734 now read*: "EsViritu-processed reads were first retrimmed using BBDuk (v38.84) to trim low-quality (Q < 20) reads and filter out <30 bp reads after trimming, resulting in read lengths of 30-151 bp (median=151 bp)."*

Coverage depth varied substantially by reference, and this can be seen in the coverage histograms of Figures 3, 5, 6, and 8. We have revised the legend of Figure 3 to include mean coverage depth.

Figure 3 legend original read: *"Figure 3. Genome-Wide Coverage of HIV-1 from Wastewater-Derived Reads via Hybrid-Capture Sequencing. Genome-wide read coverage from all EsViritu-classified HIV-1 reads aligned to the HXB2 reference genome (accession: K03455). Histogram (top) shows depth of coverage across the genome; stacked reads (bottom) indicate read distribution. Coverage spans 96.4% of the genome, with enrichment at LTR regions and the 3' end of env."*

Now it reads:

"Figure 3. Genome-Wide Coverage of HIV-1 from Wastewater-Derived Reads via Hybrid-Capture Sequencing. Genome-wide read coverage from all EsViritu-classified HIV-1 reads aligned to the HXB2 reference genome (accession: K03455). Histogram (top) shows depth of coverage across the genome; stacked reads (bottom) indicate read distribution. Coverage spans 96.4% of the genome, with a mean coverage depth of 136.4x, and enrichment at LTR regions and the 3' end of env."

4c) Unmapped reads: *"*From line 183 the authors state that more than 1/3 of the sequenced data could not map clearly to a reference set due to the short sequence length.*"*

The reviewer correctly notes that approximately one-third of EsViritu-classified HIV-1 reads could not be confidently assigned to specific reference genomes in our curated database. This was primarily due to short read length after quality trimming which limits the mappable sequence available for competitive alignment or potentially mischaracterization of reads by EsViritu in sensitive mode. These reads were excluded from circulating vs non-circulating classification analysis but were included in aggregate HIV-1 detection metrics shown in Figure 2 (Formerly Figure 1).

To the revised Results section we have added:

Lines 197-206: *"Out of the 16,710 total HIV-1 reads classified by EsViritu in sensitive mode, 10,737 (64%) aligned to one or more references in our curated genome set. Of these mapped reads, 780 (7.3%) aligned preferentially to contemporary LANL isolates (circulating), 7,265 (67.7%) aligned best to non-circulating reference strains, and 2,692 (25.1%) aligned equally well to both categories (shared; C/N = 1) (Fig. 5A). The remaining 5,973 EsViritu-classified reads (36%) did not map cleanly to any genome in the reference set, primarily due to short sequence length after quality trimming (median length: 151 bp) or mischaracterization by EsViritu in sensitive mode, which limits the mappable sequence*

available for competitive alignment. These unmapped reads were excluded from circulating vs. non-circulating classification and aggregate HIV-1 detection metrics shown in Figures 1-2."

5) In the introduction, lines 62-68 the authors should cite the studies that have already examined HIV through wastewater surveillance. It looks like they've cited them in the discussion as well – would be good in both places. For example:
- Wolfe MK, Varkila MR, Zulli A, Parsonnet J, Boehm AB. Detection and quantification of human immunodeficiency virus-1 (HIV-1) total nucleic acids in wastewater settled solids from two California communities. Applied and Environmental Microbiology. 2024 Dec 18;90(12):e01477-24.
- Terwilliger A, Clark J, Avadhanula V, Feliz K, Weesner K, Murphy E, Hopkins L, Kimata JT, Giordano TP, Piedra P, Maresso A. HIV Detection in Wastewater as a Potential Epidemiological Bellwether. Available at SSRN 4257909. 2022.
- Alshehri B, Birch ON, Greaves JC. Monitoring Multiple Sexually Transmitted Pathogens Through Wastewater Surveillance. Pathogens. 2025 Jun 5;14(6):562.

Author's response: thank you for bringing this to our attention. We have revised the introduction to include these references:

***Lines 66-68 now include**: "Early studies detected HIV-1 nucleic acids in wastewater as far back as the 1990s[20], and recent work has begun to explore HIV-1 detection using both PCR and sequencing-based approaches[21-23]."*

6) Line 69 – revise to "Nearly all viral WBE is performed using PCR."

Author's response: thank you for bringing this to our attention, it has been corrected in the revised manuscript.

7) Line 440-445 in the discussion – in the 1990s wastewater was tested for infectious HIV and it was not found to be infectious. The authors could cite some of those studies such as

Author's response: we thank the reviewer for suggesting this. It is a great addition to the manuscript.

***Lines 578-580 now read:** "Early work in the 1990s demonstrated that infectious HIV could not be recovered from wastewater, likely due to viral inactivation during treatment processes or environmental degradation.[20,48,52,53]"*

8) From supplemental figure 4 – does the incidence or prevalence of HIV support the disproportionate results by city here? I know the authors have stated this, but can they share any prevalence or incidence estimates while still preserving anonymity of the sites?

*Author's response: This question addresses the same core concern raised in Question 1 about correlating wastewater signal with clinical HIV data. Please see our response to Question 1a, which includes the comprehensive correlation analysis we have added (**Figure 8**) directly addressing this question.*

*To specifically answer the "disproportionate results by city" observation: City A dominates both total reads and circulating reads. The correlation analysis in **Figure 8** demonstrates that this is epidemiologically appropriate because both Site A1 and Site A2 are located in high HIV-prevalence catchments according to available AIDSVu data (A2 is the highest-prevalence catchment, A1 is the second-highest). While Site A1's total signal is dominated by vector contamination from adjacent research infrastructure (14:1 non-circulating:circulating ratio), the strong correlation between circulating reads and HIV prevalence across all sites ($r = 0.89$, $p = 0.003$, **Fig. 8B**) validates that City A's dominance reflects genuine differences in HIV burden across cities, not analytical artifacts.*

Reviewer #1 (Remarks on code availability):

I reviewed this as an epidemiologist.

Reviewer #2 (Remarks to the Author):

Thank you for the opportunity to review this manuscript, which evaluates a previously developed agnostic sequencing approach for wastewater surveillance to detect HIV-1. The article is generally well written and easy to follow. However, its conclusions are undermined by methodological limitations in the choice of reference database and by insufficient discussion of the ethical context. Addressing these issues, particularly by expanding sensitivity analyses and integrating a nuanced ethical discussion, would substantially improve the manuscript. I have several major considerations.
Major Comments
1. Methodological concern: reference database selection
• My major methodological concern relates to the curated reference database used to map circulating and non-circulating HIV strains. The authors employed 144 full-length subtype B

HIV-1 genomes. Given the growing number of infections linked to non-B subtypes in the United States, why were these excluded?
https://pubmed.ncbi.nlm.nih.gov/30961993/
https://pubmed.ncbi.nlm.nih.gov/28318764/
The choice of reference set is critical because it fundamentally shapes read-mapping efficiency, detection sensitivity, and ultimately all downstream analyses and inferences. For example, if a locally curated dataset of HIV sequences, including non-B subtypes, were used, it is possible the authors would have captured more reads and generated more accurate results. Excluding non-B references risks systematically underestimating signals from circulating lineages.
• Relatedly, there is seemingly low coverage observed in pol, the genomic region where most U.S. sequence data are historically derived for surveillance and clinical monitoring. If pol coverage in the wastewater samples was indeed poor, did this drive the authors to avoid using local reference datasets that would have relied heavily on pol alignments? If so, this limitation should be explicitly discussed, as it may have restricted the ability to compare wastewater-derived data with the largest available source of U.S. HIV sequences.

•Since one of the manuscript's central conclusions is that lentiviral vectors may be incorrectly disposed of, a claim with substantial public health and policy implications, additional sensitivity analyses using alternative reference sets are necessary. This would allow readers to assess how robust the findings are to different assumptions.
• Furthermore, given the seriousness of this claim, were regulatory authorities informed? If not, the authors should clarify how such findings should be responsibly communicated. I also recommend tempering the tone of the discussion to reflect the uncertainty introduced by reference database limitations.

Author's response: We thank the reviewer for these thoughtful comments. These are excellent points, and we should have explained our reasoning better in the manuscript. We will address each component below:

1a) Reference Database Selection: *"*My major methodological concern relates to the curated reference database used to map circulating and non-circulating HIV strains. The authors employed 144 full-length subtype B HIV-1 genomes. Given the growing number of infections linked to non-B subtypes in the United States, why were these excluded?
https://pubmed.ncbi.nlm.nih.gov/30961993/
https://pubmed.ncbi.nlm.nih.gov/28318764/
The choice of reference set is critical because it fundamentally shapes read-mapping efficiency, detection sensitivity, and ultimately all downstream analyses and inferences.

For example, if a locally curated dataset of HIV sequences, including non-B subtypes, were used, it is possible the authors would have captured more reads and generated more accurate results. Excluding non-B references risks systematically underestimating signals from circulating lineages.*”*

Author’s response: As the reviewer’s citations show, subtype B makes up more than 93% of infections in the United States. This was the primary consideration for focusing on subtype B references.

However, we directly tested whether non-B subtypes contribute to our wastewater signal. We performed competitive mapping using non-B subtype references (3 isolates for A, C, D, F1, G; 2 isolates for O, and 1 isolates for H, J, K, L, N, O, P, and U subtypes) alongside the 5 non-circulating B subtype isolates. These results showed that reads overwhelming mapped to the B: HXB2 (8,652 reads), NL4-3 (4,832 reads), Bru (4,494 reads), NY5 (954 reads), and YU-2 (489 reads). In contrast, no non-B subtype isolate captured > 98 reads, with the highest number of reads mapping to a C subtype isolate (accession: MW262771). This represents <1% of our HIV-classified reads, validating that our B-focused approach captures the dominant circulating signal. We have added this analysis as Supplemental Figure 1.

Revised Supplemental Figure 1:

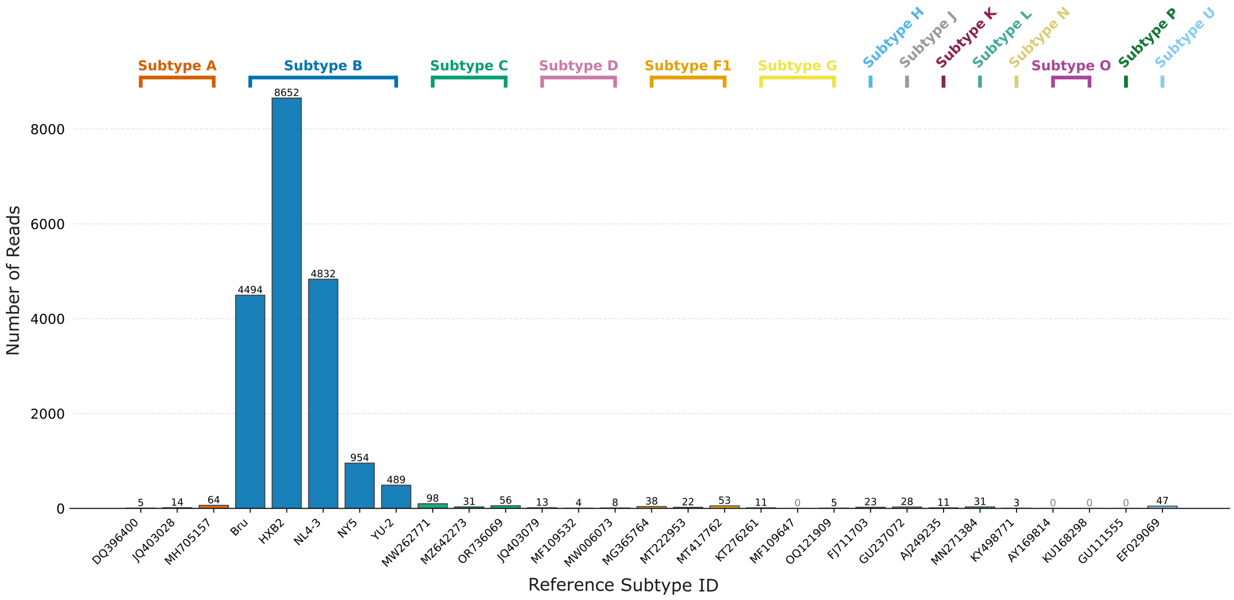

Supplemental Figure 1. *HIV-1 reads sampled in wastewater belong almost exclusively to Subtype B HIV-1. Number of reads mapping to 14 known HIV-1 subtypes, (3 isolates for A, C, D, F1, G; 2 isolates for O, and 1 isolate for H, J, K, L, N, O, P, and U subtypes) alongside the 5*

non-circulating B subtype isolates. Reference subtype ID or accession ID is present on the x-axis, with raw read count mapping to each reference on the y-axis.

1b) Pol coverage and local reference datasets: "Relatedly, there is seemingly low coverage observed in pol, the genomic region where most U.S. sequence data are historically derived for surveillance and clinical monitoring. If pol coverage in the wastewater samples was indeed poor, did this drive the authors to avoid using local reference datasets that would have relied heavily on pol alignments? If so, this limitation should be explicitly discussed, as it may have restricted the ability to compare wastewater-derived data with the largest available source of U.S. HIV sequences."

Author's response: The reviewer correctly notes that locally curated reference datasets could incrementally improve regional sensitivity. However, a core design principle of our study was developing a scalable, off-the-shelf surveillance platform deployable without site-specific customization. We used the commercial Twist Comprehensive Viral Research Panel, which enables immediate deployment in any location because of the many probes tiled across the viral genome that should increase detection of some nucleic acid fragments. While local curation might capture rare variants, it would require continuous updating as viral populations evolve, limit deployment to sites with existing sequencing infrastructure, increase cost and technical complexity, and complicate multi-site comparisons due to methodological heterogeneity. Our approach prioritizes deployability over site-specific optimization, similar to standardized SARS-CoV-2 wastewater assays. Given that non-B subtypes comprise <1% of our wastewater signal, this trade-off is justified.

Regarding pol *coverage specifically: our reference database selection was not driven by* pol *coverage limitations. We used whole-genome alignments because our hybrid-capture approach targets the complete HIV genome, and we felt genome-wide data would maximize information from our fragmented, low-coverage wastewater reads. The LANL database provided comprehensive subtype B representation (1 isolate per patient in the US, 2014-2024) appropriate for this untargeted sequencing approach.*

We have made the following revisions to the Discussion:

Lines 552-575 now read: *"This study demonstrates proof-of-concept for sequencing-based HIV wastewater surveillance but also reveals important technical challenges. The relatively low recovery of circulating-classified reads (780 of 16,710 total HIV reads, or ~5%) reflects both biological constraints, such as variable fecal shedding among people with HIV, and technical limitations of hybrid-capture efficiency for highly diverse viral genomes. On the technical side, hybrid-capture efficiency for diverse HIV fragments remains*

challenging, especially given the high sequence variability across global isolates. Biologically, the background presence of lentiviral vector DNA may obscure low-abundance authentic signals, particularly in sites near research institutions. Additionally, the need to maintain site anonymity, which is particularly important for HIV surveillance given ongoing stigma and criminalization, limits our ability to fully characterize catchment-level factors that may influence signal composition. Future optimization could explore enhanced enrichment strategies, deeper sequencing, or targeted depletion of vector-associated regions to improve signal-to-noise ratios. The limited depth of circulating reads in this dataset precludes high-resolution variant tracking or full-genome reconstruction, which may be essential for fine-grained epidemiological inference in future studies.

We deliberately employed commercially available reagents (Twist Comprehensive Viral Research Panel) and standardized reference databases to assess whether HIV surveillance could be achieved without site-specific customization. While this approach successfully detected and characterized circulating HIV signal, the sparse recovery suggests that further methodological development is needed before widespread implementation. Potential improvements include supplementing commercial panels with consensus sequences from high-prevalence regions or developing vector-specific blocking probes. The analytical framework established here, particularly the competitive alignment strategy for distinguishing circulating from vector-derived reads, provides a foundation for such optimization efforts."

***1c) Sensitivity analysis and robustness**: "*Since one of the manuscript's central conclusions is that lentiviral vectors may be incorrectly disposed of, a claim with substantial public health and policy implications, additional sensitivity analyses using alternative reference sets are necessary. This would allow readers to assess how robust the findings are to different assumptions.*"*

Author's response: The analysis described in 1a serves as a sensitivity analysis demonstrating that our findings are robust to reference set composition. The overwhelming dominance of subtype B signal (>99% of reads) indicates that inclusion of additional non-B references would not meaningfully alter our conclusions about circulating vs vector-derived signal composition.

***1d) Regulatory notification and discussion tone**: "*Furthermore, given the seriousness of this claim, were regulatory authorities informed? If not, the authors should clarify how such findings should be responsibly communicated. I also recommend tempering the tone of the discussion to reflect the uncertainty introduced by reference database limitations."

Author's response: We want to clarify an important point: we are detecting nucleic acids, not necessarily viable infectious vectors. Standard decontamination protocols prioritize inactivation of infectious agents but are not designed to eliminate all nucleic acids (PMID: 24388929). Commercial HIV wastewater surveillance assays already detect similar signals (e.g., GT Molecular), indicating this is established practice. Our contribution is providing the genomic resolution to distinguish circulating virus from research/clinical sources—a distinction impossible with PCR-only methods.

We have not notified regulatory authorities because our findings do not indicate biosafety violations or environmental health hazards. The persistent detection of vector-derived nucleic acids likely reflects normal research activity at sites with biomedical infrastructure, not necessarily improper disposal. However, we agree that tempering our discussion language is appropriate and have revised to clarify: (1) we cannot determine nucleic acid form or viability from sequence data alone, (2) nucleic acid persistence after decontamination is expected, and (3) vector-derived sequences represent a methodological consideration for surveillance interpretation rather than a safety concern.

We have made the following revisions to the Discussion:

***Lines 506-532 now read**: "Because lentiviral vectors are subject to strict regulatory disposal protocols, they have not typically been considered a source of confounding HIV-1 signal in wastewater. This assumption has likely contributed to the lack of vector screening in prior studies. However, our results indicate that vector-derived nucleic acids are consistently detectable in wastewater, particularly at sites near biomedical research infrastructure. It is not possible to determine from sequence data alone whether these represent intact but inactivated vectors, fragmented genetic material, or degraded nucleic acids persisting after standard decontamination. Accepted decontamination protocols prioritize inactivation of infectious agents over elimination of all nucleic acids, and nucleic acid persistence after chemical or thermal treatment is well-documented.[51] The consistent recovery of vector sequences across multiple time points at research-adjacent sites suggests this reflects research activity rather than isolated contamination event.*

The presence of vector-derived sequences represents an important methodological consideration for surveillance interpretation rather than necessarily indicating biosafety violations or environmental health hazards. This overlooked confounding factor may help explain why HIV-1 wastewater detection has historically lagged behind surveillance of other pathogens. Past studies relying on short, conserved PCR amplicons may have inadvertently amplified vector-derived sequences—particularly in regions like pol, env, and the LTRs—without recognizing their synthetic origin. In the absence of phylogenetic or genome-wide

context, such signals might reasonably be dismissed as laboratory artifacts, limiting the interpretability and dissemination of early findings.

It is worth noting that commercial HIV wastewater surveillance assays are already available and in use (e.g., GT Molecular), indicating that detection of HIV-related nucleic acids in wastewater, including potential vector-derived sequences, is an established practice. Our sequencing-based approach provides the genomic resolution needed to distinguish vector-derived from circulating signals, a distinction not possible with PCR-only methods, enabling more accurate interpretation of HIV wastewater surveillance data."

2. Ethical considerations

• The manuscript lacks sufficient discussion of the ethical issues surrounding wastewater surveillance for HIV. HIV is unlike many other pathogens in the United States because transmission, or even perceived exposure, remains criminalized in many states (see: https://www.lgbtmap.org/equality-maps/hiv_criminalization_laws). This legal context substantially heightens the risks of stigma, discrimination, and misuse of data

• In parallel, there is an ongoing debate within the U.S. HIV advocacy community regarding the ethics of genomic surveillance. In many jurisdictions, routinely collected clinical samples are sequenced without explicit consent for genomic surveillance. Extending such approaches to wastewater data without broad community input risks amplifying mistrust.

• The omission of this broader ethical discussion is a major limitation. I recommend reading Mehta et al 2019 Coltart et al 2018 as starting references on the ethical dimensions of pathogen genomics and surveillance. This would provide important context on issues of consent, equity, and responsible data use.

Author's response: We appreciate the reviewer raising these important ethical considerations and agree they warrant explicit discussion in the manuscript. We address each point below:

2a) HIV criminalization and stigma risks: *"*The manuscript lacks sufficient discussion of the ethical issues surrounding wastewater surveillance for HIV. HIV is unlike many other pathogens in the United States because transmission, or even perceived exposure, remains criminalized in many states (see: https://www.lgbtmap.org/equality-maps/hiv_criminalization_laws). This legal context substantially heightens the risks of stigma, discrimination, and misuse of data*"*

Author's response: We agree that the legal and social context surrounding HIV in the United States creates heightened ethical obligations for any surveillance approach. We will add a dedicated section addressing: (1) the heightened risks of stigma and misuse given HIV

criminalization laws in many U.S. states, (2) the critical distinction between population-level surveillance signals and individual genomics tracking, and (3) the need for community engagement in decisions about surveillance implementation.

We have made the following revisions to the Discussion:

***Lines 646-652 now read**: "We also cannot overlook the fact that HIV wastewater surveillance raises ethical considerations distinct from other pathogens. HIV remains subject to criminalization laws in many U.S. jurisdictions, and people living with HIV continue to face stigmatization and discrimination. There is ongoing debate within HIV advocacy communities about genomic surveillance conducted without explicit consent[54,65], and these realities demand that HIV surveillance approaches, including wastewater methods, include robust safeguards against misuse and harm to affected communities."*

***2b) Genomic Surveillance and community trust**: "*In parallel, there is an ongoing debate within the U.S. HIV advocacy community regarding the ethics of genomic surveillance. In many jurisdictions, routinely collected clinical samples are sequenced without explicit consent for genomic surveillance. Extending such approaches to wastewater data without broad community input risks amplifying mistrust.*"*

Author's response: We want to emphasize that our methodology does not currently enable, nor is it designed for, individual-level identification. The circulating HIV reads in our dataset are highly fragmented, represent pooled signal from entire sewershed populations, and reflect the substantial within-host genetic heterogeneity characteristic of HIV infections. Even with future technical improvements, the biological and methodological barriers to linking wastewater sequences to individuals remain formidable.

The reviewer should not that we have added an ethicist (Faith Fletcher) to the MS authors. She is our ethicist for this project and is consulted on a regular basis as the project develops. As part of our efforts, we have participated in town halls with community leaders and the community itself, and the lead PI Maresso has presented this project and work. The response has been overwhelmingly positive.

We also note that HIV wastewater surveillance is already occurring through commercial PCR-based detection kits (e.g. GT Molecular). Our sequencing-based approach provides greater genetic resolution, which does warrant additional ethical scrutiny, but the core concerns the reviewer raises apply to all forms of HIV wastewater surveillance. We will make clear in the revised manuscript that wastewater-derived sequences should not be used for individual identification or legal purposes, and that implementation of surveillance

programs should involve meaningful community input and be guided by principles aimed at preventing harm.

We have made the following revisions to the Discussion:

***Lines 653-667 now read**: "Our methodology does not enable individual-level identification. The circulating HIV reads we recovered are highly fragmented, represent pooled community signal from entire sewersheds, and reflect substantial within-host viral heterogeneity.[57] Even with future improvements in sensitivity, extensive biological and technical barriers to individual-level surveillance remain. Nevertheless, as sequencing-based wastewater surveillance capabilities advance, clear ethical frameworks are essential.[55,56] Wastewater-derived HIV data should focus exclusively on population-level public health benefits and should not be used for individual identification or legal purposes.*

We recommend that HIV wastewater surveillance programs partner with community-based HIV organizations and advocacy groups to address concerns about privacy, stigma, data stewardship and distrust.[58] Our team conducted townhalls with people living with HIV to gather input on study design, data use, and community concerns. These partnerships help ensure surveillance practices align with community needs and can advocate for expanding access to wastewater monitoring.[58] Continued community engagement is necessary to realize public health benefits while preventing harm to affected communities."

***2c) Ethical framework references**: "*The omission of this broader ethical discussion is a major limitation. I recommend reading Mehta et al 2019 Coltart et al 2018 as starting references on the ethical dimensions of pathogen genomics and surveillance. This would provide important context on issues of consent, equity, and responsible data use.*"*

Author's response: The suggested references (Mehta et al 2019 and Coltart et al 2018) provide valuable framing and we have incorporated them into our expanded ethics discussion.

3. Utility of wastewater HIV detection
• I remain unconvinced that wastewater detection of HIV offers actionable surveillance value. The authors suggest it could help guide testing campaigns, but what levels of detection would trigger an intervention? And what kind of intervention would realistically follow?
• A deeper discussion is needed on what thresholds of detection would be meaningful, how results could be operationalized, and whether such an approach would provide any advantage over existing epidemiologic data sources.

*Author's response: We appreciate the reviewer's critical assessment of actionable utility of our work. **Please view the response to reviewer 1 above**. While we agree that operational thresholds require further validation, our new correlation analysis (**Figure 8**) provides empirical evidence that wastewater HIV surveillance can detect epidemiologically meaningful signal when vector contamination is properly accounted for.*

***3a) Detection thresholds and intervention triggers**: "*I remain unconvinced that wastewater detection of HIV offers actionable surveillance value. The authors suggest it could help guide testing campaigns, but what levels of detection would trigger an intervention?*"*

*Author's response: Our exploratory correlation analysis (**Figure 8**, n=9 sites from 2 cities) provides proof-of-concept for intervention threshold development: circulating HIV-1 reads correlate with community HIV prevalence when vector contamination is properly accounted for (r = 0.89, p = 0.003 when excluding the heavily vector-contaminated Site A1; **Figure 8B**). This initial evidence demonstrates that detectable, epidemiologically relevant signal exists.*

*Defining specific threshold values will require longitudinal validation studies correlating wastewater measurements with clinical outcomes over time, similar to the development pathway for SARS-CoV-2 wastewater surveillance. However, our data establish the **foundation upon which such thresholds** can be built.*

*Importantly, the correlation analysis demonstrates practical robustness: even in heavily vector-contaminated samples (Site A1 with 14:1 non-circulating:circulating ratio), our classification framework extracts circulating signal that correlates with prevalence (r = 0.69, p = 0.038, **Figure 8C**). This suggests surveillance remains viable even in challenging real-world scenarios where catchments serve biomedical research facilities.*

*While precise threshold values require prospective validation studies, our correlation analysis suggests a workable approach: catchments with circulating read counts significantly elevated above site-specific baseline (adjusted for sewershed size and sampling frequency) would warrant targeted public health intervention. The correspondence between Site A2 (our highest circulating-signal site) and the highest HIV-prevalence catchment in the dataset (**Figure 8**) illustrates that this approach can identify priority areas.*

***3b) Operationalization and advantages over existing surveillance**: "*…what kind of intervention would realistically follow? A deeper discussion is needed on what thresholds of detection would be meaningful, how results could be operationalized, and whether such an approach would provide any advantage over existing epidemiologic data sources."

Author's response: Although our correlation analysis is exploratory and geographically limited (n=9 sites, 2 cities, using 2022 AIDSVu prevalence data), the strong observed correlation between circulating reads and community HIV prevalence ($r = 0.89$, $p = 0.003$) provides proof-of-concept that wastewater-derived signal tracks with disease burden. If validated in larger, prospective studies across diverse settings, this approach could offer several advantages over existing clinical surveillance:

1. *Coverage of undiagnosed populations: The correlation with diagnosed HIV prevalence from AIDSVu demonstrates that wastewater signal tracks with known disease burden. This is important because viral shedding occurs in both diagnosed and undiagnosed individuals with detectable viral loads. Therefore, wastewater surveillance has the potential to capture transmission from populations invisible to clinical reporting systems. An estimated 13% of people with HIV in the US are undiagnosed, and nearly 40% are not virally suppressed; these groups account for >80% of new transmission yet cannot be directly tracked by clinical reporting systems like those feeding AIDSVu.*
2. *Speed and continuous monitoring: wastewater provides continuous, population-level monitoring without requiring individuals to seek testing. This could enable detection weeks to months before traditional molecular epidemiological investigations identify transmission clusters, particularly important given that these investigations are lengthy and expensive.*
3. *Geographic granularity: Sewershed-level data can identify high-burden catchment areas that may be masked by coarser administrative reporting units. Site A2, a predominantly residential catchment with the highest circulating signal, corresponds to the highest HIV-prevalence area in our dataset, suggesting this approach may identify priority areas for intervention.*
4. *Resource efficiency: Unlike active case-finding or testing campaigns, wastewater monitoring leverages existing infrastructure with minimal additional manpower once established.*

Realistic intervention pathways, informed by our SARS-CoV-2 wastewater surveillance experience in Houston:

- *Deployment of mobile testing units to catchments with elevated circulating HIV signal*
- *Alerts to clinical facilities and providers in the affected areas to increase HIV testing and be vigilant for acute retroviral syndrome*
- *Intensified PrEP outreach and strategic resource allocation by local health departments*

- *Real-time monitoring of intervention effectiveness through continued wastewater sampling*

These interventions would target the critical gap in existing surveillance: identifying and reaching undiagnosed or virally unsuppressed populations who do not appear in clinical reporting systems yet drive transmission. The key advantage lies in detecting transmission among populations that traditional surveillance struggles to capture, potentially providing early warning before outbreaks are identified through clinical systems.

We acknowledge that demonstrating operational utility requires prospective validation studies including:

- *Longitudinal monitoring correlating wastewater trends with incidence data over time*
- *Multi-site validation across diverse geographic and demographic settings*
- *Pilot intervention studies assessing whether wastewater-guided responses improve outcomes*
- *Cost-effectiveness analyses comparing wastewater surveillance to existing approaches*

However, our proof-of-concept, particularly the robust correlation despite substantial vector contamination at Site A1, provides initial evidence that this approach shows promise and merits further development as a complementary surveillance tool.

We have made the following revisions to the Discussion:

Lines 586-645 now read: *"Finally, linking wastewater sequencing results to clinical outcomes remains an open challenge. Correlating HIV-1 signal in wastewater with geographic incidence, treatment coverage, or viral suppression rates would enable more confident attribution of signal to community-level transmission. Such integration is both feasible and necessary: while wastewater provides granular, continuous surveillance data, its public health utility hinges on validation against established clinical metrics. To begin addressing this challenge, we performed an exploratory correlation analysis using publicly available HIV prevalence data from AIDSVu54 (Emory University), which aggregates data from the CDC's National HIV Surveillance System, for limited sites where ZIP code level data were available (Cities A and D, n=7-9 sites). This analysis provided an initial test for whether our circulating read classification tracks with known epidemiological patterns.*

Site A1, serving a major medical research center, provided a stringent test of our classification framework: with a 14:1 non-circulating:circulating ratio, we asked whether our method could extract authentic circulating signal when background contamination was

high. The persistence of significant correlation when A1 was included ($r = 0.69$, $p = 0.038$) despite reduction from $r = 0.89$ without it demonstrates that competitive alignment successfully discriminates circulating reads even under high contamination. This has practical implications: surveillance remains viable in catchments serving biomedical facilities, though site selection strategies or targeted vector depletion could improve signal quality.

Several limitations exist: small sample sizes (n=7-9), spatial mismatch between sewersheds and ZIP codes (with catchments typically serving multiple ZIP codes with varying population densities), 2022 AIDSVu prevalence data rather than contemporaneous shedding metrics, and availability of only two cities. One site serving a major transportation hub was excluded because transient populations (travelers) greatly outnumber local population, thus making HIV prevalence rates unreliable and violating the assumption that wastewater signal reflects local community burden. Additionally, the range restriction analysis (Supplemental Figure 5D) illustrates that wastewater HIV surveillance requires meaningful variation in HIV burden for statistical detection. When restricted to compressed prevalence (normalized rates 1.0-1.9), correlation became undetectable despite consistent positive associations across the full range (1.0-4.0). This null result reflects insufficient statistical power from limited dynamic range rather than contradictory biology, indicating that surveillance will be most informative in heterogeneous settings or with sufficient catchments to capture prevalence variation.

Despite these constraints, consistent positive correlations across multiple scenarios, including highly significant results under optimal conditions ($p = 0.003$) and persistent significance with high non-circulating read contamination ($p = 0.038$), provide proof-of-concept that wastewater HIV detection tracks with community burden when vector contamination is properly accounted for. The improved correlation using circulating versus all reads validates our classification framework's biological relevance and practical utility.

Beyond validation, translating detection into actionable public health interventions requires further development. Empirical thresholds linking wastewater signal to community prevalence must be established through longitudinal studies, similar to those conducted for SARS-CoV-2, to determine meaningful alert levels. Our group's prior work with SARS-CoV-2 surveillance in Houston demonstrated the utility of wastewater-guided interventions.10,42 For HIV, the advantages could be even more substantial given the lengthy and expensive epidemiological investigations currently required to confirm local outbreaks.

Clear intervention pathways must also be defined in partnership with local health departments and affected communities. Plausible responses to elevated HIV signal could

include deployment of mobile testing units to affected catchments, alert to clinical facilities and providers in the affected areas to increase HIV testing and be vigilant for acute retroviral syndrome, intensified PrEP outreach, and strategic resource allocation. The key advantage lies in detecting transmission among undiagnosed populations—the groups most responsible for ongoing transmission—potentially weeks to months before traditional surveillance systems would identify an outbreak, and with minimal additional resources.

If successful, this approach could offer health systems a real-time, population-scale method to identify communities with high levels of undiagnosed or unsuppressed HIV-1—particularly in areas underserved by traditional surveillance. This study represents an early but critical step toward that goal, establishing an unbiased, scalable framework for detecting and evaluating HIV-1 signal through comprehensive wastewater virome sequencing. While substantial work remains to translate technical feasibility into operational readiness, the foundational analytical framework presented here provides a basis for future development."

4. Consideration of HIV biology and shedding dynamics

- The introduction and discussion should more carefully address the biology of HIV fecal shedding. Who sheds HIV in stool, under what circumstances, and does this vary by strain or treatment status? Since not everyone sheds HIV via the gut, what biases could this introduce into surveillance frameworks relying on wastewater detection?
- To what extent can the authors infer whether detected signals represent multiple individuals or a single person shedding? Without such considerations, it is difficult to evaluate the validity of the proposed surveillance approach.

Author's response: The reviewer raises an important consideration regarding differential HIV shedding patterns and their implications for wastewater surveillance. We address each point below:

*4a) HIV Shedding: "*The introduction and discussion should more carefully address the biology of HIV fecal shedding. Who sheds HIV in stool, under what circumstances, and does this vary by strain or treatment status?*"*

Author's response: Studies demonstrate that HIV RNA/DNA is detected in fecal samples from 8-19% of chronically infected individuals with detectable plasma viral loads, but not in subjects on antiretroviral therapy (ART) with undetectable plasma viral loads (PMID: 19799780). This shedding originates from gut-associated lymphoid tissue (GALT), a major HIV reservoir that releases infected cells and free virus into the intestinal lumen. We have added this biological context to the discussion.

The revised discussion now includes:

***Lines 465-474**: "Studies have shown that HIV RNA/DNA is detected in fecal samples from 8-19% of chronically infected individuals with detectable plasma viral loads, but is not detected in subjects on antiretroviral therapy (ART) with undetectable plasma viral loads.[47] This shedding originates from gut-associated lymphoid tissue (GALT), a major HIV reservoir that releases infected cells and free virus into the intestinal lumen. While this selectivity might initially seem problematic for surveillance, it actually enhances public health utility. Individuals with detectable viral loads, whether untreated, unsuppressed, or undiagnosed, represent the primary drivers of HIV transmission and are precisely the populations that traditional clinical surveillance struggles to capture."*

4b) Potential surveillance biases: "? Since not everyone sheds HIV via the gut, what biases could this introduce into surveillance frameworks relying on wastewater detection?*"*

Author's response: The reviewer correctly notes that not everyone sheds HIV via the gut and that this could introduce detection bias. However, this selectivity enhances rather than undermines surveillance utility: individuals with detectable viral loads, whether untreated, undiagnosed, or virologically unsuppressed, are the primary drivers of HIV transmission and the populations most likely to be missed by clinical surveillance.

Wastewater surveillance does not require universal shedding, only that sufficient signal is generated within the population served by the sewershed. The enrichment towards virally unsuppressed individuals aligns detections with public health need. This principle mirrors SARS-CoV-2 wastewater surveillance, which succeeded despite substantial variation in individual shedding patterns. We will add to the discussion, incorporating these findings and noting that future validation studies can further characterize the relationship between shedding patterns, treatment status, and detection sensitivity.

*4c) Individual vs population-level signal: "*To what extent can the authors infer whether detected signals represent multiple individuals or a single person shedding? Without such considerations, it is difficult to evaluate the validity of the proposed surveillance approach.*"*

Author's response: While HIV phylogenetics has been used forensically with high-quality clinical sequences, wastewater-derived reads present fundamentally different constraints: (1) extreme fragmentation (median length <150 bp after trimming), (2) pooled signal from entire sewersheds, (3) within-host viral diversity that exceeds between-host diversity in wastewater context, and (4) mixture with vector sequences. Even with improved sequencing depth, these biological and technical barriers make individual level attribution from wastewater infeasible with current methods. Fortunately, population-level

*surveillance is the intended application and our correlation analysis (**Figure 8**) validates that aggregated signal tracks community disease burden, which is the appropriate scale for public health interventions.*

The revised Discussion now includes:

***Lines 475-484**: "WBE does not require universal or uniform shedding across infected individuals; it requires only that sufficient shedding occurs within populations served by the sewershed to generate detectable signal. The enrichment of wastewater detection towards virally unsuppressed individuals aligns surveillance with epidemiological need. This principle is supported by SARS-CoV-2 wastewater surveillance, which succeeded despite substantial individual variation in shedding by disease stage, treatment status, and symptom severity. Future validation studies correlating wastewater measurements with clinical data will help clarify detection sensitivity across different populations, but the fundamental principle remains: population-level signal enriched for transmission-competent infections provides actionable surveillance value."*

Response to Reviewers' comments:

Reviewer 1: "The authors have done well to address my concerns. The primary limitation of the work remains, that of the recovery of HIV being dominated by a single site. Still I think the work represents a significant advancement in the science."

Author's response: We thank the reviewer for their continued support of this work and for noting that it represents a significant advancement in the field. We have addressed each of the remaining suggestions below.

Reviewer 1, Comment 1: "On figure 8: It's a rather small detail but I recommend the authors try a z-score on the prevalence to anonymize the sites. That's a bit simpler and easier to understand than dividing by the lowest rate."

Author's response: We agree with the reviewer that this is a better way to present the data, and thank them for the suggestion. We have implemented this change: all prevalence values in Figure 8 and Supplemental Figure 5 have been converted to z-scores ($z = (x - \mu) / \sigma$) calculated across the sites included in each respective panel. This provides a more intuitive and statistically standard representation while maintaining site anonymity.

We have revised the figure legends and Method sections accordingly.

Reviewer 1, Comment 2: "On figure 8: Would the authors be more specific about the prevalence in the methods? Is this a period prevalence (what date range) or point prevalence (what date)? Is it prevalence of diagnosed HIV? Or prevalence of folks with HIV on ARVs?"

*Author's response: We appreciate this request and hope that we will be able to clarify for both the reviewer and subsequent readers. The prevalence data used in this study represented **year-end 2023 point prevalence of persons living with diagnosed HIV per 100,000 population**, as reported through the CDC's National HIV Surveillance System and disseminated via AIDSVu. These estimates capture individuals with a documented HIV diagnosis who were alive at the end of the calendar year, 2023. The data do not distinguish by antiretroviral treatment status or viral suppression, nor do they include estimates of undiagnosed infection.*

We have revised the Methods section to include this specification and added a note in the Discussion acknowledging that diagnosed prevalence likely underestimates total community HIV burden, particularly in communities with lower testing access, which may attenuate the observed correlations between wastewater signal and true prevalence.

Reviewer 1, Comment 3: "Lastly, taking only the WWTP zip code will introduce quite a bit of error. Do the authors have catchment areas for the WWTPs that they sampled? Can the authors estimate a median or mean HIV prevalence from the catchment area that is overlapping. There are a few different approaches to handle the mis-alligned geographies: Price M, Simpson BS, Tscharke BJ, Ahmed F, Keller EL, Sussex H, Kah M, Sila-Nowicka K, Chappell A, Gerber C, Trowsdale S. Reporting population size in wastewater-based epidemiology: A scoping review. Science of the Total Environment. 2024 Nov 25;953:176076."

Author's response: We thank the reviewer for this critical methodological point and for directing us towards Price et al. *(2024), which provides an excellent overview of approaches for estimating contributing populations in epidemiology.*

We have substantially improved our prevalence estimation by incorporating sewershed catchment boundaries from either utility-reported or EPA's National Sewershed Dataset modeled service area polygons. Using these boundaries, we performed a spatial intersection with U.S. Census Bureau ZCTA (ZIP Code Tabulation Area) shapefiles (2023 vintage) in GeoPandas. For each sewershed, we computed a ***population-weighted mean HIV prevalence*** *by weighting each overlapping ZCTA's AIDSVu prevalence estimate by the fraction of its population falling within the catchment boundary, estimated via areal interpolation assuming uniform population distribution within each ZCTA. This approach replaces the single WWTP ZIP code with a composite estimate that accounts for multiple communities contributing wastewater to each treatment plant.*

We acknowledge that areal interpolation assumes uniform population distribution within the ZCTAs, which is an approximation. However, this approach represents a substantial improvement over the single ZIP code proxy and aligns with the recommendation of Price et al. *(2024). The Methods section has been revised to describe this procedure, and Figure 8 and Supplemental Figure 5 now reflect the updated prevalence estimates.*

Additionally, during revision, updated AIDSVu data became available for an additional City (City B), which we incorporated to expand the analysis from 3 to 4 cities (n=12 sites) and improve statistical power.

Finally, our initial revision mistakenly listed the H1 Site as belonging to City A. This is not entirely accurate. City H is within the metropolitan area of City A, but the Site was listed and masked separately as City H. We still included this in the Supplemental Figure 5A and 5B analysis, but the distinction has now been explicitly stated.

Reviewer 2: "I appreciate the detailed responses to my concerns regarding the use of the reference database and the ethical considerations. Overall, I am satisfied with the corresponding revisions, which I think substantially improve the manuscript. The authors were incredibly thoughtful and responsive to the reviewer critiques."

Author's Response: We are grateful for the reviewer's positive assessment of our prior revisions and for their thoughtful engagement with the public health implications of this work. We take the concern about Site A2's influence seriously and have performed a comprehensive sensitivity analysis to address it.

Reviewer 2, Comment 1: "I have one remaining concern, which has left me skeptical about the public health utility of HIV waste surveillance data. In the revised Figure 8, the observed correlation appears to be driven largely by a single site (A2). I encourage the authors to conduct a sensitivity analysis, assessing the robustness of this association by evaluating the influence of A2 (i.e., re-estimating the correlation after excluding A2 and/or reporting standard influence diagnostics)."

Author's response: We agree that rigorously evaluating the influence of individual sites, particularly A2 which occupies the upper extreme of both prevalence and wastewater signal distributions, is essential for interpreting the robustness of our analysis. We have explained our Site exclusion analysis in more detail and added Cook's distance analysis.

1. ***Site exclusion analysis****. Supplemental Figure 5C presents the circulating reads correlation after excluding Site A2 while retaining all other sites, including Site A1 (the most vector-contaminated catchment area). The correlation remains statistically significant ($r = 0.72$, $p = 0.013$, 95% CI [0.21, 0.92]), demonstrating that the association between wastewater HIV signal and community prevalence does not depend on Site A2 alone. We have revised the main text to reference this panel more prominently as a direct robustness check.*
2. ***Cook's distance analysis****. We computed Cook's distance for all sites in the full circulating reads analysis (Figure 8C, n=12). Two sites exceeded the conventional 4/n threshold (0.33): Site A2 (Cook's D = 1.90) and Site A1 (Cook's D = 1.17). No other site exceeded the threshold (all remaining Cook's D < 0.06). These two sites are influential for biologically distinct and interpretable reasons:*
    - ***Site A2*** *is influential due to high leverage, as it sits at the upper extreme of the prevalence distribution and falls approximately where the regression line predicts, anchoring the slope as the most informative observation in the dataset. This is the expected pattern when a genuine biological gradient*

exists but only one catchment represents the high-prevalence end of the range.

- ***Site A1*** *is influential for a different reason: it exhibits a large positive residual (standardized residual = 2.84), indicating that its circulating read count is substantially higher than predicted by its prevalence. This is consistent with residual vector contamination at this site, which serves a major medical center and exhibited a 14:1 non-circulating:circulating read ratio in our classification analysis. This is precisely the reason it was excluded from the Figure 8A all-reads analysis: while no other site had more than a mean of 5.75 reads per sample, Site A1 had 64.9 reads per sample. This explains why excluding A1 improves the correlation from r = 0.68 (Figure 8C) to r = 0.78 (Figure 8B), A1 introduces noise that attenuates the underlying relationship.*

3. ***Context for range restriction:*** *Supplemental Figure 5D shows that excluding both A1 and A2 results in a non-significant correlation (r = -0.13, p = 0.72). We interpret this as a range restriction effect rather than evidence against the biological relationship. With the two highest-prevalence catchments removed, the remaining sites span a narrow prevalence range, eliminating the statistical power needed to detect a trend. This is consistent with statistical principles and it is well-documented that SARS-CoV-2 WBE requires adequate dynamic range across communities to calculate a correlation.*

Across all sensitivity scenarios that retain sufficient dynamic range in HIV prevalence, the correlation between circulating wastewater reads and community HIV burden is consistently positive and statistically significant:

Analysis	***n***	***r***	***p-value***
Circulating reads, A1 excluded (Fig. 8B)	*11*	*0.78*	*0.005 (**)*
Circulating reads, all sites (Fig. 8C)	*12*	*0.68*	*0.016 (*)*
Circulating reads, A2 excluded (Supp. Fig. 5C)	*11*	*0.72*	*0.013 (*)*
Circulating reads, A1 and A2 excluded (Supp. Fig. 5D)	*10*	*-0.13*	*0.72 (ns)*

We believe that the consistency of significant positive correlations across the first three scenarios, combined with the biologically interpretable influence diagnostics, supports the conclusion that the wastewater-prevalence association is a robust finding rather than a single-site artifact. The loss of significance in the fourth scenario reflects insufficient dynamic range, not contradictory biology.

We have added Cook's distance results and their interpretation to the revised Results section and expanded the relevant Discussion paragraphs.

Summary of major revisions:

1. *Z-score standardization applied to all prevalence comparisons in Figures 8 and Supplemental Figure 5.*
2. *Prevalence data specified as year-end 2023 point prevalence of diagnosed HIV per 100,000 (CDC/AIDSVu)*
3. *Population-weighted catchment prevalence computed using EPA sewershed boundaries and Census ZCTA spatial overlay (2023 vintage), replacing single ZIP code estimates.*
4. *Comprehensive sensitivity analysis including site exclusion panels and Cook's distance influence diagnostics.*
5. *Expanded dataset incorporating an additional city (City B) with newly available AIDSVu data, and aggregation of co-located sampling points within the same sewershed (Sites D1 and D2).*
6. *Revised Discussion addressing geographic mismatch limitations, influence of high-prevalence sites, and a range restriction effect, with citation of Price et al. (2024).*
